# BIOME cohort: Multi‐system microbiome dynamics and clinical outcomes in massive burn injury patients

**DOI:** 10.1002/imt2.70106

**Published:** 2026-01-29

**Authors:** Runzhi Huang, Yixu Li, Xiaoliang Li, Xulin Chen, Gang Xu, Shurun Huang, Weixi Yang, Xiaodong Li, Haiming Xin, Jianqiang Jiao, Hui Chen, Yuguo Xie, Peng Duan, Wenjun Liu, Xiangdong Deng, Zongyu Li, Yiping Xiu, Jiansheng Zheng, Rujun Chen, Zhaohong Chen, Xingang Wang, Xiaojian Li, Gaoxing Luo, Yi Zhang, Hongyan Zhang, Shichu Xiao, Liang Xiao, Ruijin Guo, Jie Zhang, Xiaofei Ye, Hongtai Tang, Qiao Zuo, Bin Qu, Bingwei Sun, Changhong Lu, Chengliang Deng, Deyi Zheng, Geng Ji, Haisheng Li, Haizhou Yu, Hua Zhou, Jian Wu, Lanjun Nie, Lei Wang, Pihong Zhang, Qingfu Zhang, Shengwu Chao, Sisi Yang, Xianglin Ding, Xiangsheng Ding, Ying Yan, Jiangyong Shen, Yongjian Zhao, Yunchuan Pan, Yunbiao Shen, Yu Zhang, Zhihui Yao, Zhe Cheng, Jiangfan Xie, Zhigang Chu, Shijie Li, Long Xu, Xiuming Yang, Sujie Xie, Hanlin Sun, Shuyuan Xian, Dezhi Han, Maolong Dong, Chengde Xia, Peng Luo, Yongming Yao, Zhaofan Xia, Shizhao Ji

**Affiliations:** ^1^ Department of Burn Surgery The First Affiliated Hospital of Naval Medical University Shanghai China; ^2^ Research Unit of Key Techniques for Treatment of Burns and Combined Burns and Trauma Injury, Chinese Academy of Medical Sciences Shanghai China; ^3^ Department of Burn The First People's Hospital of Zhengzhou Zhengzhou China; ^4^ Department of Burns The First Affiliated Hospital of Anhui Medical University Hefei China; ^5^ Department of Burn and Plastic Surgery Northern Jiangsu People's Hospital Yangzhou China; ^6^ Department of Burns and Plastic Surgery The 910th Hospital of Joint Logistics Support Force of Chinese People's Liberation Army Quanzhou China; ^7^ Department of Burns and Plastic Surgery, Huai'an First People's Hospital Nanjing Medical University Huai'an China; ^8^ Department of Burn and Plastic Surgery Bethune International Peace Hospital Shijiazhuang China; ^9^ Department of Burns and Plastic Surgery The 924th Hospital of Joint Logistics Support Force of Chinese People's Liberation Army Guilin China; ^10^ Department of Burns The Fifth Hospital of Baoding City Baoding China; ^11^ Department of Burns Beijing Jishuitan Hospital Beijing China; ^12^ Department of Burn and Plastic Surgery Marine Corps Hospital Chaozhou China; ^13^ Burn Department General Hospital of TISCO Taiyuan China; ^14^ Department of Burn and Injury The Second Affiliated Hospital of Kunming Medical University Kunming China; ^15^ Department of Burns and Plastic Surgery The First People's Hospital of Xuzhou Xuzhou China; ^16^ Department of Burns Surgery The Fifth Hospital of Harbin Harbin China; ^17^ Department of Burn and Plastic Surgery General Hospital of Northern Theater Command Shenyang China; ^18^ Department of Burns and Plastic Surgery The 909th Hospital of Joint Logistics Support Force of Chinese People's Liberation Army Zhangzhou China; ^19^ Department of Burn The 906th Hospital of Joint Logistics Support Force of Chinese People's Liberation Army Wenzhou China; ^20^ Burn and Wound Repair Department Fujian Medical University Union Hospital Fuzhou China; ^21^ Department of Burns & Wound Care Center, The Second Affiliated Hospital Zhejiang University School of Medicine Hangzhou China; ^22^ Department of Burns and Plastic Surgery, Guangzhou Red Cross Hospital Jinan University Guangzhou China; ^23^ Institute of Burn Research, State Key Laboratory of Trauma and Chemical Poisoning, Southwest Hospital Third Military Medical University Chongqing China; ^24^ Department of Burn and Plastic Surgery Affiliated Hospital of Nantong University Nantong China; ^25^ Medical Center of Burn Plastic and Wound Repair, The First Affiliated Hospital, Jiangxi Medical College Nanchang University Nanchang China; ^26^ BGI Research Wuhan Hubei China; ^27^ State Key Laboratory of Genome and Multi‐omics Technologies, BGI Research Shenzhen China; ^28^ Tongji University School of Medicine, Tongji University Shanghai China; ^29^ Department of Health Statistics Naval Medical University Shanghai China; ^30^ Neurovascular Center, Changhai Hospital Naval Medical University Shanghai China; ^31^ Department of Burn Surgery Sichuan Academy of Medical Sciences & Sichuan Provincial People's Hospital Chengdu China; ^32^ Department of Burns and Plastic Surgery Suzhou Hospital Affiliated to Nanjing Medical University Suzhou China; ^33^ Department of Burns and Plastic Surgery Staff Hospital of Handan Iron and Steel Group Company Handan China; ^34^ Department of Burns and Plastic Surgery The Affiliated Hospital of Zunyi Medical University Zunyi China; ^35^ Department of Burn and Plastic Surgery Guizhou Provincial People's Hospital Guiyang China; ^36^ Department of Burn and Plastic Surgery Taizhou People's Hospital Taizhou China; ^37^ Department of Burn and Plastic Surgery The Fourth Affiliated Hospital of Nantong University Yancheng China; ^38^ Department of Burns Ganzhou Municipal Hospital of Jiangxi Province Ganzhou China; ^39^ Department of Burns and Plastic Surgery Gansu Provincial People's Hospital Lanzhou China; ^40^ Department of Burn and Plastic Surgery The Second Affiliated Hospital of Nanjing Medical University Nanjing China; ^41^ Department of Burns and Plastic Surgery Xiangya Hospital of Central South University Changsha China; ^42^ Burn and Wound Repair Center The Third Hospital of Hebei Medical University Shijiazhuang China; ^43^ Department of Burns and Plastic Surgery The Affiliated Hospital of Qinghai University Xining China; ^44^ Department of Burns and Plastic Surgery The General Hospital of Chinese People's Liberation Army Tibet Military Area Command Lhasa China; ^45^ Gansu Tianshui Xianglin Burn Specialty Hospital Tianshui China; ^46^ Department of Burns The Affiliated Lianyungang Hospital of Xuzhou Medical University Lianyungang China; ^47^ Department of Burns and Plastic Surgery Naval Hosplial of Eastern Theater Zhoushan China; ^48^ Department of Burns and Plastic Surgery General Hospital of Ningxia Medical University Yinchuan China; ^49^ Department of Burn and Plastic Surgery, Tianjin Fourth Hospital Nankai University Tianjin China; ^50^ Department of Burn & Skin Repair Surgery Hainan General Hospital (Hainan Affiliated Hospital of Hainan Medical University) Haikou China; ^51^ Department of Burns and Plastic Surgery Urumchi General Hospital of Lanzhou Command of Chinese People's Liberation Army Urumchi China; ^52^ Department of Burn The Second Hospital of the Southern Theater Command Navy Sanya China; ^53^ Department of Burns and Plastic Surgery The 926th Hospital of Joint Logistics Support Force of Chinese People's Liberation Army Kaiyuan China; ^54^ Department of Burns and Plastic Surgery The 928th Hospital of Joint Logistics Support Force of Chinese People's Liberation Army Haikou China; ^55^ Department of Burns Tongren Hospital of Wuhan University & Wuhan Third Hospital Wuhan China; ^56^ Department of Burn and Plastic Surgery The 969th Hospital of Joint Logistics Support Force of Chinese People's Liberation Army Hohhot China; ^57^ Department of Burn and Plastic Surgery The Third Affiliated Hospital of Naval Medical University Shanghai China; ^58^ Department of Oncology, Zhujiang Hospital Southern Medical University Guangzhou China; ^59^ Translational Medicine Research Center, Medical Innovation Research Division and The Fourth Medical Center of Chinese People's Liberation Army General Hospital Beijing China

## Abstract

Massive burn injuries (MBIs) are associated with high mortality and disability rates, primarily due to microbial‐driven sepsis from extensive skin barrier loss and subsequent scar formation. This makes MBI a compelling model for studying multi‐system microbial dysbiosis following skin barrier destruction. We launched the Burn & Trauma Injury Organisms and Microbiome Ecology (BIOME) project, planning to enroll at least 150 MBI patients from 50 hospitals across 28 provinces, autonomous regions, and municipalities in China. The cohort includes skin swabs, intestinal and oral samples, and longitudinal clinical data collected from within 72 h post‐injury through 1 year of follow‐up. This multicenter, prospective, longitudinal cohort will establish a comprehensive clinical microbiota resource for patients with MBI. By integrating multi‐system microbiome profiling with clinical outcomes, the BIOME project aims to elucidate the translational role of microbiota reconstruction as a potential target for diagnosis, prognosis, and therapeutic intervention in severe burn care.

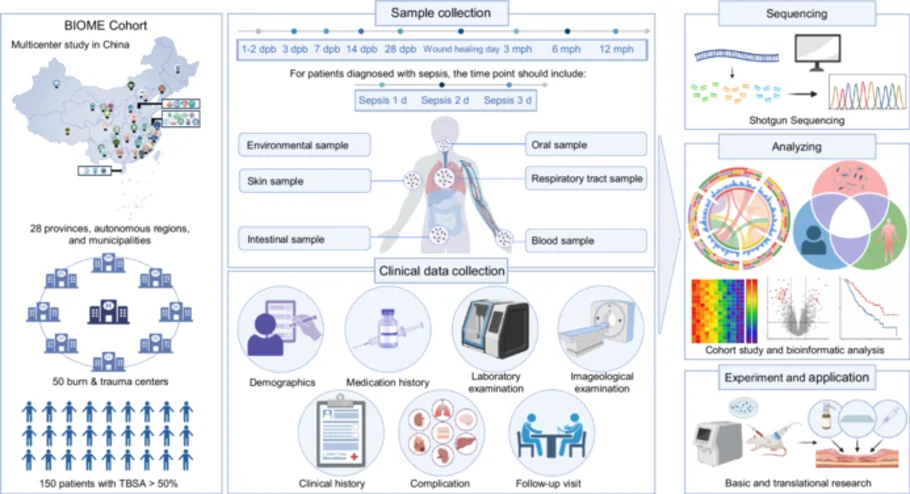

## AUTHOR CONTRIBUTIONS

**Runzhi Huang**: Conceptualization; methodology; software; data curation; supervision; formal analysis; validation; investigation; funding acquisition; visualization; project administration; writing—review and editing; writing—original draft. **Yixu Li**: Conceptualization; methodology; data curation; investigation; validation; supervision; resources; project administration; visualization; writing—original draft. **Xiaoliang Li**: Conceptualization; investigation; writing—original draft; methodology; validation; visualization; data curation; supervision; resources; project administration. **Xulin Chen**: Conceptualization; methodology; data curation; supervision; investigation; validation; project administration; resources; writing—review and editing. **Gang Xu**: Conceptualization; investigation; methodology; validation; writing—review and editing; project administration; resources; supervision; data curation. **Shurun Huang**: Data curation; supervision; resources; project administration; writing—review and editing; methodology; validation; conceptualization; investigation. **Weixi Yang**: Conceptualization; investigation; methodology; validation; writing—review and editing; project administration; resources; supervision; data curation. **Xiaodong Li**: Data curation; supervision; resources; project administration; writing—review and editing; validation; methodology; investigation; conceptualization. **Haiming Xin**: Conceptualization; investigation; methodology; validation; writing—review and editing; project administration; resources; supervision; data curation. **Jianqiang Jiao**: Data curation; supervision; resources; project administration; methodology; validation; investigation; conceptualization; writing—review and editing. **Hui Chen**: Project administration; writing—review and editing; validation; methodology; investigation; conceptualization; resources; supervision; data curation. **Yuguo Xie**: Data curation; supervision; resources; project administration; writing—review and editing; validation; methodology; investigation; conceptualization. **Peng Duan**: Conceptualization; investigation; methodology; validation; writing—review and editing; project administration; resources; supervision; data curation. **Wenjun Liu**: Data curation; supervision; resources; project administration; writing—review and editing; validation; methodology; conceptualization; investigation. **Xiangdong Deng**: Conceptualization; investigation; methodology; writing—review and editing; validation; project administration; resources; supervision; data curation. **Zongyu Li**: Data curation; supervision; resources; project administration; writing—review and editing; validation; methodology; investigation; conceptualization. **Yiping Xiu**: Conceptualization; investigation; methodology; validation; writing—review and editing; project administration; resources; supervision; data curation. **Jiansheng Zheng**: Data curation; supervision; resources; project administration; writing—review and editing; investigation; conceptualization; methodology; validation. **Rujun Chen**: Data curation; supervision; resources; project administration; writing—review and editing; validation; methodology; investigation; conceptualization. **Zhaohong Chen**: Conceptualization; investigation; methodology; validation; writing—review and editing; project administration; resources; supervision; data curation. **Xingang Wang**: Data curation; supervision; resources; project administration; writing—review and editing; methodology; validation; investigation; conceptualization. **Xiaojian Li**: Conceptualization; investigation; methodology; validation; writing—review and editing; project administration; resources; supervision; data curation. **Gaoxing Luo**: Data curation; supervision; resources; project administration; writing—review and editing; validation; methodology; conceptualization; investigation. **Yi Zhang**: Conceptualization; investigation; methodology; validation; writing—review and editing; project administration; resources; supervision; data curation. **Hongyan Zhang**: Data curation; supervision; resources; project administration; writing—review and editing; validation; methodology; conceptualization; investigation. **Shichu Xiao**: Conceptualization; investigation; methodology; validation; writing—review and editing; project administration; supervision; data curation; resources. **Liang Xiao**: Conceptualization; data curation; methodology; investigation; supervision; project administration; writing—review and editing; resources; validation. **Ruijin Guo**: Conceptualization; investigation; methodology; validation; writing—review and editing; project administration; resources; supervision; data curation; visualization; software. **Jie Zhang**: Data curation; supervision; resources; project administration; writing—review and editing; validation; methodology; conceptualization; investigation; software; visualization. **Xiaofei Ye**: Conceptualization; investigation; methodology; writing—review and editing; validation; software; data curation; supervision; resources; project administration; formal analysis; visualization. **Hongtai Tang**: Data curation; supervision; resources; project administration; writing—review and editing; investigation; conceptualization; methodology; validation. **Qiao Zuo**: Conceptualization; investigation; methodology; validation; project administration; resources; supervision; data curation; writing—review and editing. **Bin Qu**: Conceptualization; investigation; methodology; validation; writing—review and editing; project administration; data curation; supervision; resources. **Bingwei Sun**: Data curation; resources; supervision; project administration; writing—review and editing; validation; methodology; conceptualization; investigation. **Changhong Lu**: Conceptualization; investigation; methodology; validation; writing—review and editing; project administration; resources; supervision; data curation. **Chengliang Deng**: Data curation; supervision; resources; project administration; writing—review and editing; validation; methodology; conceptualization; investigation. **Deyi Zheng**: Conceptualization; investigation; methodology; validation; writing—review and editing; project administration; resources; supervision; data curation. **Geng Ji**: Data curation; supervision; resources; project administration; writing—review and editing; validation; methodology; conceptualization; investigation. **Haisheng Li**: Conceptualization; investigation; methodology; validation; writing—review and editing; project administration; resources; supervision; data curation. **Haizhou Yu**: Data curation; supervision; resources; project administration; methodology; validation; conceptualization; investigation; writing—review and editing. **Hua Zhou**: Methodology; validation; conceptualization; investigation; writing—review and editing; project administration; resources; supervision; data curation. **Jian Wu**: Data curation; supervision; resources; project administration; writing—review and editing; validation; methodology; conceptualization; investigation. **Lanjun Nie**: Conceptualization; investigation; methodology; validation; writing—review and editing; project administration; resources; supervision; data curation. **Lei Wang**: Data curation; supervision; resources; project administration; validation; writing—review and editing; methodology; conceptualization; investigation. **Pihong Zhang**: Conceptualization; investigation; methodology; validation; writing—review and editing; project administration; resources; supervision; data curation. **Qingfu Zhang**: Data curation; supervision; project administration; resources; writing—review and editing; validation; methodology; conceptualization; investigation. **Shengwu Chao**: Conceptualization; Investigation; methodology; validation; writing—review and editing; project administration; resources; supervision; data curation. **Sisi Yang**: Data curation; supervision; resources; project administration; writing—review and editing; validation; methodology; conceptualization; investigation. **Xianglin Ding**: Conceptualization; investigation; methodology; validation; writing—review and editing; project administration; resources; supervision; data curation. **Xiangsheng Ding**: Data curation; supervision; resources; project administration; writing—review and editing; validation; methodology; conceptualization; investigation. **Ying Yan**: Conceptualization; investigation; methodology; validation; writing—review and editing; project administration; resources; supervision; data curation. **Jiangyong Shen**: Data curation; supervision; resources; project administration; writing—review and editing; validation; methodology; conceptualization; investigation. **Yongjian Zhao**: Conceptualization; investigation; methodology; validation; writing—review and editing; project administration; resources; supervision; data curation. **Yunchuan Pan**: Data curation; supervision; resources; project administration; writing—review and editing; validation; methodology; conceptualization; investigation. **Yunbiao Shen**: Conceptualization; investigation; methodology; validation; writing—review and editing; project administration; resources; supervision; data curation. **Yu Zhang**: Data curation; supervision; resources; project administration; writing—review and editing; investigation; conceptualization; methodology; validation. **Zhihui Yao**: Conceptualization; methodology; validation; investigation; data curation; supervision; resources; project administration; writing—review and editing. **Zhe Cheng**: methodology; validation; conceptualization; investigation; writing—review and editing; data curation; supervision; resources; project administration. **Jiangfan Xie**: Data curation; supervision; resources; project administration; writing—review and editing; validation; methodology; conceptualization; investigation. **Zhigang Chu**: Conceptualization; investigation; methodology; validation; writing—review and editing; project administration; resources; supervision; data curation. **Shijie Li**: Data curation; supervision; resources; project administration; methodology; validation; conceptualization; investigation; writing—review and editing. **Long Xu**: Methodology; validation; conceptualization; investigation; data curation; supervision; resources; project administration; writing—review and editing. **Xiuming Yang**: Conceptualization; investigation; methodology; validation; writing—review and editing; project administration; resources; supervision; data curation. **Sujie Xie**: Data curation; supervision; resources; project administration; writing—review and editing; validation; methodology; conceptualization; investigation. **Hanlin Sun**: Conceptualization; investigation; methodology; validation; writing—review and editing; project administration; resources; supervision; data curation. **shuyuan xian**: data curation; supervision; resources; project administration; writing—review and editing; validation; methodology; conceptualization; investigation. **Dezhi Han**: Methodology; validation; conceptualization; investigation; data curation; supervision; resources; project administration; writing—review and editing; formal analysis; funding acquisition; visualization. **Maolong Dong**: Methodology; validation; conceptualization; investigation; supervision; data curation; resources; project administration; writing—review and editing; formal analysis; funding acquisition; visualization. **Chengde xia**: methodology; validation; conceptualization; investigation; writing—review and editing; data curation; supervision; resources; project administration; funding acquisition; formal analysis; visualization. **Peng Luo**: Data curation; supervision; resources; project administration; writing—review and editing; validation; methodology; conceptualization; investigation; visualization; funding acquisition; formal analysis. **Yongming Yao**: Methodology; validation; conceptualization; investigation; data curation; supervision; resources; formal analysis; project administration; visualization; funding acquisition; writing—review and editing. **Zhaofan Xia**: Writing—review and editing; project administration; formal analysis; data curation; supervision; resources; validation; visualization; methodology; conceptualization; investigation; funding acquisition. **Shizhao Ji**: Conceptualization; investigation; funding acquisition; writing—original draft; writing—review and editing; validation; visualization; methodology; software; formal analysis; project administration; data curation; supervision; resources. All authors have read the final manuscript and approved it for publication.

## CONFLICT OF INTEREST STATEMENT

The authors declare no conflicts of interest.

## ETHICS STATEMENT

The study was approved (No. CHEC2025‐071) by the Ethics Committee of the First Affiliated Hospital of Naval Medical University.

To the editor,

Burns and trauma are the leading causes of injury‐related mortality among individuals under 45 years of age worldwide [1]. Among these, massive burn injuries (MBIs), defined as burns involving >50% of total body surface area (TBSA) or full‐thickness burns affecting >20% TBSA, are associated with exceptionally high mortality and long‐term disability [2, 3]. The MBI patient mortality is mainly due to sepsis, which is caused by endogenous or exogenous microbial infection [4].

Growing evidence indicates a potential role of the microbiome in sepsis and its promise as a therapeutic and monitoring target [5, 6]. MBIs profoundly disrupt the human microbiota, including skin, gut, and respiratory communities [3, 7]. Dysbiosis of the skin microbiota contributes to the development and progression of dermatological disorders, whereas microbiome reconstruction facilitates wound healing by restoring skin barrier function [8, 9]. Beyond its local effects, the skin microbiome is tightly interconnected with the gut, oral, and respiratory microbiota, collectively forming a systemic microbial network that supports immune homeostasis and host defense [8, 9, 10]. Disruption of this network can promote pathogen overgrowth and immune dysregulation, thereby increasing susceptibility to severe infections, sepsis, and subsequent multiple organ dysfunction syndrome, ultimately leading to poorer clinical outcomes and prognosis [11, 12].

However, most studies remain limited to observational associations or animal models, typically focused on single niches at single time points [6, 8, 9]. The lack of large‐scale clinical cohorts capturing multi‐ecosystem dynamics limits mechanistic understanding and hinders intervention development [8, 9]. To address these gaps, we initiated the Burn & Trauma Injury Organisms and Microbiome Ecology (BIOME) cohort: a multicenter, longitudinal, prospective study investigating microbiome dynamics during MBI treatment.

The primary aims of the BIOME cohort are: (1) establishing a nationwide multicenter prospective cohort to investigate the longitudinal dynamic characteristics of multi‐system microbiomes (skin, gut, and oral) in MBI patients; (2) exploring the associations between dynamic microbial alterations and new‐onset sepsis and all‐cause mortality.

The secondary aims of the BIOME cohort include: (1) examining the relationships between microbiome dynamics and wound outcomes, focusing on wound healing trajectories and scar formation; (2) evaluating the predictive value of specific microbial signatures for clinical prognosis, aiming to identify early biomarkers for intervention.

## STUDY DESIGN

The BIOME cohort is a multicenter, prospective, longitudinal study conducted across 50 hospitals in 28 Chinese administrative regions (Figure 1A and Table S1). The participating centers are recognized for their expertise in managing MBIs. Participating centers cover all major geographical areas, ensuring national representation. To account for geographic microbiome variation, 20% of patients from geographical division of China will serve as a validation cohort [13]. The negative and positive control sampling of each center have been integrated into the study design.

**Figure 1:**
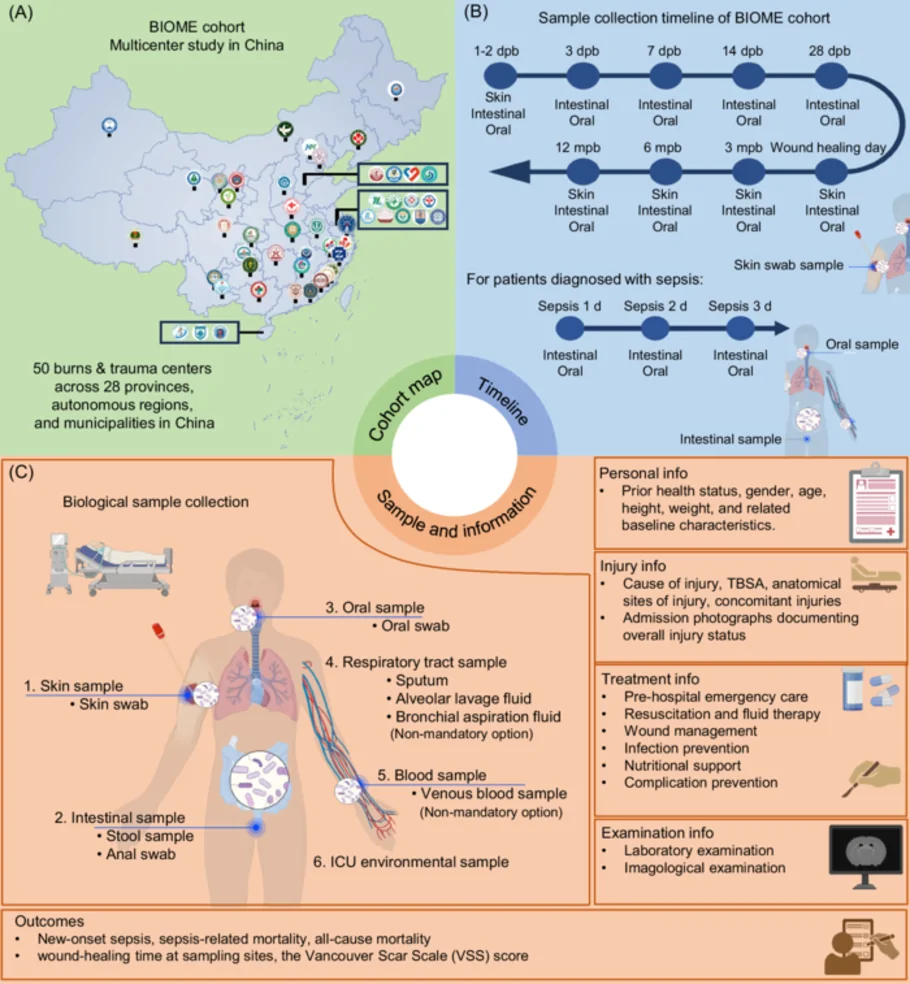
Study design and data collection framework of the BIOME cohort. (A) Cohort map. The BIOME cohort is a multicenter, prospective, longitudinal study conducted across 50 hospitals spanning 28 provinces, autonomous regions, and municipalities in China (red panels). (B) Unified Timeline and Sampling Strategy. A total of 150 patients with MBIs will be enrolled into a single unified cohort. For all participants, multi‐site biological sampling (skin, oral, and intestinal) is conducted longitudinally at standardized time points ranging from 1 to 2 ± 1 day post‐burn (dpb) through 12 months post‐burn (mpb). This comprehensive schedule covers the entire clinical trajectory, from the acute phase through wound re‐epithelialization to long‐term scar formation. Additionally, intensified daily sampling (1–3 days) is triggered immediately upon any clinical diagnosis of sepsis (blue panels). (C) Biological sample collection. Six categories of samples were obtained: (1) ICU environmental samples, (2) skin samples (swabs), (3) intestinal samples (stool, anal swab), (4) oral samples (oral swabs), (5) respiratory tract samples (sputum, alveolar lavage fluid, or bronchial aspiration fluid; optional), and (6) venous blood samples (optional) (left orange panels). Patient information. Collected metadata include: (1) personal information (prior health status, sex, age, height, weight); (2) injury information (cause, total body surface area, anatomical sites, concomitant injuries, admission photographs); (3) treatment information (pre‐hospital emergency care, resuscitation and fluid therapy, wound management, infection prevention, nutritional support, complication prevention); (4) examination information (laboratory and imaging assessments); and (5) outcomes (new‐onset sepsis, sepsis‐related mortality, all‐cause mortality, wound‐healing time at sampling sites, and scar quality assessed by the Vancouver Scar Scale (VSS)) (right orange panels).

The study was approved by the Ethics Committee of The First Affiliated Hospital of Naval Medical University (Approval No. CHEC2025‐071) and registered with the Chinese Clinical Trial Registry (ChiCTR2500099489). Comprehensive documentation including the study protocol, standard operating procedures (SOPs), and the statistical analysis plan were provided in the Supplementary Materials.

The BIOME cohort plans to enroll at least 150 MBI patients (Figure 1B). The planned sample size was justified using both event‐rate‐based and events‐per‐variable (EPV) considerations for the two primary outcomes: all‐cause mortality and new‐onset sepsis. A large disaster‐related MBIs' cohort from the 2014 Kunshan explosion (including 20 centers' data) reported an all‐cause mortality of 44.8% and a sepsis incidence of 68.5% in patients with TBSA > 50% [14].

For event‐rate‐based considerations, assuming a two‐sided significance level of *α* = 0.05 and a statistical power of: 1 – *β* = 0.80 (80%).

The minimum required sample size to detect a clinically meaningful difference in mortality and sepsis between microbiome‐defined subgroups was calculated to be 82 and 76 patients, respectively.

To ensure adequate statistical power for multivariable modeling, we applied the Events Per Variable (EPV = 10) principle to both primary outcomes. Based on the representative mortality rate of 44.8% and sepsis incidence of 68.5% from the Kunshan cohort, enrolling 150 patients is expected to yield:

150×44.8%≈67deaths,

150×68.5%≈103sepsisevents.

These event counts allow inclusion of 6−7 variables for mortality models and 10−11 variables for sepsis models, providing sufficient power to evaluate microbial predictors while adjusting for major clinical confounders. Therefore, the planned enrollment of 150 patients is statistically adequate for all primary analyses.

Multi‐system samples, including skin swabs, intestinal and oral samples, and control specimens, will be collected for shotgun metagenomic sequencing and analyzed with clinical outcomes (Figure 1C). This study is designed as a single, unified cohort. Skin, gut, and oral are all collected concurrently from the same set of patients to support integrated downstream analyses, rather than assigning participants to separate sub‐cohorts. Sample and data collection will occur at predefined time points: 1−2, 3, 7, 14, and 28 days (±1 day) post‐burn, wound healing day, and 3, 6, and 12 months (±7 days) post‐burn. Additional samples will be collected for 3 days following a sepsis diagnosis (ABA criteria) [15]. Demographic information, injury characteristics, treatment details, and outcomes/endpoints will be collected and recorded in the Electronic Data Capture (EDC) system of the Case Report Form.

## RECRUITMENT

Recruitment runs from July 1, 2025 to June 30, 2028. Patient enrollment, sepsis diagnosis, wound healing assessments, and key clinical outcomes will be jointly determined by two senior clinicians. One being the principal investigator at each participating center, and the other from The First Affiliated Hospital of Naval Medical University. If their evaluations differ, a third senior clinician from the independent adjudication committee will provide the final judgment. All enrollment decisions, diagnostic records, and wound assessment materials will be documented in the EDC system, and the independent adjudication committee will conduct a final review before data analysis and database lock. Written informed consent is mandatory.

Inclusion criteria for BIOME cohort study:

- Age 18−65 years;
- MBI patients with >50% TBSA who were admitted to the hospital within 72 h of burn injury;
- Patients or designated agents and guardians agreed to participate in this study and signed an informed consent form.

Exclusion criteria for BIOME cohort study:

- Patients with underlying endocrine disorders (e.g., diabetes mellitus, hyperthyroidism);
- Patients diagnosed with hematologic diseases or malignancies of the skin or internal organs;
- Patients with autoimmune diseases, immunodeficiency disorders, or immunosuppression (e.g., patients undergoing high‐dose immunosuppressive therapy);
- Patients with pre‐existing dermatologic conditions (e.g., psoriasis, vitiligo) in combination;
- Pregnant or lactating women;
- Any other conditions deemed by the investigators to potentially affect study outcomes.

## BIOLOGICAL SAMPLING PROCEDURES

Sampling corresponds to shock, infection, reparative, and rehabilitation phases of MBI at standardized time points. At each time point, comprehensive biospecimens and clinical data will be collected, including:

- Skin swab samples from burned and unburned regions of the torso and radial side of the extremities,
- Intestinal samples (fecal specimens or anal swabs),
- Oral swab samples,
- Demographic information, injury characteristics, treatment details, outcomes/endpoints.

To ensure consistency and minimize technical variability, all participating centers will use standardized nucleic acid preservation reagents, sample tubes, and sterile saline, which will be centrally distributed by The First Affiliated Hospital of Naval Medical University on a quarterly basis. In addition to the regular biological sampling, strict quality control measures, including the collection of environmental surface swabs, air blanks, reagent negative controls, and mock community positive controls, are mandatory in all participating centers to monitor contamination and validate sequencing accuracy. Detailed SOPs for sample acquisition and negative control handling are provided in the Supplementary Information. To ensure longitudinal continuity, the core sampling schedule will proceed as planned, irrespective of the patient's clinical setting (ICU, general ward, or outpatient). Sample collection will only be definitively terminated in the event of patient death, withdrawal of informed consent, or loss to follow‐up. To further reduce both technical variability and systemic error, all centers will employ standardized biospecimen collection kits, also distributed quarterly by The First Affiliated Hospital of Naval Medical University.

## PROGRESS OF THE COHORT

As of September 21, 2025, a total of 44 patients had been enrolled in the BIOME cohort, representing 29.3% of the target sample size of 150 cases (Figure 2). Patients are recruited from multiple regions of China, with 50.0% from the eastern region, 45.0% from the central region, and 5.0% from the western region (Figure 2A,B). The mean age of the enrolled patients was 43.6 ± 12.9 years, with 32 males (72.7%) and 12 females (27.3%) (Figure 2C).

**Figure 2:**
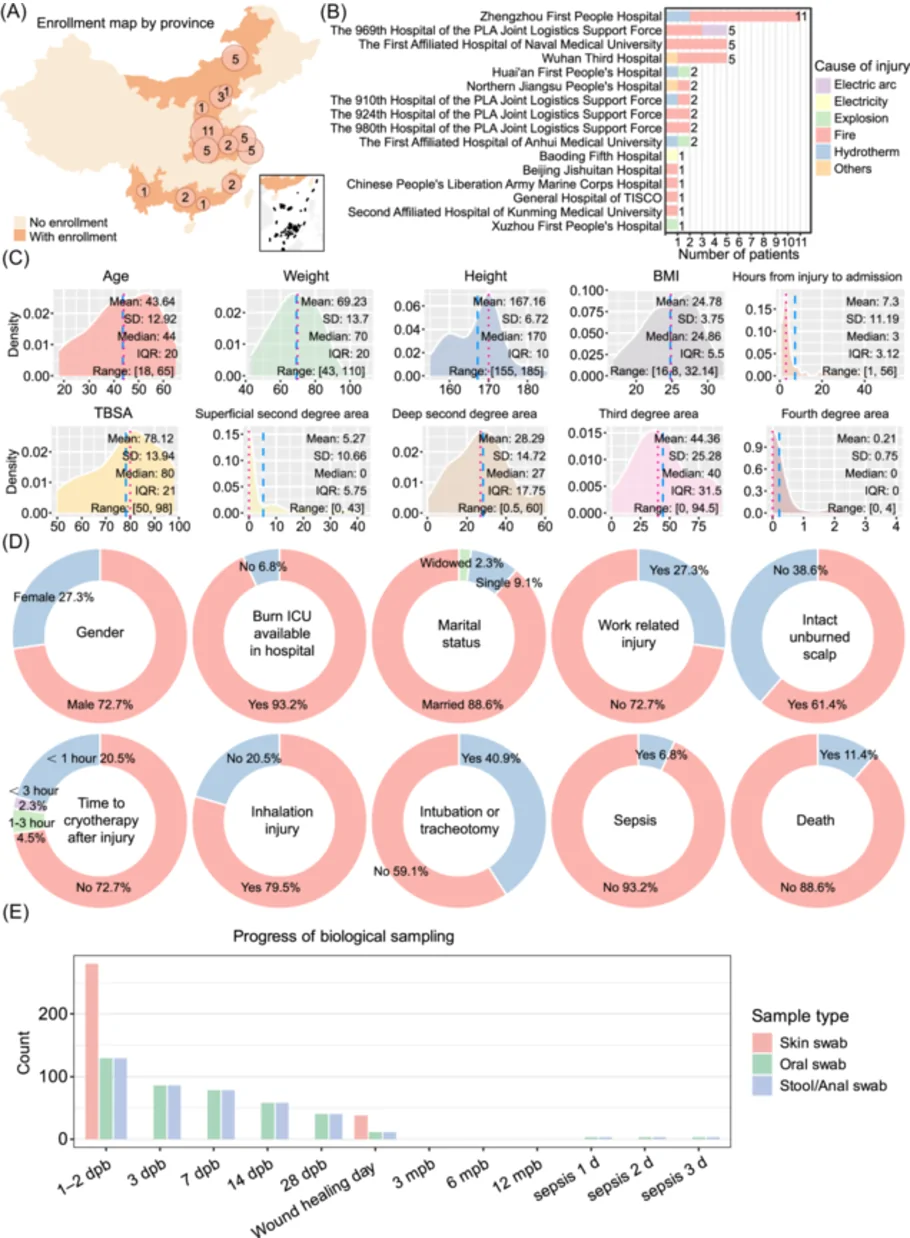
Sample Collection of the BIOME Cohort (as of September 21, 2025). (A) Enrollment map showing the geographic distribution of patients across provinces in China. Provinces with enrollment are shaded, with circle sizes proportional to the number of enrolled patients. (B) Recruitment hospitals and corresponding patient counts are shown in a horizontal bar plot. The inset donut chart summarizes the causes of burn injury, with fire being the predominant cause. (C) Distribution of continuous variables, including age, weight, height, BMI, hours from injury to hospital admission, TBSA, and burn depth area (superficial second‐degree, deep second‐degree, third‐degree, fourth‐degree). Density curves are overlaid with summary statistics (mean, SD, median, IQR, and range). (D) Donut charts illustrating categorical characteristics of the cohort, including gender, availability of burn ICU in the admitting hospital, marital status, work‐related injury, scalp integrity, time to cryotherapy after injury, presence of inhalation injury, airway interventions (intubation or tracheotomy), incidence of sepsis during hospitalization, and survival outcome. (E) Progress of biological sampling overview. Bar plots display the number of samples collected across time points.

Injury characteristics: the primary causes of injury were fire (70.5%), hydrotherm (11.4%), explosion (6.8%), electricity (4.5%), electric arc (2.3%), and other causes (4.5%). The mean body mass index (BMI) of the cohort was 24.78 ± 3.75, with a median of 24.86 (range: 16.8–32.14). The mean total burn surface area (TBSA) was 78.12 ± 13.94%, with a median of 80% (range: 50%−98%) (Figure 2C,D).

Early treatment information: The median time from injury to hospital admission was 3 h (interquartile range: 3.12; range: 0–56). Additionally, 61.4% of patients retained intact unburned scalp areas, and 79.5% presented with concomitant inhalation injury. Cryotherapy was initiated within 1 h after injury in 20.5% of patients, within 1−3 h in 2.3%, and after more than 3 h in 4.5%. Notably, 72.7% of patients did not receive any cryotherapy. Airway interventions (intubation or tracheotomy) were performed in 40.9% of patients during the acute phase (Figure 2C,D).

Outcome information: During hospitalization, 6.8% of patients developed sepsis. At follow‐up, the mortality rate was 11.4%, while 88.6% of patients survived (Figure 2D).

The progress of biological sampling is shown in Figure 2E.

## DISCUSSION

As a nationwide, multicenter, prospective cohort of patients with MBIs, the BIOME project was officially proposed on October 18, 2024.

The MBI represents a unique clinical model of systemic stress, ideal for investigating cross‐organ microbial interactions. A recent pivotal experimental study identified a “skin‐to‐gut axis,” demonstrating that dermal injury can directly disrupt the intestinal microbiome and immune homeostasis in mice [10]. The BIOME cohort extends these mechanistic insights into the clinical setting by enabling high‐resolution, longitudinal, multisystem profiling of the skin, gut, and respiratory microbiomes in human patients. Collectively, the BIOME project provides an unprecedented clinical resource for elucidating microbiome‐mediated mechanisms underlying post‐burn complications, including sepsis, and for identifying microbial biomarkers with diagnostic and prognostic potential.

Despite these strengths, we acknowledge certain limitations. First, regarding patient inclusion and demographics, potential biases in geographical location and gender may limit generalizability. MBIs are predominantly caused by accidental events, such as industrial explosions and residential fires, which may result in uneven regional and gender representation. In addition, microbial signatures identified in this cohort may not be directly generalizable to populations with different ethnic, dietary, or environmental backgrounds [16, 17]. To mitigate regional bias, the BIOME cohort has been expanded to include 50 leading burn centers across 28 provinces, with ongoing efforts to increase recruitment from underrepresented western and northern regions. Second, concerning sampling methodology, MBI patients frequently suffer from paralytic ileus due to burn shock, and high‐dose opioid analgesics (especially the first 72 h), necessitating the use of anal swabs as a substitute. Third, regarding clinical treatment, the administration of antibiotics, comorbidities, and variations in treatment protocols introduce unavoidable confounding factors [18]. Disentangling disease‐induced dysbiosis from medication effects remains a universal challenge in critical care microbiome research [18]. To reduce these treatment and source heterogeneity, we apply established statistical frameworks, including Linear Mixed Models (LMM), Multivariate Association with Linear Models (MaAsLin2), and subgroup and sensitivity analyses to enhance the robustness of our findings [19]. Finally, as an observational cohort, the BIOME cohort is inherently limited in its ability to infer causality. While robust associations between microbiome features and clinical outcomes can be identified, mechanistic validation will require future experimental studies using animal models, organoids, or fecal microbiota transplantation approaches.

## Supporting information


**Table S1.** Medical centers and research institutions participating in BIOME cohort.

Supporting Information

## Data Availability

The data that support the findings of this study are available on request from the corresponding author. The data are not publicly available due to privacy or ethical restrictions. The datasets generated during the BIOME study have been securely deposited at the First Affiliated Hospital of Naval Medical University, in full compliance with ethical regulations and patient confidentiality agreements. Due to ethical and legal restrictions, participant‐level data are not publicly available. The data that support the findings of this study are available from the corresponding authors for research and scientific purposes upon submission of a research proposal. Upon approval, all data analysis must be conducted on a local server with protected access, in compliance with Personal Information Protection Law of the People's Republic of China (PIPL) and Regulations on the Administration of Human Genetic Resources of the People's Republic of China. Additionally, the aggregated data and scripts used in the study are saved in GitHub (https://github.com/runzhihuang2022/Huang_20251224_iMeta). Supplementary materials (methods, tables, graphical abstract, slides, videos, Chinese translated version, and update materials) may be found in the online DOI or iMeta Science http://www.imeta.science/.
