# Supplementary material for: BIOME cohort: Multi‐system microbiome dynamics and clinical outcomes in massive burn injury patients
Source: Imeta. 2026 Jan 29;5(1):e70106. doi: 10.1002/imt2.70106 (PMC13726635; doi:10.1002/imt2.70106)
Supplement: Supplementary file 1 [file IMT2-5-e70106-s001.docx]

**Supporting information to**

**BIOME cohort: Multi-system microbiome dynamics and clinical outcomes in massive burn injury patients**

**Running title**: BIOME: Multi-system microbiome in massive burn injury patients

Runzhi Huang^1,2#^, Yixu Li^1,2#^, Xiaoliang Li^3#^, Xulin Chen^4^, Gang Xu^5^, Shurun Huang^6^, Weixi Yang^7^, Xiaodong Li^8^, Haiming Xin^9^, Jianqiang Jiao^10^, Hui Chen^11^, Yuguo Xie^12^, Peng Duan^13^, Wenjun Liu^14^, Xiangdong Deng^15^, Zongyu Li^16^, Yiping Xiu^17^, Jiansheng Zheng^18^, Rujun Chen^19^, Zhaohong Chen^20^, Xingang Wang^21^, Xiaojian Li^22^, Gaoxing Luo^23^, Yi Zhang^24^, Hongyan Zhang^25^, Shichu Xiao^1,2^, Liang Xiao^26,27^, Ruijin Guo^26,27^, Jie Zhang^28^, Xiaofei Ye^29,^ Hongtai Tang^1,2^, Qiao Zuo^30^, Bin Qu^31^, Bingwei Sun^32^, Changhong Lu^33^, Chengliang Deng^34^, Deyi Zheng^35^, Geng Ji^36^, Haisheng Li^23^, Haizhou Yu^37^, Hua Zhou^38^, Jian Wu^39^, Lanjun Nie^40^, Lei Wang^40^, Pihong Zhang^41^, Qingfu Zhang^42^, Shengwu Chao^43^, Sisi Yang^44^, Xianglin Ding^45^, Xiangsheng Ding^46^, Ying Yan^47^, Jiangyong Shen^48^, Yongjian Zhao^49^, Yunchuan Pan^50^, Yunbiao Shen^51^, Yu Zhang^52^, Zhihui Yao^53^, Zhe Cheng^54^, Jiangfan Xie^3^, Zhigang Chu^55^, Shijie Li^56^, Long Xu^1,2^, Xiuming Yang^1,57^, Sujie Xie^1,2^, Hanlin Sun^1,2^, Shuyuan Xian^1,2^, Dezhi Han^56*^, Maolong Dong^55*^, Chengde Xia^3*^, Peng Luo^58*^, Yongming Yao^59*^, Zhaofan Xia ^1,2*^, Shizhao Ji^1,2*^

^1^Department of Burn Surgery, The First Affiliated Hospital of Naval Medical University, Shanghai 200433, China

^2^Research Unit of Key Techniques for Treatment of Burns and Combined Burns and Trauma Injury, Chinese Academy of Medical Sciences, Shanghai 200433, China

^3^Department of Burn, The First People's Hospital of Zhengzhou, Zhengzhou 450004, China

^4^Department of Burns, The First Affiliated Hospital of Anhui Medical University, Hefei 230022, China

^5^Department of Burn and Plastic Surgery, Northern Jiangsu People's Hospital, Yangzhou, 225001, China

^6^Department of Burns and Plastic Surgery, The 910th Hospital of Joint Logistics Support Force of Chinese People's Liberation Army, Quanzhou 362000, China

^7^Department of Burns and Plastic Surgery, Huai'an First People's Hospital, Nanjing Medical University, Huai'an 223300, China

^8^Department of Burn and Plastic Surgery, Bethune International Peace Hospital, Shijiazhuang 050000, China

^9^Department of Burns and Plastic Surgery, The 924th Hospital of Joint Logistics Support Force of Chinese People's Liberation Army, Guilin 541002, China

^10^Department of Burns, The Fifth Hospital of Baoding City, Baoding 071000, China.

^11^Department of Burns, Beijing Jishuitan Hospital, Beijing 100035, China

^12^Department of Burn and Plastic Surgery, Marine Corps Hospital, Chaozhou 521000, China

^13^Burn Department, General Hospital of TISCO, Taiyuan 030009, China

^14^Department of Burn and Injury, The Second Affiliated Hospital of Kunming Medical University, Kunming 650033, China

^15^Department of Burns and Plastic Surgery, The First People's Hospital of Xuzhou, Xuzhou 221005, China

^16^Department of Burns Surgery, The Fifth Hospital of Harbin, Harbin 150040, China

^17^Department of Burn and Plastic Surgery, General Hospital of Northern Theater Command, Shenyang 110016, China

^18^Department of Burns and Plastic Surgery, The 909th Hospital of Joint Logistics Support Force of Chinese People's Liberation Army, Zhangzhou 363000, China

^19^Department of Burn, The 906th Hospital of Joint Logistics Support Force of Chinese People's Liberation Army, Wenzhou 325000, China

^20^Burn and Wound Repair Department, Fujian Medical University Union Hospital, Fuzhou 350001, China

^21^Department of Burns & Wound Care Center, The Second Affiliated Hospital, Zhejiang University School of Medicine, Hangzhou 310009, China

^22^Department of Burns and Plastic Surgery, Guangzhou Red Cross Hospital, Jinan University, Guangzhou 510220, China

^23^Institute of Burn Research, State Key Laboratory of Trauma and Chemical Poisoning, Southwest Hospital, Third Military Medical University, Chongqing 400038, China

^24^Department of Burn and Plastic Surgery, Affiliated Hospital of Nantong University, Nantong 226001, China

^25^Medical Center of Burn Plastic and Wound Repair, The First Affiliated Hospital, Jiangxi Medical College, Nanchang University, Nanchang 330031, China

^26^BGI Research, Wuhan, Hubei 430075, China

^27^State Key Laboratory of Genome and Multi-omics Technologies, BGI Research, Shenzhen 518083, China

^28^Tongji University School of Medicine, Tongji University, Shanghai 200092, China

^29^Department of Health Statistics, Naval Medical University, Shanghai 200433, China

^30^Neurovascular Center, Changhai Hospital, Naval Medical University, Shanghai 200433, China

^31^Department of Burn Surgery, Sichuan Academy of Medical Sciences & Sichuan Provincial People's Hospital, Chengdu 610072, China

^32^Department of Burns and Plastic Surgery, Suzhou Hospital Affiliated to Nanjing Medical University, Suzhou 215002, China

^33^Department of Burns and Plastic Surgery, Staff Hospital of Handan Iron and Steel Group Company, Handan 056001, China

^34^Department of Burns and Plastic Surgery, The Affiliated Hospital of Zunyi Medical University, Zunyi 563003, China

^35^Department of Burn and Plastic Surgery, Guizhou Provincial People's Hospital, Guiyang 550002, China

^36^Department of Burn and Plastic Surgery, Taizhou People's Hospital, Taizhou 225300, China

^37^Department of Burn and Plastic Surgery, The Fourth Affiliated Hospital of Nantong University, Yancheng 224001, China

^38^Department of Burns, Ganzhou Municipal Hospital of Jiangxi Province, Ganzhou 341000, China

^39^Department of Burns and Plastic Surgery, Gansu Provincial People's Hospital, Lanzhou 730099, China

^40^Department of Burn and Plastic Surgery, The Second Affiliated Hospital of Nanjing Medical University, Nanjing 210011, China

^41^Department of Burns and Plastic Surgery, Xiangya Hospital of Central South University, Changsha 410008, China

^42^Burn and Wound Repair Center, The Third Hospital of Hebei Medical University, Shijiazhuang 050051, China

^43^Department of Burns and Plastic Surgery, The Affiliated Hospital of Qinghai University, Xining 810000, China

^44^Department of Burns and Plastic Surgery, The General Hospital of Chinese People's Liberation Army Tibet Military Area Command, Lhasa 850000, China

^45^Gansu Tianshui Xianglin Burn Specialty Hospital, Tianshui 741099, China

^46^Department of Burns, The Affiliated Lianyungang Hospital of Xuzhou Medical University, Lianyungang 222061, China

^47^Department of Burns and Plastic Surgery, General Hospital of Eastern Theater Command, Nanjing 210002, China

^48^Department of Burns and Plastic Surgery, General Hospital of Ningxia Medical University, Yinchuan 750004, China

^49^Department of Burn and Plastic Surgery, Tianjin Fourth Hospital, Nankai University, Tianjin 300322, China

^50^ Department of Burn & Skin Repair Surgery, Hainan General Hospital (Hainan Affiliated Hospital of Hainan Medical University), Haikou 570311, China

^51^Department of Burns and Plastic Surgery, Urumchi General Hospital of Lanzhou Command of Chinese People's Liberation Army, Urumchi 830000, China

^52^Department of Burn, The Second Hospital of the Southern Theater Command Navy, Sanya 572317, China

^53^Department of Burns and Plastic Surgery, The 926th Hospital of Joint Logistics Support Force of Chinese People's Liberation Army, Kaiyuan 661699, China

^54^Department of Burns and Plastic Surgery, The 928th Hospital of Joint Logistics Support Force of Chinese People's Liberation Army, Haikou 570311, China

^55^Department of Burns, Tongren Hospital of Wuhan University & Wuhan Third Hospital, Wuhan 430060, China

^56^Department of Burn and Plastic Surgery, The 969th Hospital of Joint Logistics Support Force of Chinese People's Liberation Army, Hohhot 010051, China

^57^Department of Burn and Plastic Surgery, The Third Affiliated Hospital of Naval Medical University, Shanghai 200433, China

^58^Department of Oncology, Zhujiang Hospital, Southern Medical University, Guangzhou 510282, China

^59^Translational Medicine Research Center, Medical Innovation Research Division and The Fourth Medical Center of Chinese People's Liberation Army General Hospital, Beijing 100853, China

^#^These authors contributed equally: Runzhi Huang, Yixu Li and Xiaoliang Li have contributed equally to this work.

*Correspondence: shizhaoji2022@163.com (Shizhao Ji); [xiazhaofan_smmu@163.com](mailto:xiazhaofan_smmu@163.com) (Zhaofan Xia); [c_ff@sina.com](mailto:c_ff@sina.com) (Yongming Yao); luopeng@smu.edu.cn (Peng Luo); xiachengde@tom.com (Chengde Xia); d252628@126.com (Maolong Dong); dezhi_han@126.com (Dezhi Han).

## **Clinical Study Protocol**

| Title: | BIOME Cohort: Multi-System Microbiome Dynamics and Clinical Outcomes in Massive Burn Injury Patients, a Multicenter, Longitudinal, and Prospective Study |
| --- | --- |
| Investigational Product(s): | Not Applicable |
| Protocol Number: | CHEC2025-071 |
| Version | Version 1.1 |
| Sponsor: | The First Affiliated Hospital of Naval Medical University |
| Collaborators: | Not Applicable |
| Principal Investigator: | Prof. Peng Luo, Prof. Yongming Yao, Prof. Zhaofan Xia, Prof. Shizhao Ji |
| Date: | 2025.12.13 |

Confidentiality

All information contained in this clinical trial protocol is the property of the sponsor and is provided in strict confidence to investigators, potential investigators, or consultants. Access is limited to these individuals, their designated staff, and the relevant independent ethics committee or institutional review board, in accordance with applicable regulatory requirements. No part of this protocol may be disclosed to third parties without the prior written authorization of the sponsor, unless such disclosure is required to obtain informed consent from participants for the use of the investigational product, in compliance with applicable laws and regulations.

## Statement of Principal Investigator

I have reviewed this clinical trial protocol and agree to its contents. I undertake to conduct the study strictly in accordance with the protocol. I will ensure that copies of this protocol and all relevant documents are distributed to all investigators participating in this trial. Furthermore, I will discuss the distributed documents with the investigators to ensure their full understanding of the investigational product and the procedures for conducting the trial.

**Principal Investigator:** Prof. Peng Luo, Prof. Yongming Yao, Prof. Zhaofan Xia, Prof. Shizhao Ji

## **Synopsis**

|  | **Product:** | | Not Applicable |
| --- | --- | --- | --- |
|  | **Title:** | | BIOME Cohort: Multi-System Microbiome Dynamics and Clinical Outcomes in Massive Burn Injury Patients, a Multicenter, Longitudinal, and Prospective Study |
|  | **Protocol Number:** | | CHEC2025-071 Version 1.1 |
|  | **Study Type:** | | A prospective cohort study initiated by the investigator |
|  | **Background and Principles:** | | Massive burn injuries (MBIs) are defined as burns involving > 50% of the total body surface area (TBSA) or third-degree burns affecting > 20% [1]. According to the World Health Organization (WHO), burns represent a major global health burden, resulting in millions of non-fatal injuries and over 300,000 deaths annually [2]. Burns profoundly disrupt the human microbiota, including skin [2,3], gut [4], and respiratory communities [5]. Dysbiosis within these ecosystems may result in severe infections such as sepsis and multiple organ dysfunction syndrome (MODS), thereby worsening treatment outcomes and prognosis [6,7]. Growing evidence indicates that skin microbiota dysbiosis contributes to the onset and progression of dermatological disorders, while microbiota reconstruction promotes wound healing by re-establishing the skin barrier [8,9]. The skin microbiome is also closely interconnected with gut, oral, and respiratory microbiota, together maintaining systemic homeostasis [10,11]. Emerged evidence has indicated that microbiomes become key targets for the monitoring and treatment of sepsis. However, most current studies remain limited to observational associations or animal models, typically focused on a single microbiota niche at one time point [12−14]. Large-scale, systematic clinical cohorts tracking the spatiotemporal dynamics of multiple microbial ecosystems in critically burned patients are lacking. This gap limits understanding of the mechanistic roles of microbiota in disease progression and hampers development of microbiota-targeted interventions [15−17]. Therefore, comprehensive clinical cohort studies in systemic injury models such as MBIs are urgently needed to construct dynamic atlases of multi-system microbiota and elucidate their coupling patterns and clinical relevance during recovery.  To address these critical gaps, we initiated the Burn & Trauma Injury Organisms and Microbiome Ecology (BIOME) cohort: a multicenter, longitudinal, and prospective study designed to investigate the dynamics of the microbiome system during the comprehensive treatment process in patients with MBI. We plan to collect skin swabs, gut samples, oral samples, environmental samples, and clinical information from at least 150 MBI patients with burn wounds >50% TBSA. We will systematically depict the skin microbial profiles of burn wounds at different time points and compare the dynamic variation in the skin microbiota with the commensal microbiota of other sites (oral, and intestinal) during the course of the MBI (strong stress state). |
|  | **Objective:** | | 1. Establish the first nationwide multicenter cohort of MBI patients, enabling long-term follow-up and comprehensive microbiome profiling. 2. Investigate alterations and interrelationships between skin microbiota and microbial communities at other body sites (e.g., gut, oral cavity) under systemic stress induced by MBI. 3. Characterize temporal dynamics of skin microbiota during distinct phases of wound healing and scar formation after MBI. 4. Develop predictive models linking microbial signatures to clinical trajectories, aiming to identify key microbial determinants of infection, complication risk, wound healing, and scarring. 5. Elucidate the diagnostic and therapeutic potential of microbiota-derived biomarkers in burn care, paving the way for precision medicine in wound management. |
|  | **Study Design:** | | A Multicenter, Longitudinal, Prospective Observational Study |
| **Sample Size:** | | At least 150 patients will be enrolled in total. | |
| **Centre:** | | Multicenter center in China | |
| **Duration of Study (Year):**  **From the day when the first participant is admitted to the last participant completes his/her trial and the interim analysis will be implemented during this period.** | | July 1, 2025 to June 30, 2028 | |
| **Inclusion Criteria:** | | 1. Age 18–65 years; 2. MBI patients with >50% TBSA who were admitted to the hospital within 72 hours of burn injury; 3. Patients or designated agents and guardians agreed to participate in this study and signed an informed consent form. | |
| **Exclusion Criteria:** | | 1. Patients with underlying endocrine disorders (e.g., diabetes mellitus, hyperthyroidism); 2. Patients diagnosed with hematologic diseases or malignancies of the skin or internal organs; 3. Patients with autoimmune diseases, immunodeficiency disorders, or immunosuppression (e.g., patients undergoing high-dose immunosuppressive therapy); 4. Patients with pre-existing dermatologic conditions (e.g., psoriasis, vitiligo) in combination; 5. Pregnant or lactating women; 6. Any other conditions deemed by the investigators to potentially affect study outcomes. | |

| **Formulation(s):** | Biological sampling will be performed at standardized time points: 1-2 ± 1, 3 ± 1, 7 ± 1, 14 ± 1, and 28 ± 1 days post-burn; on the day of wound healing; and at 3, 6, and 12 months (± 7 days) post-burn. These time points were selected to correspond to the major clinical phases of MBI: shock (resuscitation), infection (hypermetabolic), reparative (proliferative), and rehabilitation (recovery). At each time point, comprehensive biospecimens and clinical data will be collected, including:   1. Skin swab samples from burned and unburned regions of the torso and radial side of the extremities, 2. Intestinal samples (fecal specimens or anal swabs), 3. Oral swab samples, 4. All clinical data including demographic information, injury characteristics, auxiliary examinations, laboratory tests, treatment details, outcomes/endpoints, and other relevant variables. |
| --- | --- |
| **Dosage and**  **Administration:** | To ensure consistency and minimize technical variability, all participating centers will use standardized nucleic acid preservation reagents, sample tubes, and sterile saline, which will be centrally distributed by The First Affiliated Hospital of Naval Medical University on a quarterly basis. Detailed standard operating procedures (SOPs) for sample acquisition and negative control handling are provided in the Supplementary Information. To further reduce both technical variability and systemic error, all centers will employ standardized biospecimen collection kits, also distributed quarterly by The First Affiliated Hospital of Naval Medical University. |
| **Treatment Course:** | Not Applicable |
| **Standard treatment:** | Patients with MBI require continuous, stage-specific treatment beginning immediately after injury and extending up to one year. Management generally follows the phases of shock, infection, wound repair, rehabilitation, and scar formation.  During the shock phase, the primary objectives are fluid resuscitation and stabilization of circulation. Treatment is guided by total burn surface area and depth, with fluid replacement monitored through central venous access and urine output. Airway protection and respiratory support are prioritized, and mechanical ventilation or tracheostomy is provided when necessary to maintain oxygenation and organ perfusion.  In the infection phase, the patient is highly susceptible to infection and sepsis. Standard therapy includes early debridement of necrotic tissue, appropriate antimicrobial administration, and wound coverage using biologic or silver-based dressings. Nutritional therapy is intensified with early enteral feeding, high-protein diets, and immune-enhancing nutrients to support host defense and reduce complications.  The wound repair phase focuses on epithelialization and wound closure. Conventional management involves staged excision and grafting, supplemented by allogeneic skin or dermal substitutes. Biologic dressings and tissue-engineered scaffolds may be used to promote wound coverage, minimize infection, and improve long-term outcomes.  In the rehabilitation phase, the emphasis shifts toward functional recovery. Interventions include structured physical therapy, joint mobility exercises, and psychosocial support. Scar prevention measures such as compression garments, pressure therapy, and pharmacologic adjuvants are incorporated to prevent contractures and dysfunction.  The scar formation phase requires long-term follow-up and targeted scar modulation. Standard strategies include pressure therapy, silicone gel applications, laser treatment, and, when necessary, surgical revision to optimize cosmetic and functional recovery.  All treatments are administered in accordance with established national and international guidelines, such as those of the American Burn Association (ABA) and the Chinese Society of Burn Surgery [1].  This study is designed as a multicenter, prospective, longitudinal observational study. All biological sampling, including skin swabs, oral swabs, stool or anal swabs, will be conducted using noninvasive or minimally invasive procedures. Importantly, no additional interventions will be introduced that could alter diagnostic or therapeutic decision-making. Sampling will be performed strictly within the framework of routine care, ensuring that patient management follows the standard clinical pathway without interference. The study thus aims to provide a comprehensive depiction of treatment and recovery in MBI patients while maintaining complete clinical safety and integrity. |
| **Randomization:** | Not applicable. |
| **Endpoint and outcome:** | The primary outcomes of this study are defined as new-onset sepsis, and all-cause mortality. The diagnosis of sepsis will follow the criteria of the American Burn Association (ABA) [1]. The study duration is one year after burn injury. For patients who die during hospitalization, the exact date and time of death will be recorded. Study participation will end at one-year post-burn or upon patient death, whichever occurs first. |

| **Safety Indices:** | This study is designed for sample collection and does not interfere with clinical decision-making. Investigators are required to follow standard medical procedures and treatment protocols; monitor identify and manage adverse events. |
| --- | --- |
| **Primary Statistical Method:** | Metagenomic sequencing of multi-site and multi-timepoint samples: Samples obtained from different anatomical sites and multiple time points will be subjected to metagenomic sequencing. Data will be analyzed using Python- and R-based pipelines together with epidemiological statistical tools to explore: (i) the dynamic shifts of microbial ecology during skin barrier reconstruction in burned and unburned regions of patients with massive burns; and (ii) the temporal variation of commensal microbial communities at mucosal sites, including the respiratory and gastrointestinal tracts, throughout wound repair and tissue remodeling.  Epidemiological design and metagenomic GWAS analyses: Observational study frameworks and genome-wide association study (GWAS)-based bioinformatics methods will be applied to establish correlations between microbiome evolution and clinically significant events in patients with severe burns. Particular attention will be directed toward the associations among wound healing, scar formation, and the overall evolution of microecological systems. Statistical analyses will further evaluate interactions between microbial dynamics and host clinical trajectories.  Bioinformatics and experimental validation: Integrative bioinformatics combined with experimental validation will be used to elucidate the mechanisms underlying host–microbe interactions during wound repair and scar formation. Key microbial taxa and functional pathways involved in regulating wound healing outcomes will be identified, and their interactions with host responses will be characterized to define their mechanistic contributions to recovery. |
| **Interim Analysis:** | Not applicable. |
| **First Aids:** | Not applicable. |

## Abbreviations

| **Abbreviation** | **Full** |
| --- | --- |
| ABA | American Burn Association |
| ACL | Access Control List |
| ACME | Average Causal Mediation Effect |
| ADL | Activities of Daily Living |
| ADLB | Laboratory Dataset |
| ADMG | Metagenomic Dataset |
| ADSL | Subject-Level Dataset |
| AE | Adverse Event |
| AI | Artificial Intelligence |
| ANOSIM | Analysis of Similarities |
| AS | Anal Swab |
| AUC | Area Under Curve |
| BIOME | Burn & Trauma Injury Organisms and Microbiome Ecology |
| BP | Bodily Pain |
| BW | Burn Wounds |
| CARD | Comprehensive Antibiotic Resistance Database |
| CDISC | Clinical Data Interchange Standards Consortium |
| CLR Transformation | Centered Log-Ratio Transformation |
| Co-I | Co-Investigator |
| CRF | Case Report Form |
| CTCAE | Common Terminology Criteria for Adverse Events |
| DAC | Data Access Committee |
| DCA | Decision Curve Analysis |
| DID model | Difference-in-Differences Model |
| DUA | Data Use Agreement |
| dL | Deciliter |
| DSMB | Data and Safety Monitoring Board |
| DVT | Deep Venous Thrombosis |
| eCRF | Electronic Case Report Form |
| EDC | Electronic Data Capture |
| EDTA | Ethylenediaminetetraacetic Acid |
| FDR | False Discovery Rate |
| FiO2 | Fraction of Inspired Oxygen |
| FOBT | Fecal Occult Blood Test |
| GCP | Good Clinical Practice |
| GH | General Health |
| GMM | Gaussian Mixture Model |
| GSEA | Gene Set Enrichment Analysis |
| HR | Hazard Ratio |
| HT | Health Transition |
| ICC | Intraclass Correlation Coefficient |
| ICD | International Classification of Diseases |
| ICF | Informed Consent Form |
| ICH | International Council for Harmonisation |
| ICU | Intensive Care Unit |
| ID | Identity Document |
| IEC | Institutional Ethics Committee |
| IPTW | Inverse Probability of Treatment Weighting |
| IRB | Institutional Review Board |
| KEGG | Kyoto Encyclopedia of Genes and Genomes |
| kg | Kilogram |
| kPa | Kilopascal |
| L | Liter |
| LASSO | Least Absolute Shrinkage and Selection Operator |
| LCA | Lowest Common Ancestor |
| LDA | Linear Discriminant Analysis |
| LEfSe | Linear Discriminant Analysis Effect Size |
| LIME | Local Interpretable Model-agnostic Explanations |
| LIS | Laboratory Information System |
| LMM | Linear Mixed Model |
| MAP | Mean Arterial Pressure |
| MBI | Massive Burn Injury |
| MDIS | Medical Digital Identity System |
| mg | Milligram |
| MH | Mental Health |
| mHLA-DR | monocyte Human Leukocyte Antigen DR |
| min | Minute |
| MIxS | Minimum Information about any Sequence |
| mL | Milliliter |
| mm | Millimeter |
| mmHg | Millimeters of mercury |
| MODS | Multiple Organ Dysfunction Syndrome |
| MSM | Marginal Structural Model |
| NCI | National Cancer Institute |
| ng | Nanogram |
| NMDS | Non-Metric Multi-Dimensional Scalin |
| NRI | Net Reclassification Index |
| NS | Normal Skin |
| OS | Oral Swab |
| PaO2 | Partial pressure of arterial oxygen |
| PCA | Principal Component Analysis |
| PCoA | Principal Coordinates Analysis |
| PDP | Partial Dependence Plot |
| PF | Physical Functioning |
| PGP | Pretty Good Privacy |
| PI | Principal Investigator |
| PPE | Personal Protective Equipment |
| QC | Quality Control |
| qPCR | Quantitative real-time PCR |
| qSOFA | Quick Sequential Organ Failure Assessment |
| RE | Role-Emotional |
| ROC | Receiver Operating Characteristic Curve |
| RP | Role-Physical |
| RR | Relative Risk |
| SAE | Serious Adverse Event |
| SAP | Statistical Analysis Plan |
| SDV | Source Data Verification |
| SEM | Structural Equation Modeling |
| SF | Social Functioning |
| SF-36 | 36-item Short-Form |
| SFTP | SSH File Transfer Protocol |
| SHAP | SHapley Additive exPlanations |
| SMD | Standardized Mean Difference |
| SOFA | Sequential Organ Failure Assessment |
| SOP | Standard Operating Procedure |
| SS | Stool Sample |
| STAMP | Spatial Transcriptomic-Microbial Association |
| Sub-I | Sub-Investigator |
| TBSA | Total Body Surface Area |
| TRIPOD | Transparent Reporting of a multivariable prediction model for Individual Prognosis or Diagnosis |
| ug | Microgram |
| ul | Microliter |
| umol | Micromole |
| UPGMA | Unweighted Pair-group Method with Arithmetic Mean |
| VSS | Vancouver Scar Scale |
| VT | Vitality |
| WHO | World Health Organization |
| XGBoost | eXtreme Gradient Boosting |

# **Introduction**

The human microbiome represents a dynamic and integrated ecosystem encompassing host–microbe interactions at structural, functional, and molecular levels. This intricate network contributes to host metabolism, immune regulation, barrier integrity, and disease progression across the lifespan [19−21]. The skin, as the largest organ and primary physical barrier of the human body, harbors highly structured microbial communities that function as an ecological shield, orchestrating immune homeostasis and protecting against pathogen invasion [22,23]. Perturbations of the skin microbiome have been implicated in a wide spectrum of inflammatory and infectious dermatoses, while restoration of microbial balance has been shown to facilitate cutaneous wound healing and re-establishment of the skin barrier [24,25]. Moreover, the skin microbiome is interconnected with other ecological niches such as the gut, respiratory tract, and oral cavity [15,17], forming a system-level network that collectively maintains host homeostasis. Despite increasing recognition of these interactions, most current evidence is limited to associative studies focusing on a single niche or preclinical models, with a paucity of systematic clinical investigations capturing multi-organ, multi-timepoint microbiome dynamics during disease states [20,21].

Massive burn injury provides a unique and extreme model of systemic immune dysregulation and barrier disruption. Beyond extensive destruction of the skin’s physical shield, burn wounds create nutrient-rich environments favorable for opportunistic microbial colonization, thereby predisposing patients to invasive infections and sepsis [14,26,27]. Epidemiological data indicate a bimodal distribution of burn-related mortality: an early peak within the first week due to shock and trauma, and a later peak at 4 – 6 weeks, primarily attributable to severe infections and sepsis [26−28]. Advances in resuscitation have reduced early mortality, yet infection-driven late deaths remain a major challenge, with endogenous microbial instability identified as a central driver [6,29]. Importantly, while prompt wound closure and grafting are standard strategies to restore physical barriers, they cannot fully reconstitute microbial homeostasis, leaving patients vulnerable to dysbiosis-associated systemic inflammation and infection [30−32].

Existing studies of burn-associated microbiome alterations have highlighted a decline in microbial richness and diversity at wound sites, with enrichment of opportunistic pathogens such as Pseudomonas aeruginosa, Enterobacteriaceae, and Acinetobacter species, and concomitant depletion of commensals such as Cutibacterium acnes and Staphylococcus epidermidis [3,22,33,334]. Recruitment of neutrophils and macrophages into the wound microenvironment further alters oxygen availability, pH, and nutrient composition, thereby reshaping microbial ecology [30−32]. However, these studies have been constrained by low resolution sequencing approaches, limited sampling timepoints, and insufficient integration with host immune or clinical outcomes, restricting our understanding of causal mechanisms and translational potential.

Against this backdrop, critical questions remain unresolved: How do multiple body-site microbiomes (skin, gut, respiratory tract, oral cavity, blood) dynamically evolve following massive burn injury (MBI)? What are the temporal and spatial coupling mechanisms linking physical barrier reconstruction with microbial barrier reconstitution? And to what extent do these microbiome dynamics influence infection susceptibility, immune reprogramming, and patient outcomes? Addressing these questions requires systematic, prospective, multicenter studies with high-resolution multi-omics approaches to construct a comprehensive spatiotemporal map of burn-associated microbiome dynamics.

The BIOME project is designed to fill this critical knowledge gap. By establishing a prospective, multicenter cohort of patients with extensive burns, this study aims to delineate the longitudinal evolution of microbiomes across multiple anatomical niches, characterize the interplay between skin barrier healing and microbial barrier reconstruction, and identify predictive microbial signatures associated with infection risk and clinical outcomes. Elucidating these mechanisms holds major clinical significance: it will provide a systems-level understanding of infection pathogenesis in burn patients, inform strategies for microbiome-targeted interventions, and ultimately guide integrated approaches to barrier restoration, infection control, and immunomodulation in critical illness.

# **Objectives**

We initiated the Burn & Trauma Injury Organisms and Microbiome Ecology (BIOME) cohort: a multicenter, longitudinal, and prospective study designed to investigate the dynamics of the microbiome system during the comprehensive treatment process in patients with MBI. We plan to collect skin swabs, gut samples, oral samples, environmental samples, and clinical information from at least 150 MBI patients with burn wounds > 50% of the total body surface area (TBSA). We will systematically depict the skin microbial profiles of burn wounds at different time points and compare the dynamic variation in the skin microbiota with the commensal microbiota of other sites (oral, and intestinal) during the course of the MBI (strong stress state). Specifically, this study aims to:

- Establish the first nationwide multicenter cohort of MBI patients, enabling long-term follow-up and comprehensive microbiome profiling.
- Investigate alterations and interrelationships between skin microbiota and microbial communities at other body sites (e.g., gut, oral cavity) under systemic stress induced by MBI.
- Characterize temporal dynamics of skin microbiota during distinct phases of wound healing and scar formation after MBI.
- Develop predictive models linking microbial signatures to clinical trajectories, aiming to identify key microbial determinants of infection, complication risk, wound healing, and scarring.
- Elucidate the diagnostic and therapeutic potential of microbiota-derived biomarkers in burn care, paving the way for precision medicine in wound management.

# **Study Design**

A multicenter, longitudinal, prospective observational study.

# **Outcomes**

### **Primary Outcomes**

The primary outcomes of this study are defined as:

- New-onset sepsis (according to the criteria of the American Burn Association, ABA).
- All-cause mortality.

Study duration is one year after burn injury. For patients who die during hospitalization, the exact date and time of death will be recorded. Study participation will end at one year post-burn or upon patient death, whichever occurs first.

### **Secondary Outcomes**

Secondary outcomes include:

- Sepsis-related mortality.
- Incidence of new infections within one year.
- ICU mortality and hospital mortality beyond sepsis-related mortality.
- Length of ICU stays and hospital stays.
- Re-hospitalization rate within one year.
- Wound-healing time at sampling sites.
- Wound complications (e.g., delayed healing, graft loss, wound infection).
- Vancouver Scar Scale (VSS) score for scar assessment.
- Functional recovery indicators (such as mobility scores and range of motion in burn-injured areas).
- Quality of life assessments at 3, 6, and 12 months using validated scales (e.g., 36-item Short-Form (SF-36)).

### **Exploratory Outcomes**

Exploratory outcomes are designed to explore the systemic and local microbiome dynamics and their correlation with clinical events, including:

- Variations in skin, intestinal, and oral microbiome composition and diversity across different time points.
- Correlation between microbiome signatures and wound-healing trajectories.
- Identification of microbial or metabolic biomarkers predictive of sepsis, delayed healing, or scar formation.
- Integration of multi-omics data (metagenomics, metabolomics, transcriptomics) with clinical outcomes to identify mechanistic pathways.

These exploratory outcomes were not part of the formal hypothesis testing but aimed to generate insights for future studies and improve clinical decision-making in wound reconstruction strategies.

### **Safety Outcomes**

As this study is a prospective observational cohort, it does not involve interventional procedures or alterations to routine clinical care. Therefore, no direct safety outcomes are expected. However, adverse clinical events, serious adverse events, and complications will be recorded as part of standard clinical data collection to ensure comprehensive reporting. This reflects the real-world safety profile of patients with massive burn injuries without influencing clinical decision-making or patient autonomy.

# **Participants**

### **Sample Size Estimation**

At least 150 patients are planned to be enrolled in this study.

The BIOME cohort plan to enroll at least 150 patients with MBIs for longitudinal biospecimen collection and clinical follow-up. The planned sample size was justified using both event-rate–based and events-per-variable (EPV) considerations for the two primary outcomes (all-cause mortality and new-onset sepsis). A large disaster-related MBI cohort from the 2014 Kunshan explosion reported an all-cause mortality of 44.8% and a sepsis incidence of 68.5% in patients with TBSA > 50% [2].

For event-rate–based considerations, Assuming a two-sided significance level of α = 0.05 and a statistical power of:

1 – β = 0.80 (80%)

The minimum required sample size to detect a clinically meaningful difference in mortality and sepsis between microbiome-defined subgroups was calculated to be 82 and 76 patients, respectively.

To ensure adequate statistical power for multivariable modeling, we applied the Events Per Variable (EPV=10) principle to both primary outcomes. Based on the representative mortality rate of 44.8% and sepsis incidence of 68.5% from the Kunshan cohort, enrolling 150 patients is expected to yield:

150 × 44.8% ≈ 67 deaths

150 × 68.5% ≈ 103 sepsis events

These event counts allow inclusion of 6-7 variables for mortality models and 10-11 variables for sepsis models, providing sufficient power to evaluate microbial predictors while adjusting for major clinical confounders. Taken together, these four calculations (rate-comparison and EPV-based justifications for both mortality and sepsis) indicate that the planned sample size of 150 MBI patients provides sufficient statistical power for the primary analyses and for building clinically meaningful multivariable models.

### **Inclusion Criteria**

- Age 18–65 years;
- MBI patients with > 50% TBSA who were admitted to the hospital within 72 hours of burn injury;
- Patients or designated agents and guardians agreed to participate in this study and signed an informed consent form.

### **Exclusion criteria**

- Patients with underlying endocrine disorders (e.g., diabetes mellitus, hyperthyroidism);
- Patients diagnosed with hematologic diseases or malignancies of the skin or internal organs;
- Patients with autoimmune diseases, immunodeficiency disorders, or immunosuppression (e.g., patients undergoing high-dose immunosuppressive therapy);
- Patients with pre-existing dermatologic conditions (e.g., psoriasis, vitiligo) in combination;
- Pregnant or lactating women;
- Any other conditions deemed by the investigators to potentially affect study outcomes.

### **Participants Withdraw**

- Voluntary withdrawal: Participants (or their legal guardians) request to withdraw from the study at any time for personal reasons.
- Loss to follow-up: Failure to attend scheduled visits or inability to contact participants despite reasonable efforts.
- Protocol non-compliance: Significant deviation from study protocol or procedures that may affect the reliability of data.
- Clinical necessity: Requirement for additional medical or surgical intervention, as determined by the treating physician, that precludes continued study participation.
- Safety concerns: Occurrence of adverse events or intercurrent illnesses that, in the judgment of the investigator, may endanger participant safety if participation continues.
- Data integrity compromise: Receipt of concomitant treatments or procedures that could significantly interfere with outcome assessments.
- Investigator decision: Withdrawal deemed necessary by the Investigator or Lead Statistician for scientific, ethical, or safety considerations, with documented rationale.

# **Treatment**

### **Plan**

Biological sampling will be performed at standardized time points that correspond to the major clinical phases of burn injury:

- Shock/resuscitation phase: Day 1–2 (± 1) post-burn
- Infection/hypermetabolic phase: Day 3 (± 1), Day 7 (± 1), Day 14 (± 1), and Day 28 (± 1)
- Reparative/proliferative phase: at the day of wound healing
- Rehabilitation/recovery phase: 3 months, 6 months, and 12 months post-burn (each ± 7 days)

At each predefined time point, comprehensive biospecimens and clinical data will be systematically collected, including:

- Skin swab samples from both burned and unburned regions (torso and radial side of extremities);
- Intestinal samples (fecal specimens or anal swabs);
- Oral swab samples;
- Clinical data: demographic information, burn injury characteristics, treatment details, laboratory and imaging results, outcomes/endpoints, and other relevant variables.

To ensure consistency and minimize inter-center variability, all participating centers will employ standardized biospecimen collection kits, nucleic acid preservation reagents, and sterile saline, which will be centrally distributed on a quarterly basis by The First Affiliated Hospital of Naval Medical University. Sample acquisition will follow uniform Standard Operating Procedures (SOPs), including the handling of negative controls, as provided in the Supplementary Information.

Sample collection will be terminated upon ICU discharge or patient transfer, in order to align with ethical standards and clinical feasibility. To further ensure rigor, all samples will be stored using standardized procedures, and comprehensive documentation (protocol, SOPs, and statistical analysis plan (SAP)) will guide the entire process.

In summary, the BIOME protocol emphasizes rigorous and harmonized sampling across centers, while ensuring that study procedures remain strictly observational. All patient care and treatment decisions will be exclusively determined by attending clinicians, independent of study participation.

### **Labeling and Packaging**

Not applicable.

### **Sample labels:**

Not applicable.

### **Investigational Product Count**

Not applicable.

### **Grouping of Participants**

Not applicable.

### **Treatment or Dosage Selection**

Not applicable.

### **Blinding Method**

Not applicable.

### **Concomitant Therapy**

Not applicable.

### **Standard Treatment**

Patients with massive burn injuries (MBI) require continuous, stage-specific treatment beginning immediately after injury and extending up to one year. Management generally follows the phases of shock, infection, wound repair, rehabilitation, and scar formation.

During the shock phase, the primary objectives are fluid resuscitation and stabilization of circulation. Treatment is guided by total burn surface area and depth, with fluid replacement monitored through central venous access and urine output. Airway protection and respiratory support are prioritized, and mechanical ventilation or tracheostomy is provided when necessary to maintain oxygenation and organ perfusion.

In the infection phase, the patient is highly susceptible to infection and sepsis. Standard therapy includes early debridement of necrotic tissue, appropriate antimicrobial administration, and wound coverage using biologic or silver-based dressings. Nutritional therapy is intensified with early enteral feeding, high-protein diets, and immune-enhancing nutrients to support host defense and reduce complications.

The wound repair phase focuses on epithelialization and wound closure. Conventional management involves staged excision and grafting, supplemented by allogeneic skin or dermal substitutes. Biologic dressings and tissue-engineered scaffolds may be used to promote wound coverage, minimize infection, and improve long-term outcomes.

In the rehabilitation phase, the emphasis shifts toward functional recovery. Interventions include structured physical therapy, joint mobility exercises, and psychosocial support. Scar prevention measures such as compression garments, pressure therapy, and pharmacologic adjuvants are incorporated to prevent contractures and dysfunction.

The scar formation phase requires long-term follow-up and targeted scar modulation. Standard strategies include pressure therapy, silicone gel applications, laser treatment, and, when necessary, surgical revision to optimize cosmetic and functional recovery.

All treatments are administered in accordance with established national and international guidelines, such as those of the American Burn Association (ABA) and the Chinese Society of Burn Surgery [1].

This study is designed as a multicenter, prospective, longitudinal observational study. All biological sampling, including skin swabs, oral swabs, stool or anal swabs, will be conducted using noninvasive or minimally invasive procedures. Importantly, no additional interventions will be introduced that could alter diagnostic or therapeutic decision-making. Sampling will be performed strictly within the framework of routine care, ensuring that patient management follows the standard clinical pathway without interference. The study thus aims to provide a comprehensive depiction of treatment and recovery in MBI patients while maintaining complete clinical safety and integrity.

# **Evaluation**

### **Efficacy Evaluation**

Efficacy will be evaluated based on clinical outcomes related to wound repair, scar formation, and infection control in patients with massive burn injuries (MBI). The following parameters will be systematically assessed:

- Wound healing time at predefined sampling sites within 12 months post-burn.
- Wound healing rate within 28 days post-burn.
- Scar formation and severity, assessed using the Vancouver Scar Scale (VSS).
- Incidence of sepsis, defined according to the criteria of the American Burn Association (ABA).
- Mortality outcomes, including sepsis-related mortality and all-cause mortality.
- Complication rates, such as delayed healing, wound infection, or graft failure.
- Functional recovery and quality of life, assessed by validated instruments (e.g., SF-36) at 3, 6, and 12 months.

### **Safety Evaluation**

This study is designed as a multicenter, prospective, observational cohort. All participating patients will continue to receive standard clinical care as determined by their treating physicians, without any study-driven interventions or treatment modifications. Consequently, no study-related safety risks are anticipated.

Nevertheless, for comprehensive documentation, the following will be recorded from routine clinical practice:

- Adverse events (AEs) and serious adverse events (SAEs), including sepsis, complications of wound healing, and mortality.
- Clinical vital signs and laboratory test results, monitored as part of routine care, will be collected into the electronic case report form (eCRF).
- Any abnormal findings will be interpreted by investigators according to clinical practice, but attribution to the study will not apply.

The assessment of AEs, including abnormalities in laboratory parameters, will be graded according to the Common Terminology Criteria for Adverse Events (CTCAE), version 4.03, issued by the U.S. National Cancer Institute (NCI). The severity grades are defined as follows:

- Grade 1 (Mild) indicates asymptomatic or mild symptoms with only clinical or diagnostic observations and no intervention needed.
- Grade 2 (Moderate) involves minimal or local noninvasive intervention and may limit instrumental activities of daily living (ADL) such as meal preparation, shopping, telephone use, or money management.
- Grade 3 (Severe) represents severe or medically significant events that are not immediately life-threatening but are disabling. These events limit self-care ADL—such as bathing, dressing, feeding, using the toilet, or taking medications—and typically require hospitalization or prolonged hospital stay.
- Grade 4 (Life-threatening) refers to events with life-threatening consequences requiring urgent intervention.
- Grade 5 (Death) indicates a fatal outcome related to the adverse event.

Monitoring of vital signs including body temperature, systolic and diastolic blood pressure, heart rate, and respiratory rate will be conducted at baseline and on study procedures. All physiological measurements will be systematically recorded in the electronic case report form (eCRF) at each scheduled clinical visit.

In summary, the safety evaluation is descriptive and non-interventional, reflecting the real-world clinical profile of MBI patients. The BIOME study will not alter clinical decision-making or patient autonomy.

# **Study Procedures**

### **Screening**

The following observations and procedures will be completed prior to enrollment:

- Checking participant against inclusion/exclusion criteria
- Obtaining of an EC-approved ICF signed
- Obtaining of the medical history

### **IWRS Randomization**

Not applicable.

### **Procedures: Baseline and Day 1–2 (± 1) Post-Burn**

Biospecimen collection (6 types):

- Skin swab from burned torso region.
- Skin swab from unburned torso region.
- Skin swab from burned radial side of extremities.
- Skin swab from unburned radial side of extremities.
- Intestinal specimen (feces or anal swab).
- Oral swab.

Clinical data entry into standardized information collection system: At enrollment, a new patient record will be created in the standardized electronic data collection system (EDC). Data collection must strictly follow the SOPs, which specifies variable formats (e.g., continuous variable units, categorical variable levels) and upload standards (e.g., the images of sampling sites be uploaded as photos). The clinical dataset includes five domains:

Personal Information (baseline only)

- Past health status
- Gender
- Age
- Height
- Weight

Burn Injury Information (baseline and major clinical milestones)

- Cause of burn injury (e.g., flame, scald, chemical, electrical)
- Total body surface area (TBSA) affected
- Anatomical locations of burn injury
- Presence of concomitant injuries
- Photographs documenting overall injury status at admission

Treatment Information (updated at relevant timepoints)

- Pre-hospital emergency care (e.g., airway management, fluid resuscitation)
- Resuscitation details (e.g., shock fluid volume and type)
- Wound management (initial debridement, dressing)
- Infection prevention measures (antibiotics, antiseptic dressings)
- Nutritional support interventions
- Complication prevention strategies (e.g., deep venous thrombosis (DVT) prophylaxis, organ support)

Other Information (collected as available)

- Treating institution (hospital name/center code)
- Administrative division (geographical/urban–rural category)
- Socioeconomic data (if available, e.g., insurance type, income bracket)

Prognosis and Follow-up Information (longitudinal; collected at specific endpoints)

- Sepsis occurrence (per ABA definition)
- Sepsis-related mortality
- All-cause mortality
- Wound-healing time (days to complete closure at sampling sites)
- Vancouver Scar Scale (VSS) total and subscores (pigmentation, vascularity, pliability, height, pain, pruritus; only after wound healing is achieved)

### **Procedures: Day 3 (± 1) Post-Burn**

Biospecimen collection (2 types): Intestinal specimen, oral swab.

Clinical data collection: Routine update.

### **Procedures: Day 7 (± 1) Post-Burn**

Biospecimen collection (2 types): Intestinal specimen, oral swab.

Clinical data collection: Routine update.

### **Procedures: Day 14 (± 1) Post-Burn**

Biospecimen collection (2 types): Intestinal specimen, oral swab.

Clinical data collection: Routine update.

### **Procedures: Day 28 (± 1) Post-Burn**

Biospecimen collection (2 types): Intestinal specimen, oral swab.

Clinical data collection: Routine update.

### **Procedures: Sepsis Onset Day (for 3 consecutive days)**

Biospecimen collection (2 types): Intestinal specimen, oral swab.

Clinical data collection: Routine update. Sepsis-related data, laboratory findings, Imagological examination.

### **Procedures: Wound-Healing Day**

Biospecimen collection (6 types): Same as baseline (burned/unburned torso and extremity skin swabs, intestinal specimen, oral swab).

Clinical data collection: Routine update. Wound healing time/rate and scar assessment.

### **Procedures: Month 3 (± 7 days) Post-Burn**

Biospecimen collection (6 types): Same as baseline (burned/unburned torso and extremity skin swabs, intestinal specimen, oral swab).

Clinical data collection: Routine update. Updated outcome data including VSS scoring, complications, and quality of life assessments.

### **Procedures: Month 6 (± 7 days) Post-Burn**

Biospecimen collection (6 types): Same as baseline (burned/unburned torso and extremity skin swabs, intestinal specimen, oral swab).

Clinical data collection: Routine update. Updated outcome data including VSS scoring, complications, and quality of life assessments.

### **Procedures: Month 12 (± 7 days) Post-Burn**

Biospecimen collection (6 types): Same as baseline (burned/unburned torso and extremity skin swabs, intestinal specimen, oral swab).

Clinical data collection: Routine update. Updated outcome data including VSS scoring, complications, and quality of life assessments.

Study termination: Participation ends at 12 months post-burn or at time of death (if earlier). Date and time of death will be documented for deceased patients. This study is observational and will not interfere with any clinical decision-making, patient management, or treatment pathways. All sample collections and clinical data acquisitions will be conducted alongside routine medical care, following unified SOPs across all participating centers.

# **Study Preparation and Overall Management SOPs**

### **SOP01: Training and Authorization Management of Study Personnel**

Scope: Principal Investigator (PI), Sub-Investigator (Sub-I), Co-Investigator (Co-I), sampling personnel, sample quality control (QC) personnel, central laboratory staff, and data analysis personnel across all clinical centers.

Purpose: The purpose of this SOP is to ensure that all study personnel are adequately trained in standardized procedures, thereby guaranteeing consistency of multicenter study data, integrity of sample quality, and compliance with ethical and regulatory standards.

Research Roles and Corresponding Training Modules:

PI, Sub-I, Co-I :

Core Responsibilities: Full study oversight, ethical compliance, emergency decision-making.

Mandatory Training Modules:

- Study protocol interpretation (inclusion/exclusion criteria, sample types, time windows).
- Sepsis emergency procedures.
- Privacy protection and data confidentiality regulations.

Sampling Personnel:

Core Responsibilities: Aseptic collection of skin, intestinal, and oral samples.

Mandatory Training Modules:

- Standardized sampling procedures (including negative controls C1–C5).
- Time-window control (± 24 h).
- Immediate cryopreservation procedures.

Sample QC Personnel:

Core Responsibilities: Verification of sample integrity, contamination prevention.

Mandatory Training Modules:

- Acceptance criteria (swab completeness, stool sample adequacy).
- Temperature monitoring and log verification.
- Contamination risk identification.

Central Laboratory Personnel:

Core Responsibilities: Sample storage, transport, nucleic acid extraction.

Mandatory Training Modules:

- Storage at -80℃.
- Dry ice transport standards (≤ -70℃).
- Batch effect control (standardized consumables).

Data Analysis Personnel:

Core Responsibilities: Metagenomic data processing and bioinformatics analysis.

Mandatory Training Modules:

- Data anonymization and coding rules.
- Sequencing QC criteria (Q30 ≥ 80%).
- Statistical analysis plan (SAP).

Authorization Process: All personnel must undergo the following process: theoretical training → practical simulation → authorization.

Authorization criteria:

- PI, Sub-I, Co-I: participation in protocol compliance review and ethical discussion.
- Sampling Personnel: practical examination ≥ 90%, simulated sampling contamination rate ≤ 3%.
- Sample QC Personnel: strict adherence to acceptance criteria (swab integrity, stool adequacy).
- Data QC Personnel: sequencing data quality control (Q30 ≥ 80%).
- Other personnel: module examination ≥ 90%.

Assessment Questions: A training exam consisting of 20 questions: 10 single-choice, 5 true/false, and 5 short-answer questions.

Single-choice Questions:

1. Which of the following is a mandatory criterion for subject enrollment? (C)

A. Age 18–70 years;

B. Admission within 5 days post-burn;

C. TBSA > 50% burn area;

D. Enrollment with verbal consent from family.

2. Which of the following is correct when collecting stool samples? (C)

A. Collect 1 g each time;

B. Sample can be exposed to air for 30 min;

C. Collect inner portion using sterile spoon;

D. Stool may be mixed with urine.

3. When should skin swab sampling be performed? (D)

A. After debridement;

B. After bathing;

C. 30 min after dressing change;

D. Before debridement or dressing change.

4. Which statement about sample freezing is incorrect? (C)

A. All samples must be transferred to -80℃ within 1 h;

B. Rapid freezing in liquid nitrogen before ultra-low freezer;

C. Temporary storage at -20℃ for 48 h is acceptable;

D. Store in pre-labeled cryotubes.

5. What is the correct order for oral swab sampling? (D)

A. Throat first, then tongue dorsum;

B. Oral cavity must be completely dry;

C. One swab per site only;

D. Throat sampling should be performed last.

6. Which of the following about sample labeling and recording is correct? (C)

A. Only record sampling time;

B. Source of sample need not be labeled;

C. Each sample must record time, site, and details;

D. Depth for anal swab need not be recorded.

7. What is the required depth for anal swab sampling? (C)

A. 1–2 cm;

B. 3–4 cm;

C. 4–5 cm;

D. 6–7 cm.

8. Which is the main responsibility of sample QC personnel? (B)

A. Nucleic acid extraction;

B. Verify swab integrity and stool adequacy;

C. Arrange investigator shifts;

D. Write analysis scripts.

9. Which responsibility belongs to central laboratory personnel? (C)

A. Manage ethical documents;

B. Check informed consent;

C. Control batch effects;

D. Decide sample types.

10. Which is the training focus for sampling personnel? (D)

A. Metagenomic sequencing platform use;

B. Emergency decision-making;

C. Cold-chain transport process;

D. Aseptic sampling and contamination control.

True/False Questions:

1. Sampling can be performed within 3 h after debridement without timing restrictions. (F)

2. Liquid nitrogen rapid freezing is a required pretreatment step for all samples. (T)

3. Swab samples may be kept at room temperature for 30 min before freezing. (F)

4. All personnel must pass training modules with ≥ 90% before authorization. (T)

5. The PI does not participate in training assessments and only handles ethical reports. (F)

Short-answer Questions

1. List three key measures to control sample contamination.

Reference Answer: Aseptic operation, double-check verification; Use negative controls (C1–C5); Immediate freezing in liquid nitrogen.

2. List two key steps in the preparation phase of anal swab sampling.

Reference Answer: Clean perianal area (soap + 70% ethanol); Sampling should ideally be performed after a 30-minute window of no local care to minimize contamination. However, immediate clinical interventions (e.g., salvage) take precedence. In such instances, sampling is performed after the necessary care is completed, and this deviation is documented in the case records.

3. Why should tonsillar areas be avoided during oral swab sampling?

Reference Answer: To reduce contamination by non-target microbes; To minimize patient discomfort and improve success rate.

4. How does a QC personnel determine if a swab sample is qualified?

Reference Answer: Swab intact without breakage; Tube sealed tightly without leakage; Swab head visibly collected sample without abnormal color or foreign material.

5. List two measures to control batch effects in multicenter sample processing.

Reference Answer: Use consumables from the same batch (swabs/cryotubes); Process samples within uniform timeframes and protocols.

### **SOP02: Screening and Enrollment Procedures for Study Subjects**

Scope: Principal Investigators (PIs), Sub-Investigators (Sub-Is), Co-Investigators (Co-Is), and Sampling Personnel at each clinical center.

Purpose: To ensure that only patients are screened and enrolled, with strict adherence to inclusion and exclusion criteria, thereby guaranteeing study homogeneity.

Screening Criteria:

Inclusion Criteria (as per Protocol CHEC2025-071):

- Age 18–65 years;
- Patients with massive burn injuries (MBI) involving > 50% TBSA admitted to hospital within 72 hours of burn injury;
- Patients or designated legal representatives/guardians provide written informed consent to participate.

Exclusion Criteria:

- Patients with underlying endocrine disorders (e.g., diabetes mellitus, hyperthyroidism);
- Patients with hematologic diseases or malignancies involving the skin or internal organs;
- Patients with autoimmune diseases, immunodeficiency disorders, or immunosuppression (e.g., high-dose immunosuppressive therapy);
- Patients with pre-existing dermatologic conditions (e.g., psoriasis, vitiligo);
- Pregnant or lactating women;
- Any other conditions deemed by the investigator as potentially affecting study outcomes.

Withdrawal/Elimination Criteria:

- Patients who were mistakenly enrolled but later found not meeting inclusion criteria or meeting exclusion criteria;
- Patients with critical missing clinical data or multiple failed sample quality checks, as determined by the Data and Safety Monitoring Board (DSMB) to compromise key study outcomes;
- Patients who cannot adhere to the study protocol or demonstrate poor compliance during the study.

Operational Workflow:

- Step 1: Preliminary Screening (within 72h of admission)
  Verify age, TBSA percentage, and admission time.
- Step 2: Exclusion Verification
  Check systematically for six categories of exclusion conditions by reviewing medical history and laboratory reports.
- Step 3: Informed Consent
  Conduct consent signing in a private setting, with explicit statement of the right to withdraw at any time.
- Step 4: 8.4	Procedures: Baseline and Day 1–2 (± 1) Post-Burn
- Step 5: Patient Enrollment Code Assignment
  Code format: Center ID – Serial Number. Example: BIOME-CHYY01 denotes the first patient enrolled at Changhai Hospital, BIOME study.

Documentation of Enrollment Procedure Quality:

- Screening Log: This log records key screening details for each potential participant, including the screening date, patient identification code, the result of the screening (eligible/ineligible), and, if applicable, the specific reason for exclusion. All screening log data is entered and stored in the electronic database.
- Enrollment Confirmation Checklist: For each enrolled participant, a confirmation checklist is completed. This form documents the assigned patient code, the participant's initials, the exact date and time of enrollment, and the signature of the responsible investigator. This physical or electronic checklist is retained by the Principal Investigator at each participating study center.
- Elimination Record Form: In the event a participant is withdrawn or eliminated from the study after enrollment, this form must be completed. It requires a detailed account of the reason for elimination and an assessment of the completeness of the data collected up to that point. The completed form is submitted to the Lead Center for review and archiving.

### **SOP03: Informed Consent Signing and Management Procedures**

Scope: Principal Investigators (PIs), Sub-Investigators (Sub-Is), Co-Investigators (Co-Is), and sampling personnel at each clinical center.

Purpose: This SOP is established to standardize the process of informed consent signing, safeguard participant autonomy, and ensure compliance with the Declaration of Helsinki and ethics committee requirements.

Operational Process

The process follows four essential steps: explanation in an independent setting → full understanding → signing of the informed consent form (ICF) → provision of a copy with documentation.

The explanation provided to participants or their guardians must cover:

- Research objectives (investigation of microbiome dynamics).
- Types of samples to be collected (skin, intestinal, and oral swabs, etc.).
- Planned sampling timepoints.
- Privacy protection measures (use of coded identifiers instead of names).
- Voluntary withdrawal rights.

The signing requirements include:

- Dual signatures by the participant and guardian (if the participant lacks capacity).
- Witness signature on the ICF when participants are illiterate or unable to sign.
- Research personnel must sign the “Disclosure Statement.”

Informed Consent Signing and Management Procedure Special Circumstances:

- Emergency sampling (sepsis): Collect sample first → obtain informed consent within 24 hours.
- Participant withdrawal: Complete the “Withdrawal Declaration Form.” Already collected samples must be processed as follows: (✓ retained if continued use is permitted; ✓ destroyed if use is refused).
- Secondary use of samples: Indicate agreement (“Yes □/No □”) for use in other research on the ICF signature page.

Informed Consent Signing and Management Procedure Documentations:

- Original Signed ICF: The paper copy of the originally signed ICF is stored in a dedicated, double-locked filing cabinet at the study site, with access strictly controlled and logged. An electronic, scanned copy is created and encrypted using the AES-256 standard for secure backup and authorized review.
- Withdrawal Declaration Form: In the event a participant withdraws consent, the completed withdrawal form is archived separately from active participant files and is clearly labeled with a "Withdrawn" status to prevent inadvertent use of data or samples.
- Sample Usage Authorization Record: The participant's choice regarding the secondary use of biospecimens is definitively recorded in the study database. The record is annotated as either "Authorized for secondary use (coded sharing)" or "Refused (sample destruction confirmed)" based on the ICF.

Informed Consent Signing and Management Procedure Quality Documentations

- **Completeness of Signatures:** To ensure full comprehension and agreement, a quarterly random inspection of 10% of all ICFs is conducted. The compliance standard requires that 100% of inspected forms must have all critical clauses signed by both the participant and investigator, with no omissions.
- **Timeliness of Emergency Re-consenting:** For participants who develop sepsis (an event that may alter the risk-benefit profile), a full audit is performed. The compliance standard requires that 100% of necessary re-consenting procedures in such cases must be completed within 24 hours of the patient regaining capacity or a legal representative being available.
- **Privacy Protection:** The security of stored ICFs, both physical and electronic, is subject to review by the Data and Safety Monitoring Board (DSMB). The compliance standard is the occurrence of zero data leakage events from the ICF storage systems.

### **SOP04: Collaboration and Communication Mechanisms Between Study Centers**

Scope: Principal Investigators (PIs), Sub-Investigators (Sub-Is), Co-Investigators (Co-Is), and sampling personnel at each clinical center.

Purpose: This SOP aims to ensure standardized operations and data homogeneity across 36 research centers, while enabling timely resolution of inter-center issues.

Organizational Structure

Lead Institution → Center PI → Sampling Team → Center Laboratory → Data Center

Collaboration Workflow

**Routine Collaboration and Communication Mechanisms Between Study Centers**

- Consumables distribution: Conducted quarterly via cold-chain transportation with electronic receipt confirmation; output is the Batch Consumables Report.
- Data synchronization meeting: Held monthly through online meetings (e.g., Tencent Meeting); output is the Inter-center Compliance Statistics.
- Protocol updates: Implemented immediately via encrypted email with electronic confirmation receipt; output is the Confirmation of Implementation of Revised Provisions.

Emergency Event Handling: Problem classification:

- Technical/operational issue: Quality Control Team responds within 72 hours.
- Ethical risk: Referred to the Lead Unit Ethics Committee.
- Data security risk: Escalated to the Data and Safety Monitoring Board (DSMB).

**Key Control Points of Collaboration and Communication Mechanisms Between Study Centers**:

- **Additional sepsis sampling: Within 24 hours of diagnosis, the center must report the patient code, and the lead institution must trigger synchronization across all centers.**
- **Batch effect control: Mandatory use of quarterly-distributed consumables (nucleic acid preservation solution/sample tubes); replacement with other brands is prohibited.**
- **Execution of negative controls: All centers must simultaneously conduct the C1–C5/M1 procedures and upload videos to the shared repository.**
- **Data de-identification & transfer: Sample coding must follow the format: BIOME–CenterID–SubjectID–Site–Time–Replicate.**

Examples of coding format:

- BIOME-CHYY01-NS-Limb-D1-REP1: Normal skin, limb, Day 1 post-burn, 1st replicate
- BIOME-CHYY01-NS-Trunk-D1-REP1: Normal skin, trunk, Day 1 post-burn, 1st replicate
- BIOME-CHYY01-BW-Limb-D1-REP1: Burn wound, limb, Day 1 post-burn, 1st replicate
- BIOME-CHYY01-BW-Trunk-D1-REP1: Burn wound, trunk, Day 1 post-burn, 1st replicate
- BIOME-CHYY01-SS-D1-REP1: Stool sample, Day 1 post-burn, 1st replicate
- BIOME-CHYY01-AS-D1-REP1: Anal swab, Day 1 post-burn, 1st replicate
- BIOME-CHYY01-OS-D1-REP1: Oral swab, Day 1 post-burn, 1st replicate

Communication Tools and Access Rights of Collaboration and Communication Mechanisms Between Study Centers

- Enterprise WeChat: Used for routine communication; accessible to all study personnel.
- Encrypted cloud drive: For sharing SOPs and instructional videos; access granted to Center PIs and sampling staff.
- Electronic data system: Enables CRF entry and sample tracking; access is role-based for authorized personnel.
- Emergency contact network: Provides 24-hour response to sepsis events; includes the lead unit dispatcher and Center PIs.

Quality Records

- Inter-center Issue Log: Contains problem description, resolution, and closure date; maintained by the lead unit.
- Consumables Receipt Form: Records batch ID, quantity, temperature logs, and recipient; held by each center and the lead unit.
- Data Consistency Audit Report: Contains analysis of inter-center contamination rates and timing deviations; maintained by the DSMB.

**Emergency Event Reporting Template**

Center ID: __________

Event type: □ Sepsis diagnosis □ Operational incident □ Data breach

Patient code: __________

Description: ________________________

Request for support: ________________________

**Consumables Anomaly Feedback**

Temperature excursion during transport: Photograph evidence → Reject shipment → Lead unit redistributes

Packaging damage: Video documentation → Use backup consumables → Trace responsible party

### **SOP05: Management of Study Documents and Materials**

Scope: All clinical center PIs, Sub-Investigators (Sub-Is), Co-Investigators (Co-Is), and sampling personnel

Purpose: To ensure that all research documents are stored in compliance with regulatory requirements for at least ten years, safeguard participant privacy, and provide independent oversight by the Data and Safety Monitoring Board (DSMB).

Document Classification and Management Standards

- Core Documents: Includes the protocol, ethics approvals, and original informed consent forms; accessible only to the Lead PI; stored as hard copies in a dual-lock cabinet and electronic copies encrypted via AES-256.
- Process Documents: Includes case report forms (CRFs), sample transport records, and negative control logs; accessible to the Site PI and Quality Control officer; managed within an electronic database with field-level access control.
- Source Data: Consists of raw metagenomic sequencing data (FASTQ format); accessible to Data analysts and the DSMB; transferred via SSH File Transfer Protocol (SFTP) and encrypted with PGP.
- DSMB Oversight Files: Includes quarterly safety reports and data breach audit logs; accessible only to DSMB members; stored on an independent, physically isolated server.

DSMB Oversight Workflow

Responsibilities:

- Data Security: Quarterly audits of de-identification compliance (coding rule: Center ID – Subject ID – Sample type – Timepoint).
- Risk Monitoring: Trigger alerts if sample contamination rate > 5% or if any privacy breach occurs.
- Study Progress: Initiate corrective recommendations if dropout rate exceeds 20%.

Operational Workflow:

- Data manager submits quarterly progress report → DSMB closed session review → Major risk identified?
- Yes: DSMB issues recommendation to suspend study.
- No: Lead unit responds within 72 hours and signs a “DSMB Approval Statement.”

**Key Privacy Protection Measures**:

- **Data transfer leakage: Controlled via the Medical Digital Identity System (MDIS) combined with Access Control Lists (ACL).**
- **Paper record leakage: Prevented through the use of infrared surveillance in archives and a biometric entry system.**
- **Sample coding linkage: Managed by a triple mapping system: (1) Name → Hospital ID, (2) Hospital ID → Pseudocode, (3) Pseudocode → Study ID.**

Examples of Study ID coding format:

- BIOME-CHYY01-NS-Limb-D1-REP1: Normal skin, limb, Day 1 post-burn, 1st replicate
- BIOME-CHYY01-NS-Trunk-D1-REP1: Normal skin, trunk, Day 1 post-burn, 1st replicate
- BIOME-CHYY01-BW-Limb-D1-REP1: Burn wound, limb, Day 1 post-burn, 1st replicate
- BIOME-CHYY01-BW-Trunk-D1-REP1: Burn wound, trunk, Day 1 post-burn, 1st replicate
- BIOME-CHYY01-SS-D1-REP1: Stool sample, Day 1 post-burn, 1st replicate
- BIOME-CHYY01-AS-D1-REP1: Anal swab, Day 1 post-burn, 1st replicate
- BIOME-CHYY01-OS-D1-REP1: Oral swab, Day 1 post-burn, 1st replicate

Regulatory Compliance and Registration

The BIOME study strictly adheres to Good Clinical Practice (GCP) and ethical research principles. The project has been approved by the Ethics Committee and registered with national authorities:

- Ethics Approval Number: CHEC2025-071
- Clinical Trial Registration: ChiCTR2500099489
- National Human Genetic Resources Administration Filing: MR-31-25-023993

# **Sample Collection and Processing SOPs**

### **SOP06: Standardized Procedures for** **Skin Swab Collection, Preservation, and Transportation**

Scope: Principal Investigators (PIs) at each clinical center, Sub-Investigators (Sub-Is), Co-Investigators (Co-Is), sampling personnel, sample quality control staff, central laboratory staff.

Purpose: This SOP aims to standardize the collection, preservation, and transportation of skin swab samples, ensuring that specimen quality meets analytical requirements while minimizing operational errors.

Specimen Priority

- Primary specimen: Skin swab samples.
- Alternative specimens: None.

Skin Swab Sample Collection Required Materials

- **Skin swab kit:** Sterile cotton swab with plastic handle and pre-filled cryovial.
- **Disposable sterile gloves:** Powder-free.
- **Patient identification label:** Made of waterproof material.
- **Ultra-low temperature freezer:** -80℃, confirm storage capacity in advance.

Procedure

Pre-collection preparation:

- Record any recent use of antibiotics, as they may influence the human microbiome.
- This procedure applies to all burn patients undergoing skin sampling, and sampling must be performed before wound debridement or dressing change.
- Eligible sampling sites must include both burned and unburned skin regions of at least 5 × 5 cm² each.
- Explain the procedure to the patient, wear double sterile gloves, and moisten the swab tip with sterile water or normal saline.

Sampling sites:

- Burned region of the trunk
- Unburned region of the trunk
- Burned region of the radial side of extremities
- Unburned region of the radial side of extremities

Sampling steps:

- Perform hand hygiene and wear sterile protective equipment.
- Ensure that sampling occurs prior to debridement or dressing change. Rinse the site with sterile saline or wipe gently with sterile gauze.
- Using a saline-moistened swab, rub the sampling area repeatedly for at least 30 seconds.
- Insert the swab into a cryovial pre-filled with preservation solution. Break the swab at the pre-scored breakpoint, seal the tube tightly, and disinfect the exterior with an alcohol pad.
- Repeat the above sampling steps three times. Each specimen should include three swabs, stored in three separate cryovials containing preservation solution, each properly labeled.
- Immediately freeze the tubes in liquid nitrogen or on dry ice, and transfer to -80°C storage within one hour.
- Record the sampling date, time, and anatomical site in the case record.

Wound healing evaluation:

To ensure data consistency and quality, we have refined our protocol with strict definitions and a rigorous multi-level adjudication mechanism:

(1) Standardized Sampling Area: First, we wish to clarify that our skin microbiome sampling is strictly confined to a fixed 5 × 5 cm² area of a deep second-degree burn wound for each patient. This standardization eliminates variability caused by sampling different wound depths or sizes.

(2) Definition of "Wound Healing": "Healing" in our study specifically refers to the complete epithelial closure of this sampled area, regardless of whether the wound heals spontaneously or via grafting. Given the scarcity of autogenous donor skin in MBI patients (>50% TBSA), large sheet grafting is rarely feasible; instead, widely expanded mesh grafting or stamp grafting is the standard of care across all centers. Therefore, we define "healing" based on the specific modality:

- Spontaneous Healing: Defined as the complete epithelialization and closure of the wound.
- Grafted Healing (Mesh/Stamp): Defined as the survival of the grafted skin islands combined with the complete epithelialization of the interstices (gaps) between them, resulting in total wound closure.

(3) Centralized Adjudication Process:

To unify judgment standards across 50 centers, we have implemented a mechanism for all key clinical events (enrollment, sepsis diagnosis, and wound healing):

- Joint Determination: Each event is independently assessed by two senior clinicians: the Principal Investigator (PI) at the local center and a designated expert from the lead center (The First Affiliated Hospital of Naval Medical University).
- Arbitration: In cases of disagreement, a third senior clinician from the Independent Adjudication Committee provides the final judgment.
- EDC & Image Review: High-resolution photographs of the sampling sites and diagnostic evidence are uploaded to the Electronic Data Capture (EDC) system. The Independent Adjudication Committee conducts a final comprehensive review of all records before database lock to ensure strict adherence to criteria.

Sample Preservation and Transportation:

- Immediate Handling: Following collection, cryopreservation must be performed in liquid nitrogen or on dry ice within 15 minutes or less.
- Storage Conditions: Samples must be stored at -80℃. The use of cryoprotectants is strictly prohibited.
- Transportation: Samples must be transported with dry ice. All specimens must be labeled in accordance with SOP05. Shipment records are to be submitted to the shared group. Upon receipt, the central laboratory must perform quality control (QC) checks and provide feedback within 24 hours.

Key Considerations

- Timing control: Sampling must adhere strictly to the study schedule.
- Contamination prevention: Avoid swab contact with skin outside the designated sampling area.
- Biosafety: Dispose of gloves, swab wrappers, and other consumables as medical waste.

Quality Records

- Sample labeling: Patient ID, date/time of sampling, sample type (skin swab).
- Rejection criteria:
  - Sample desiccation or leakage
  - Illegible or missing labels
  - Storage temperature not meeting protocol requirements
- Sample Invalidity Criteria
  - Major failure: No cryopreservation in liquid nitrogen/dry ice within 15 minutes
  - Moderate failure: Inadequate sample material (swab with no visible collection)
  - Minor failure: Sequencing quality not qualified (validation through sequencing required, aside from Major failure and Moderate failure cases).

### **SOP07: Standardized Procedures for Intestinal Samples (Stool Samples/Anal Swabs) Collection, Preservation, and Transportation**

Scope: Principal Investigators (PIs) of each clinical center, Sub-Investigators (Sub-Is), sampling personnel, sample quality controllers, central laboratory staff.

Purpose

- To standardize dietary instructions for patients prior to stool sample collection, thereby minimizing dietary influences on detection results and improving accuracy and reliability.
- To standardize the collection, preservation, and transportation of stool and anal swab samples, ensuring sample quality meets testing requirements and minimizing operational errors.

Sample Selection Priority

- Preferred sample: Fresh stool samples.
- Alternative sample: Anal swabs, in cases where spontaneous defecation is not possible.

Intestinal Sample Collection Required Materials

- **Sterile stool collection container:** Leak-proof with a screw-cap.
- **Anal swab kit:** Contains a sterile swab with a plastic handle and a preloaded cryotube with preservation solution.
- **Disposable sterile gloves:** Powder-free.
- **Patient identification labels:** Made of waterproof material.

**Ultra-low temperature freezer:** -80℃, Storage capacity must be pre-confirmed.Procedures

Dietary Preparation

- For burn patients unable to eat orally, use standard burn nutrition support protocols.
- Ensure sufficient nutrition intake: Nutrition and fluid management follow clinical guidelines appropriate for the patient’s phase of care (e.g., enteral or parenteral nutrition during the acute phase). Protocols regarding specific oral fluid (1.5–2 L/day) and dietary fiber intake are encouraged only when clinically feasible and tolerated by the patient, primarily during the rehabilitation phase.
- Clear liquid or semi-liquid diets may be given for absorption issues; suspend oral feeding during severe diarrhea.
- Fecal Occult Blood Test (FOBT) diet: For 3 days prior, restrict foods causing false positives (meat, poultry, iron-rich foods, green vegetables). Allow milk, soy products, potatoes, radish, rice, steamed bread, etc. Collect stool on Day 4.
- Creatinine Test: For 3 days prior, prohibit meat, poultry, fish, tea, and coffee; restrict protein <40 g/day. Allow vegetables, fruits, plant oils. Collect 24 h urine for creatinine clearance and 2 ml anticoagulated blood for plasma creatinine.

Stool Sample Collection

- Patient defecates into a clean, dry container or collection paper (avoid urine, water, disinfectants).
- Open stool DNA preservation tube (avoid spilling solution/steel beads). Use provided spatula to transfer 2–5 g stool (size of soybean/peanut).
- Replace spatula and tighten lid. Shake vigorously ≥ 30 s. Wipe exterior with alcohol swab.

Anal Swab Collection

- Indications: When patient cannot defecate or stool volume is insufficient.
- Preparation: Clean perianal area with water. Explain procedure; patient assumes left lateral position with knees flexed. Wear double gloves and lubricate swab tip with sterile water/saline.
- Steps: Open sterile swab package, hold plastic handle only. Gently insert swab 2–3 cm along rectal wall. Rotate ≥ 3 times, hold 10 s. Withdraw slowly, confirm fecal matter adhered (QC point). Break swab at preset notch, drop into cryotube with preservation solution, tighten cap.
- Repeat thrice (3 swabs → 3 tubes). Label accordingly. Freeze immediately in liquid nitrogen/dry ice, transfer to -80°C within 1 h. Record patient ID, date, time, and site.

Intestinal Sample Preservation and Transportation:

- Immediate Handling: Following collection, cryopreservation must be performed in liquid nitrogen or on dry ice within 15 minutes or less.
- Storage Conditions: Samples must be stored at -80℃. The use of cryoprotectants is strictly prohibited.
- Transportation: Samples must be transported with dry ice. All specimens must be labeled in accordance with SOP05. Shipment records are to be submitted to the shared group. Upon receipt, the central laboratory must perform quality control (QC) checks and provide feedback within 24 hours.

Key Considerations

- Gastrointestinal Dysfunction: MBI patients frequently suffer from paralytic ileus due to burn shock, systemic inflammation, and the high-dose use of opioid analgesics during the acute phase (especially the first 72 hours). Consequently, many patients cannot produce a stool sample during the strictly defined, short sampling windows (e.g., Day 1, 3, 7).
- Feasibility in ICU (Rescue Method): Anal swabs provide a standardized, controllable means to obtain intestinal microbiota samples when spontaneous defecation is absent, ensuring that we capture the microbiome status during these physiologically critical windows. Excluding these time points would render the longitudinal trajectory incomplete and biased toward less severe cases.
- Standardized Sampling Hierarchy and Protocol: To mitigate the potential bias introduced by mixed sampling methods, we have established a strict, prioritized protocol and management strategy. We explicitly state that Stool Sample is the Gold Standard. Anal swabs are permitted only when the patient has not defecated within the sampling window. When a patient is due for sampling (e.g., 3 ± 1 days), and spontaneous stool is unavailable, an anal swab is collected. However, if the patient subsequently produces a stool sample within the 3 ± 1 days following the swab collection, the stool sample will be prioritized for sequencing analysis, and the swab sample will be relegated to backup or quality control. This prioritizes the gold standard (stool) whenever clinically possible, even if delayed slightly.
- Metadata Tracking and Control: The sample type (Stool vs. Swab) is a mandatory field in our Electronic Data Capture (EDC) system. This ensures comprehensive tracking.
- Statistical Correction: In our downstream bioinformatics analysis, "Sample Type" will be included as a fixed covariate to adjust for potential batch effects. We have also planned a Sensitivity Analysis to compare the microbial diversity and compositional profiles between paired samples or stratified groups to quantify and correct for any systematic bias.
- Timing control: Sampling must adhere strictly to the study schedule.
- Contamination prevention: Avoid swab contact with skin outside the designated sampling area.
- Biosafety: Dispose of gloves, swab wrappers, and other consumables as medical waste.

Quality Records

- Sample labeling: Patient ID, date/time of sampling, sample type (skin swab).
- Rejection criteria:
  - Sample desiccation or leakage
  - Illegible or missing labels
  - Storage temperature not meeting protocol requirements
- Sample Invalidity Criteria
  - Major failure: No cryopreservation in liquid nitrogen/dry ice within 15 minutes
  - Moderate failure: Inadequate sample material (swab with no visible collection)
  - Minor failure: Sequencing quality not qualified (validation through sequencing required, aside from Major failure and Moderate failure cases).

### **SOP08: Standardized Procedures for Oral Swab Collection, Preservation, and Transportation**

Scope: Principal Investigators (PIs) at clinical centers, Sub-Investigators (Sub-Is), sampling staff, sample quality control staff, central laboratory staff.

Purpose: This SOP aims to standardize the procedures for the collection, preservation, and transportation of oral swab samples to ensure sample quality meets testing requirements and to minimize operational errors.

Sample Priority

- Preferred sample: Oral swab sample.
- Alternative sample: None.

Oral Swab Sample Collection Required Materials

- Oral swab kit: Contains a sterile swab with a plastic handle and a preloaded cryotube with preservation solution.
- Disposable sterile gloves: Powder-free.
- Patient identification labels: Made of waterproof material.
- Ultra-low temperature freezer: -80℃, Storage capacity must be pre-confirmed.

Procedures

Oral hygiene preparation:

- No eating, drinking, smoking, chewing gum, or betel nut, and no brushing teeth or using mouthwash within 1 hour prior to sampling.
- Remove dentures if present.
- Clean oral cavity: 30 minutes before sampling, rinse vigorously with ~30 mL of room-temperature water, swishing around cheeks, palate, and under the tongue three times (10 seconds each), then spit out completely.
- Throat rinse: Tilt head back, say “ah,” and gargle with ~20 mL of water for 10 seconds, then spit out. Repeat once (adults only, if cooperation possible).
- Residue removal: Inspect the mouth for visible food debris; gently wipe with sterile swab if necessary.
- Rest 15–30 minutes to allow the oral cavity to dry naturally, with no oral activity during this period.

Pre-sampling preparation:

- Review patient’s oral health history and collect relevant information.
- Record antibiotic usage time and type, as antibiotics may affect microbiota.
- Confirm patient has no unusual symptoms compared with their usual health status.
- Within 30 minutes prior to sampling: wash hands, rinse mouth 1–2 times with clean water, and swallow residual water.
- Within 30 minutes prior to sampling: avoid food, drink, smoking, or chewing gum.

Sampling sites:

- Dorsal tongue: scrape central area ~1 cm² for ~5 seconds.
- Hard palate: scrape entire hard palate for ~10 seconds.
- Buccal mucosa: scrape both left and right buccal mucosa for ~10 seconds each.
- Oropharynx: gently extend swab to posterior pharyngeal wall and swab for ~5 seconds (avoid tonsils).
- Additional sampling sites may be included based on study requirements.

Sampling operations:

- Wash hands before sampling and open sterile swab packaging.
- Insert swab into oral cavity and wipe designated areas ≥ 20 times until saturated with saliva (no debris should remain attached).
- If required, moisten the swab with sterile saline and swab target areas for at least 30 seconds.
- Place the swab into a cryotube containing preservation solution, break at the pre-scored mark, seal tube tightly, and disinfect exterior with alcohol swab.
- Repeat the process three times: each sample consists of three oral swabs, stored in three separate cryotubes with labels.
- Immediately freeze in liquid nitrogen or dry ice, then transfer to -80 °C freezer within 1 hour.
- Record sampling date, time, and site.

Oral Swab Sample Preservation and Transportation:

- Immediate Handling: Following collection, cryopreservation must be performed in liquid nitrogen or on dry ice within 15 minutes or less.
- Storage Conditions: Samples must be stored at -80℃. The use of cryoprotectants is strictly prohibited.
- Transportation: Samples must be transported with dry ice. All specimens must be labeled in accordance with SOP05. Shipment records are to be submitted to the shared group. Upon receipt, the central laboratory must perform quality control (QC) checks and provide feedback within 24 hours.

Key Considerations

- Timing control: Sampling must adhere strictly to the study schedule.
- Contamination prevention: Avoid swab contact with skin outside the designated sampling area.
- Biosafety: Dispose of gloves, swab wrappers, and other consumables as medical waste.

Quality Records

- Sample labeling: Patient ID, date/time of sampling, sample type (skin swab).
- Rejection criteria:
  - Sample desiccation or leakage
  - Illegible or missing labels
  - Storage temperature not meeting protocol requirements
- Sample Invalidity Criteria
  - Major failure: No cryopreservation in liquid nitrogen/dry ice within 15 minutes
  - Moderate failure: Inadequate sample material (swab with no visible collection)
  - Minor failure: Sequencing quality not qualified (validation through sequencing required, aside from Major failure and Moderate failure cases).

### **SOP09: Standard Operating Procedures for Blood Sample Collection**

Scope: Principal Investigators (PIs), Sub-PIs, Sampling Personnel, Sample Quality Control Staff, Central Laboratory Personnel.

Purpose: To standardize the collection, preservation, and transportation of blood samples, ensuring sample quality meets testing requirements and minimizing operational errors.

Sample Selection Priority

- Preferred sample: Blood samples.
- Alternative sample: None.

Required Materials

Blood Sample Collection Required Materials

- **Sterile blood collection needle:** Ethylene oxide sterilized, sterile.
- **2% Chlorhexidine gluconate alcohol solution:** Contains chlorhexidine (18.0 – 22.0 g/L) and ethanol (63% – 77%).
- **Cotton swabs:** Ethylene oxide sterilized, sterile.
- **Tourniquet:** Disposable.
- **Medical adhesive plaster:** Ethylene oxide sterilized, single-piece.
- **Disposable sterile gloves:** Powder-free rubber gloves.
- **Patient identification labels:** Waterproof material.
- **Blood collection tubes:** Type selected according to specific blood sample requirements.
- **Refrigerator at 4**℃**:** Capable of low-temperature storage.

Procedures

Pre-collection Preparation

- Ensure the patient is in normal physical condition on the day of collection (no abnormal symptoms compared to baseline).
- Instruct the patient to refrain from eating, drinking, smoking, or other activities within 30 minutes prior to sampling.

Blood Collection Steps

- Assist the patient into a comfortable supine or seated position; wear sterile gloves.
- Apply a tourniquet 6–10 cm above the puncture site, with the tail end facing upward.
- Disinfect the skin at the puncture site twice with chlorhexidine alcohol, ensuring each round dries completely; disinfection area should be > 5 cm in diameter.
- Instruct the patient to clench their fist. Use the non-dominant hand to tighten the skin. Insert the needle into the vein at a 15°–30° angle along the vein path. Confirm blood return, then advance the needle until the bevel is fully inside the vein. Secure the needle and puncture site with sterile adhesive plaster.
- Attach the blood collection tube. Once blood flows smoothly, instruct the patient to relax their fist. Change tubes as required. After each tube is filled, invert vertically 180° several times. (Note: Anticoagulant tubes must not be the first tube collected. If only coagulation indicators are needed, discharge air at the tube tip before collection.)
- Release the tourniquet, carefully remove the adhesive plaster, gently press the puncture site with a sterile swab or adhesive plaster, and withdraw the needle swiftly. Apply pressure for 1–2 minutes until bleeding stops.
- Place the samples immediately in a 4°C refrigerator and deliver promptly to the laboratory for testing.

Key Considerations

- Timing control: Follow the sampling time points as defined in the research protocol.
- Contamination prevention: Avoid hemolysis (rupture of red blood cells that may interfere with analysis).
- Biosafety: Use sterile instruments throughout; ensure strict disinfection of medical equipment.

### **SOP10: Sampling Methods for Negative and Positive Control Specimens**

Scope: Principal Investigators (PIs) at clinical centers, Sub-Investigators (Sub-Is), sampling staff, sample quality control staff, central laboratory staff.

Purpose: The objective of this SOP is to establish standardized procedures for the collection, preservation, and transportation of environmental samples in Intensive Care Units (ICUs), as well as the collection of negative and positive control specimens. The purpose is to:

- Ensure the quality and integrity of samples collected for microbiological, metagenomic, and molecular analyses.
- Provide a uniform methodology across multiple centers to minimize variability and sampling bias.
- Define standardized practices for control specimens to detect potential contamination, validate experimental processes, and ensure data reliability.

Sample Priority

Primary Specimens:

- ICU environmental surface swabs (high-touch surfaces, bedside equipment, door handles).
- Airborne particulate samples (passive sedimentation or active air filtration).
- Negative and positive control samples as outlined below.

Alternative Specimens:

- None defined. All listed sample categories are mandatory for comprehensive quality control.

Negative and Positive Control Specimen Collection Required Materials

- **Sterile swab kits**: Sterile cotton/polyester swabs with plastic handle, pre-loaded cryotubes with DNA/RNA preservation solution.
- **Air sampling device**: Sterilized inlet heads, cutting heads, and membrane holders.
- **Sterile filter membranes**: Glass fiber filters, sterile, diameter 80 mm.
- **Passive exposure tools**: Sterile hanging hooks, 10 × 10 cm sterile sampling templates.
- **Disposable sterile gloves**: Powder-free, nitrile or latex.
- **Patient/Specimen ID labels**: Waterproof, alcohol-resistant.
- **Cryogenic freezer**: -80℃, pre-confirmed storage capacity.
- **Dry ice or liquid nitrogen**: For immediate freezing and cold-chain transport.
- **Transport boxes**: Insulated containers certified for -70℃ transport.

Sampling Procedures

- Environmental Surface Sampling
- Target Surfaces:
- Bed rails (frequently contacted upper surface).
- Infusion pump (main control panel/buttons).
- Ventilator interface (touchscreen or control interface).
- Bedside table (upper surface).
- Interior door handle (used for exiting the patient room).

Procedure:

- Don sterile gloves.
- Place a sterile 10 × 10 cm sampling template flat on the designated surface.
- Open a sterile swab package. Moisten the swab by dipping it into the pre-labeled tube containing DNA/RNA preservation buffer. Remove excess liquid by pressing against the tube wall.
- Swab the template area for 30 seconds using medium, consistent pressure, first horizontally in a zigzag “Z” path, then rotate the swab and repeat vertically.
- Immediately place the swab into the tube, break at the scored handle point, and close tightly.
- Repeat for each of the five mandatory surfaces, using fresh swabs, gloves, templates, and tubes each time.
- Vortex the tube for 60 seconds and transfer to -80 °C storage.
- Passive Air Sedimentation Sampling

Preparation:

- Label tubes with “Air Sampling” ID.
- Suspend sterile swabs horizontally using sterile plastic hooks at ~1 m above patient head level (e.g., IV pole or monitor stand).

Procedure:

- Expose swabs for 10 minutes in the ICU room with doors closed and minimal staff movement.
- Record environmental disturbances (bed sheet changes, suctioning, multiple staff entries) in the electronic CRF.
- Retrieve swabs aseptically, break handles, seal tubes, vortex for 60 seconds, and transfer to -80 °C storage.

Active Air Filtration Sampling (Optional)

- Assemble the air sampler with sterile inlet head, cutting head, and membrane holder.
- Insert sterile glass-fiber filter (80 mm diameter).
- Run device at 1 L/min airflow for 1–2 hours.
- Transfer filter membrane to sterile cryotube, seal, disinfect exterior, and freeze at -80 °C.

Positive and Negative Control Specimen Coding and Collection Methods

- **C1: Unopened preservation solution tube.**
- **C2: Preservation tube briefly opened for 1 second and then closed.**
- **C3: Sterile swab tip cut with sterile scissors and placed into preservation solution.**
- **C4: Sterile swab exposed to ICU air for 15 seconds, then the tip is cut and stored.**
- **C5: Sterile swab rubbed 5 times across sampling gloves, then the tip is cut and stored.**
- **M1: Swab dipped into a thawed mock microbial sample (stored at -80**℃**), exposed to air for 15 seconds, and then stored in a preservation tube.**

Positive and Negative Control Specimen Preservation and Transportation

- Immediate Handling: Following collection, cryopreservation must be performed in liquid nitrogen or on dry ice within 15 minutes or less.
- Storage Conditions: Samples must be stored at -80℃. The use of cryoprotectants is strictly prohibited.
- Transportation: Samples must be transported with dry ice. All specimens must be labeled in accordance with SOP05. Shipment records are to be submitted to the shared group. Upon receipt, the central laboratory must perform quality control (QC) checks and provide feedback within 24 hours.

Key Considerations

- Timing control: Sampling must adhere strictly to the study schedule.
- Contamination prevention: Avoid swab contact with skin outside the designated sampling area.
- Biosafety: Dispose of gloves, swab wrappers, and other consumables as medical waste.
- Environmental Monitoring: Record disturbances during passive air sampling.
- Control Compliance: Negative and positive controls are mandatory per sampling session.

Quality Records

- Sample labeling: Patient ID, date/time of sampling, sample type (skin swab).
- Rejection criteria:
  - Sample desiccation or leakage
  - Illegible or missing labels
  - Storage temperature not meeting protocol requirements
- Sample Invalidity Criteria
  - Major failure: No cryopreservation in liquid nitrogen/dry ice within 15 minutes
  - Moderate failure: Inadequate sample material (swab with no visible collection)
  - Minor failure: Sequencing quality not qualified (validation through sequencing required, aside from Major failure and Moderate failure cases).
- Archival Records: ICU environmental sampling sheets must be archived monthly, with signatures, corrections properly logged, and electronic copies uploaded to secure storage.

### **SOP11: Sample Transportation, Receiving Registration and Storage Procedures**

Scope: Principal Investigators (PIs) at clinical centers, Sub-Investigators (Sub-Is), sampling staff, sample quality control staff, central laboratory staff.

Purpose: This SOP is established to:

- Ensure the integrity and traceability of biological samples during cold-chain transportation.
- Standardize the receiving and registration process at central laboratories.
- Define proper storage and inventory management procedures for various sample types (DNA, tissues, libraries, and intermediate products).
- Mitigate risks associated with sample degradation, contamination, or mismanagement.
- Guarantee that all samples remain compliant with study protocols and regulatory requirements.

Required Materials of Sample Transportation, Receiving Registration and Storage Procedures

- Dry Ice: Must be pelletized and sourced from a certified supplier, with sufficient quantity for the expected transportation duration.
- Foam Box (Insulated Container): Requires a wall thickness of ≥ 4 cm and an inner volume of ≥ 8 – 10 times the sample volume.
- Plastic Cushioning: To be used to prevent breakage or leakage during transport.
- Waterproof Patient ID Labels: Must be resistant to low temperatures and abrasion.
- Laboratory Information System (LIS) Access: Required for sample registration and verification.
- Ultra-low Freezers: Requires a -80℃ freezer for RNA, tissues, and long-term storage, and a -20℃ freezer for DNA and libraries.
- Cold Packs / Ice Packs: For samples that require temporary storage at 4℃.
- Personal Protective Equipment (PPE): Includes gloves, lab coats, protective goggles, and masks.

Cold-Chain Transportation Process

Preparation of Dry Ice

- Calculate the required dry ice quantity based on sample size and transportation distance. Example: For a 2000 km shipment with a sample box size of 10 × 28 × 28 cm, ≥ 20 kg of dry ice is required.
- Always purchase dry ice from a certified and compliant supplier to ensure purity and safety.
- Handle dry ice using insulated gloves and face shields to prevent frostbite.

Preparation of Foam Boxes

- Choose foam boxes with internal dimensions 8–10 × larger than sample volume to allow for sufficient dry ice.
- Ensure wall thickness ≥ 4 cm to maintain insulation and prevent external damage.
- Inspect boxes for cracks, deformities, or moisture before use.

Sample Packaging Principles

- Place dry ice on all six sides (top, bottom, front, back, left, right) of the sample tubes.
- Wrap sample tubes in shock-proof plastic to avoid cracking during transit.
- Seal both internal and external packaging with adhesive tape to prevent leakage.
- Place an absorbent sheet inside the box to contain possible spills.

Shipment and Courier Selection

- Preferred Courier: SF Express (or equivalent) for compliance and traceability.
- Record the logistics tracking number immediately after dispatch.
- Enter the shipment details into the LIS system, linking tracking numbers with sample IDs.

Sample Receiving and Registration Procedures

- Delivery and Acceptance
- Upon courier arrival, receiving staff must:
- Inspect package integrity (no damage, leakage, or thawing).
- Sign and document courier receipt.
- Transport package to the receiving area immediately.

Laboratory Receiving Workflow

- Receiving staff must wear PPE (lab coat, gloves, mask, and shoe covers).
- Log in to the LIS system and navigate to: Production Management → Sample Center → Receiving Audit → Pending Review.
- Match shipment tracking numbers with the LIS database.
- Record any discrepancies (damage, missing samples, incorrect labeling).

Sample Quality Check

- Check packaging: Integrity and dryness.
- Check labeling: Clear, waterproof ID labels.
- Check temperature: Confirm samples remained within designated temperature range.
- Photograph abnormalities and upload them to the LIS system.

Sample Labeling and Storage Assignment

- Assign a unique production ID to each sample tube.
- Apply labels on both tube caps and walls.
- Photograph all samples under a high-resolution scanner for archiving.
- Store samples in designated boxes within fixed freezer compartments.

Exception Handling

If discrepancies arise (quantity mismatch, mislabeling, thawing), staff must:

- Record in LIS remarks.
- Upload photographs of abnormalities.
- Reject the batch if necessary, pending resubmission by the sender.

Sample Storage Management

Temporary Storage

- Before handover to laboratory staff: Samples remain in dry ice boxes until processed.
- During production workflow: Keep samples on dry ice or in appropriate freezers whenever not actively used.

Long-term Storage

- **Original Tissues & RNA: Store in a -80**℃ **ultra-low freezer.**
- **Original DNA Samples: Store in a -20**℃ **freezer.**
- **Original Libraries: Store in a -20**℃ **freezer.**
- **Extracted RNA: Store in a -80**℃ **freezer.**
- **Extracted DNA: Store in a -20**℃ **freezer.**
- **In-house Libraries: Store in a -20**℃ **freezer.**
- **Intermediate Products: Store in a -20**℃ **freezer.**

Temperature Control

Freezer logs must be recorded twice daily (morning & evening).

- Acceptable temperature ranges: -80 °C freezers: -60 °C to -80 °C. -20 °C freezers: -16 °C to -24 °C.
- Annual calibration of thermometers and sensors required.
- Alarm system must alert personnel in case of deviation.

Inventory Management

- Segregate storage areas by sample type (tissues, DNA, RNA, libraries, QC libraries, sequencing-ready libraries).
- Apply clear labeling on freezer racks, sample boxes, and compartments.
- Document storage location in LIS using: Tissue Storage Log; Nucleic Acid Sample Location Log; QC Library Log; Sequencing Library Log.
- Every retrieval or return must be photographed and documented.

Key Considerations

- Timeliness: For urgent samples, the central laboratory must process within 24 hours of arrival.
- Contamination Prevention: Avoid cross-contamination during packaging and handling.
- Biosecurity: Dispose of used packaging, gloves, and absorbents as regulated medical waste.
- Audit Compliance: All records must be available for internal and external audits.

Quality Records

- Sample labeling: Patient ID, date/time of sampling, sample type (skin swab).
- Rejection criteria:
  - Sample desiccation or leakage
  - Illegible or missing labels
  - Storage temperature not meeting protocol requirements
- Sample Invalidity Criteria
  - Major failure: No cryopreservation in liquid nitrogen/dry ice within 15 minutes
  - Moderate failure: Inadequate sample material (swab with no visible collection)
  - Minor failure: Sequencing quality not qualified (validation through sequencing required, aside from Major failure and Moderate failure cases).

# **Data Collection and Processing SOPs**

### **SOP12: Standardized Collection Procedures for Basic Demographics and Clinical Data**

Scope: Principal Investigators (PIs) at clinical centers, Sub-Investigators (Sub-Is), sampling staff, sample quality control staff, central laboratory staff.

Purpose: This SOP is established to standardize the collection of demographic data, baseline clinical information, and dynamic clinical records of critically burned patients, ensuring data completeness, accuracy, and compliance with the study protocol.

Data Collection Content

- A new patient record should be created in the standardized information collection system (Electronic Data Capture, EDC). The system collects clinical information, including personal information, injury information, treatment information, additional data, and prognosis information.
- The SOP specifies variable types (e.g., continuous variable formats, categorical levels) and upload formats (e.g., auxiliary examination data requires only photographs of reports).
- At the end of each patient’s study cycle, full multidimensional clinical data must be collected.

Standardized Information Collection System (EDC):

- Login URL: http://8.152.99.39/mryAzfSVtu.php/index/login
- Supports PC client and mobile client.
- Each sub-center has its own account and may only manage data from its own study site.
- Only administrator accounts may manage multi-center data.

Required Materials of Standardized Collection Procedures for Basic Demographics and Clinical Data

- Recording Tools: Standardized information collection system (EDC), eCRF, HIS access, paper-based "Biological Sample Linkage Registration Form".
- Laboratory Resources: Central lab booking system, sterile blood collection tubes (EDTA/Heparin/Clotting), transport boxes, dry ice.

Operational Procedures

Patient Screening

- Check inclusion/exclusion criteria before enrollment.
- Obtain informed consent from patient or legal guardian.

Time Points for Data and Sample Collection

- Time Point 1 (Day 1–2 ± 1 after burn): Collect 6 microbiome sample types (burned/unburned skin swabs, intestinal samples, oral swabs). Record full clinical information.
- Time Point 2 (Day 3 ± 1): Collect intestinal and oral swabs. Record daily clinical information.
- Time Point 3 (Day 7 ± 1): Same as above.
- Time Point 4 (Day 14 ± 1): Same as above.
- Time Point 5 (Day 28 ± 1): Same as above.
- Time Point 6 (Sepsis occurrence, 3 consecutive days): Same as above.
- Time Point 7 (Day of wound healing): Collect 6 microbiome sample types. Record clinical data.
- Time Point 8 (3 months ± 7 days): Collect 6 microbiome sample types. Record clinical data.
- Time Point 9 (6 months ± 7 days): Collect 6 microbiome sample types. Record clinical data.
- Time Point 10 (12 months ± 7 days): Collect 6 microbiome sample types. Record clinical data. Study terminates at 12 months or upon patient death.

**Data Recording Standards of Standardized Collection Procedures for Basic Demographics and Clinical Data**

- **Patient ID: A unique research code must be used (**e.g., BIOME-CHYY01 for BIOME The First Affiliated Hospital of Naval Medical University No.01**). Do not use the patient's name or hospital ID.**
- **Time Windows: A tolerance of ± 24 hours is allowed for scheduled laboratory examinations.**
- **Missing Data: Any missing data must be recorded as "Not Tested," accompanied by the reason.**
- **AE/SAE:** AE and SAE **must be graded according to NCI-CTCAE v4.03 and reported to the Ethics Committee within 48 hours.**

Clinical data entry into standardized information collection system: At enrollment, a new patient record will be created in the standardized electronic data collection system (EDC). Data collection must strictly follow the SOPs, which specifies variable formats (e.g., continuous variable units, categorical variable levels) and upload standards (e.g., the images of sampling sites be uploaded as photos).The clinical dataset includes five domains:

Personal Information (baseline only)

- Past health status
- Gender
- Age
- Height
- Weight

Burn Injury Information (baseline and major clinical milestones)

- Cause of burn injury (e.g., flame, scald, chemical, electrical)
- Total body surface area (TBSA) affected
- Anatomical locations of burn injury
- Presence of concomitant injuries
- Photographs documenting overall injury status at admission

Treatment Information (updated at relevant timepoints)

- Pre-hospital emergency care (e.g., airway management, fluid resuscitation)
- Resuscitation details (e.g., shock fluid volume and type)
- Wound management (initial debridement, dressing)
- Infection prevention measures (antibiotics, antiseptic dressings)
- Nutritional support interventions
- Complication prevention strategies (e.g., DVT prophylaxis, organ support)

Other Information (collected as available)

- Treating institution (hospital name/center code)
- Administrative division (geographical/urban–rural category)
- Socioeconomic data (if available, e.g., insurance type, income bracket)

Prognosis and Follow-up Information (longitudinal; collected at specific endpoints)

- Sepsis occurrence (per ABA definition)
- Sepsis-related mortality
- All-cause mortality
- Wound-healing time (days to complete closure at sampling sites)
- Vancouver Scar Scale (VSS) total and subscores (pigmentation, vascularity, pliability, height, pain, pruritus; only after wound healing is achieved)

Workflow of Standardized Information Collection System

- Step 1: Enter login username and password.
- Step 2: Access dashboard and create new patient record.
- Step 3: Add baseline patient data and assign unique study ID.
- Step 4: Upload photographs of reports (must include dates).

Quality Control

- Double-check system: Initial entry by sampling physician → verification within 24h by study physician.
- Logical validation: Automatic alerts by eCRF.
- Traceability: All data must match HIS or source records; discrepancies require written justification.

### **SOP13: Standardized Collection Procedures for Laboratory, Imagological Examination and Scoring Forms**

Scope: Principal Investigators (PIs) at clinical centers, Sub-Investigators (Sub-Is), sampling staff, sample quality control staff, central laboratory staff.

Purpose: To standardize the procedures for clinical diagnosis, laboratory examinations, imagological assessments, and the administration of scoring systems including Sequential Organ Failure Assessment (SOFA), VSS, ABA Sepsis Definition, and SF-36. This SOP ensures that data collection is accurate, consistent, and aligned with study protocols.

(2) Key Definitions and Standards

Sepsis Diagnosis: ABA Sepsis Definition (Burn-Specific) [1]

Criteria (at least 3 required + documented infection evidence):

- Temperature ≥ 39°C or ≤ 36.5°C
- Progressive tachycardia:

- Adults > 110 bpm

- Children > 2 SD above age-specific norms (85% max HR)

- Progressive tachypnea:

- Adults > 25 bpm not ventilated or > 12 L/min ventilated

- Children > 2 SD above age-specific norms (85% max RR)

- Thrombocytopenia (not within first 3 days post-resuscitation):

- Adults < 100,000/μL

- Children < 2 SD below age-specific norms

- Hyperglycemia (without pre-existing diabetes mellitus):

- Plasma glucose > 200 mg/dL

- Insulin resistance: > 7 units/h IV insulin (adults) or > 25% increase in 24h

- Inability to continue enteral feeding > 24h (abdominal distension, feeding intolerance, uncontrollable diarrhea)
- Documented infection required (culture positive, pathologic tissue source, or clinical response to antimicrobials).

Septic shock diagnostic criteria must meet all three of the following:

- Diagnosis of sepsis established;
- Need for vasopressor therapy to maintain mean arterial pressure (MAP) > 65 mmHg after adequate fluid resuscitation;
  Blood lactate concentration > 2 mmol/L.

For patients with fever and organ dysfunction or unexplained organ dysfunction, sepsis should be considered. Score of quick SOFA (qSOFA) > 2 can be used for rapid bedside screening of suspected sepsis patients.

Sequential Organ Failure Assessment (SOFA) Score

1. Respiration

*Scores are based on fraction of inspired oxygen / partial pressure of arterial oxygen (PaO₂/FiO₂).

- Score 0: ≥ 400 mmHg (53.3 kPa)
- Score 1: < 400 mmHg (53.3 kPa)
- Score 2: < 300 mmHg (40 kPa)
- Score 3: < 200 mmHg (26.7 kPa) with respiratory support
- Score 4: < 100 mmHg (13.3 kPa) with respiratory support

2. Coagulation (Platelets)

- Score 0: ≥ 150 × 10³/ mcL (≥ 150 × 10⁹/L)
- Score 1: < 150 × 10³/ mcL
- Score 2: < 100 × 10³/ mcL
- Score 3: < 50 × 10³/ mcL
- Score 4: < 20 × 10³/ mcL

3. Liver (Bilirubin)

- Score 0: < 1.2 mg/dL (20 µmol/L)
- Score 1: 1.2–1.9 mg/dL (20 – 32 µmol/L)
- Score 2: 2.0 – 5.9 mg/dL (33 – 101 µmol/L)
- Score 3: 6.0 – 11.9 mg/dL (102 – 204 µmol/L)
- Score 4: > 12.0 mg/dL (> 204 µmol/L)

4. Cardiovascular

*Scores are based on mean arterial pressure (MAP) or the requirement for vasopressors/inotropes (at a stable dose for ≥ 1 hour).

- Score 0: MAP ≥ 70 mmHg
- Score 1: MAP < 70 mmHg
- Score 2: Administration of dopamine at < 5 µg/kg/min or any dose of dobutamine.
- Score 3: Administration of dopamine at 5.1 – 15 µg/kg/min, epinephrine at ≤ 0.1 µg/kg/min, or norepinephrine at ≤ 0.1 µg/kg/min.
- Score 4: Administration of dopamine at > 15 µg/kg/min, epinephrine at > 0.1 µg/kg/min, or norepinephrine at > 0.1 µg/kg/min.

5. Central Nervous System (Glasgow Coma Scale (GCS))

- Score 0: GCS score of 15
- Score 1: GCS score of 13 – 14
- Score 2: GCS score of 10 – 12
- Score 3: GCS score of 6 – 9
- Score 4: GCS score of < 6

6. Renal Function

*Scoring is based on either serum creatinine level or daily urine output.

- Score 0: Creatinine < 1.2 mg/dL (110 µmol/L)
- Score 1: Creatinine 1.2 – 1.9 mg/dL (110 – 170 µmol/L)
- Score 2: Creatinine 2.0 – 3.4 mg/dL (171 – 299 µmol/L)
- Score 3: Creatinine 3.5 – 4.9 mg/dL (300 – 440 µmol/L) or urine output < 500 mL/day
- Score 4: Creatinine > 5.0 mg/dL (> 440 µmol/L) or urine output < 200 mL/day

Vancouver Scar Scale (VSS)

All baseline scores must be compared with unaffected skin areas, with scar tissue pressed using a specialized glass plate for 2 seconds before assessment.

Pigmentation

- Similar to adjacent skin
- Hypopigmentation
- Mixed pigmentation
- Hyperpigmentation

Vascularity

- Similar to normal skin
- Pink, slightly increased blood supply
- Red, moderately increased blood supply
- Purple or deep red, markedly increased blood supply

Height/Thickness

- Flat, same as surrounding skin
- Elevated ≤ 2 mm
- Elevated > 2 mm but ≤ 5 mm
- Elevated > 5 mm

Pliability

- Normal
- Supple (minimal resistance, easily deformable)
- Yielding (flexible under pressure)
- Firm (resistant, rigid, non-elastic)
- Banding (cord-like contracture during extension)
- Contracture (permanent shortening causing deformity)

SF-36 (Short Form Health Survey)

- Physical Functioning (PF) - 10 Items

*Items 1-10: "Does your health now limit you in these activities?" (Response: 1=Yes, limited a lot; 2=Yes, limited a little; 3=No, not limited at all)

1. Vigorous activities, such as running, lifting heavy objects, participating in strenuous sports.

2. Moderate activities, such as moving a table, pushing a vacuum cleaner, bowling, or playing golf.

3. Lifting or carrying groceries.

4. Climbing several flights of stairs.

5. Climbing one flight of stairs.

6. Bending, kneeling, or stooping.

7. Walking more than a mile.

8. Walking several blocks.

9. Walking one block.

10. Bathing or dressing yourself.

- Physical Health (RP) - 4 Items

*Items 11-14: "During the past 4 weeks, have you had any of the following problems with your work or other regular daily activities as a result of your physical health?" (Response: 1=Yes; 2=No)

11. Cut down on the amount of time you spent on work or other activities.

12. Accomplished less than you would like.

13. Were limited in the kind of work or other activities.

14. Had difficulty performing the work or other activities (for example, it took extra effort).

- Emotional Problems (RE) - 3 Items

*Items 15-17: "During the past 4 weeks, have you had any of the following problems with your work or other regular daily activities as a result of any emotional problems (such as feeling depressed or anxious)?" (Response: 1=Yes; 2=No)

15. Cut down on the amount of time you spent on work or other activities.

16. Accomplished less than you would like.

17. Didn't do work or other activities as carefully as usual.

- Vitality (VT) - 4 Items

*Items 18-21: "How much of the time during the past 4 weeks..." (Response for all: 1=All of the time; 2=Most of the time; 3=A good bit of the time; 4=Some of the time; 5=A little of the time; 6=None of the time). Items 20 and 21 are reverse-scored.

18. Did you feel full of life?

19. Did you have a lot of energy?

20. Did you feel worn out? (Reverse-scored)

21. Did you feel tired? (Reverse-scored)

- Mental Health (MH) - 5 Items

*Items 22-26: "How much of the time during the past 4 weeks..." (Response for all: 1=All of the time to 6=None of the time). Items 22, 23, and 25 are reverse-scored.

22. Have you been a very nervous person? (Reverse-scored)

23. Have you felt so down in the dumps that nothing could cheer you up? (Reverse-scored)

24. Have you felt calm and peaceful?

25. Have you felt downhearted and blue? (Reverse-scored)

26. Have you been a happy person?

- Social Functioning (SF) - 2 Items

27. "During the past 4 weeks, to what extent has your physical health or emotional problems interfered with your normal social activities with family, friends, neighbors, or groups?" (Response: 1=Not at all; 2=Slightly; 3=Moderately; 4=Quite a bit; 5=Extremely)

28. "During the past 4 weeks, how much of the time has your physical health or emotional problems interfered with your social activities (like visiting with friends, relatives, etc.)?" (Response: 1=All of the time; 2=Most of the time; 3=Some of the time; 4=A little of the time; 5=None of the time)

- Bodily Pain (BP) - 2 Items

29. "How much bodily pain have you had during the past 4 weeks?" (Response: 1=None; 2=Very mild; 3=Mild; 4=Moderate; 5=Severe; 6=Very severe)

30. "During the past 4 weeks, how much did pain interfere with your normal work (including both work outside the home and housework)?" (Response: 1=Not at all; 2=A little bit; 3=Moderately; 4=Quite a bit; 5=Extremely)

- General Health (GH) - 5 Items

31. "In general, would you say your health is:" (Response: 1=Excellent; 2=Very good; 3=Good; 4=Fair; 5=Poor)

32. "I seem to get sick a little easier than other people." (Response: 1=Definitely true; 2=Mostly true; 3=Don't know; 4=Mostly false; 5=Definitely false) (Reverse-scored)

33. "I am as healthy as anybody I know." (Response: 1=Definitely true to 5=Definitely false) (Reverse-scored)

34. "My health is excellent." (Response: 1=Definitely true to 5=Definitely false) (Reverse-scored)

- Health Transition (HT) - 1 Item

35. "Compared to one year ago, how would you rate your health in general now?" (Response: 1=Much better now than one year ago; 2=Somewhat better now than one year ago; 3=About the same; 4=Somewhat worse now than one year ago; 5=Much worse now than one year ago)

**Unified Assessment Timeline**

- **SOFA Score: This organ dysfunction assessment is performed at all scheduled visits (Days 1**–**2, 3, 7, 14, 28, 3m, 6m, 12m) and during event-driven visits for sepsis and wound healing.**
- **ABA Sepsis Criteria: Screening for sepsis using ABA criteria is conducted concurrently with the SOFA score at all scheduled and event-driven visits.**
- **Vancouver Scar Scale (VSS): Scar assessment is performed starting at the wound healing evaluation time point and then at all subsequent long-term follow-ups (Months 3, 6, and 12).**
- **SF-36 Health Survey: The** quality-of-life **questionnaire is administered during the long-term follow-up phase at Months 3, 6, and 12.**

eCRF Recording Rules

- Laboratory data: directly imported from LIS (Laboratory Information System) (manual entry prohibited). Non-LIS centers require double verification of original reports.
- SOFA scoring: record each system’s value (e.g., "Respiration: PaO₂/FiO₂ = 201 → 3 points").
- Sepsis event marking: lock "Sepsis Event" module in eCRF within 24 hours of diagnosis.
- Scoring Form Collection Protocols

### **SOP14: Follow-up Plan Execution and Data Collection Procedures**

. Scope: Principal Investigators (PIs) at clinical centers, Sub-Investigators (Sub-Is), sampling staff, sample quality control staff, central laboratory staff.

Purpose: This SOP is established to standardize the follow-up execution and multimodal data collection procedures for critically burned patients, ensuring compliance with the study timeline and data completeness across all clinical centers.

**In-hospital period:**

- **Day 1 – 2, 3, 7, 14, 28: Vital signs and laboratory parameters** are **collected bedside with a tolerance window of ±** **1 day.**
- **Wound healing assessment: Vital signs and laboratory parameters** are **collected bedside with a tolerance window of ±** **1 day.**
- **Sepsis Event (T0): Additional blood culture and metagenomic samples** are **collected using an emergency sampling kit.**

**Post-discharge period:**

- **3, 6, and 12 months:** SF-36 scale and VSS scar scores are collected via outpatient visits and electronic questionnaires. Scar tissue sampling and quality-of-life assessments are conducted using a home follow-up kit with video guidance, with a permitted tolerance window of ± 7 days.

Post-discharge Follow-up Strategy

- Local patient: Follow-up is conducted via outpatient visit with on-site sampling, and quality control requires that scar photos include a measurement scale and a color card.
- Non-local patient: Follow-up utilizes a home kit with an illustrated guide, and quality control requires continuous temperature tracking during transport, maintained above -60℃.
- High-risk for loss to follow-up: Follow-up employs a triple-contact strategy involving the patient, family, and attending physician, and quality control mandates a minimum of 5 call attempts.

Multi-source Data Integration

- Laboratory reports: Input through direct LIS–eCRF linkage with auto-flagging for out-of-range values.
- Scar photos: Uploaded via encrypted cloud platform with AI validation for scale calibration.
- Patient self-reports: Require e-signature verification and re-entry if completed in under 2 minutes.

Handling Time Window Deviations

- Sampling delay ≤ 48 h: Corrected by normal data entry with the reason for delay documented.
- Sampling delay > 48 h: Corrected by marking the event as a “Protocol Deviation” and discontinuing sampling for that specific time point.
- Missing questionnaire: Corrected by conducting a phone-based supplement within 72 hours, which requires recorded confirmation.

Sample–Data Matching

Each sample tube must be labeled with a unique sample ID, using the format:

- BIOME–CenterID–PatientSeq–SampleType–TimePoint–Replicate

Examples:

- BIOME-CHYY01-NS-Limb-D1-REP1 — Normal skin (limb), Day 1, replicate 1
- BIOME-CHYY01-BW-Trunk-D1-REP1 — Burn wound (trunk), Day 1, replicate 1
- BIOME-CHYY01-SS-D1-REP1 — Stool sample, Day 1, replicate 1
- BIOME-CHYY01-AS-D1-REP1 — Anal swab, Day 1, replicate 1
- BIOME-CHYY01-OS-D1-REP1 — Oral swab, Day 1, replicate 1

eCRF requirements: Each entry must include:

- Sample ID
- Collection time
- Operator signature
- Temperature log

Data Quality Traceability

- All sample and clinical data entries must be traceable to their original source documents (laboratory report, case record, or electronic signature).
- Discrepancies between eCRF and source data must be documented in a “Data Clarification Form (DCF)” within 72 hours.
- Data lock can only occur after reconciliation between clinical and laboratory datasets.

# **Statistical Analysis Plan (SAP)**

### **SAP01: Data Cleaning and Standardized Processing Procedures**

Scope: Principal Investigators (PIs) at clinical centers, Sub-Investigators (Sub-Is), sampling staff, sample quality control staff, central laboratory staff.

Purpose: To establish an automated data-cleaning pipeline that transforms raw clinical and metagenomic data into analysis-ready datasets in compliance with CDISC (Clinical Data Interchange Standards Consortium) and MIxS (Minimum Information about any Sequence) standards. This ensures data integrity, interoperability, and readiness for downstream biostatistical and bioinformatics analyses.

Data Flow Architecture

Raw Data Source → Data Receipt Validation → Field-Level Cleaning → Logical Consistency Check → Microbial Data Standardization → Analysis-Ready Dataset Generation

Data Cleaning Rule Library

Clinical Data Cleaning Rules

- Numeric fields: For out-of-range physiological values (e.g., TBSA > 100%), replace the value with null and flag it with code QM04 (Query – Logical Error).
- Time-series fields: In cases of time inversion (e.g., recorded sampling time later than freezing time), automatically correct the time to 10 minutes prior to the sample receipt time.
- Categorical fields: When non-standard terminology is used, map the entry to a standardized taxonomy term (e.g., NCBI:208964_Pseudomonas).

Rules Metagenomic Data Cleaning Rules

- Species abundance: When contamination is detected (human DNA > 95%), the sample is discarded and the re-sampling workflow is triggered.
- Gene functional annotation: Unmatched KEGG Orthology terms are flagged as "Unknown" and retained for independent exploratory analysis.
- Sampling–sequencing interval: If the interval exceeds 30 days, the data is downgraded to the exploratory analysis dataset.

Microbial Taxonomy Standardization

\# Example: 16S rRNA taxonomy standardization script (Python code)

def standardize_taxonomy(raw_name):

ncbi_dict = load_ncbi_reference() # Load NCBI taxonomy reference

if raw_name in ncbi_dict:

return ncbi_dict [raw_name] # Return standardized taxon ID

else:

return blast_nearest_match(raw_name) # Nearest-match BLAST mapping

Clinical Terminology Mapping

- Wound infection: Standardized as code AE10027460 following CTCAE v4.03 encoding.
- Third-degree burn: Standardized as code CM2001231 through mapping based on ICD-11.
- Vancomycin: Standardized as code C254789 according to the WHO Drug Dictionary

Quality Control Metrics for Cleaning Process Monitoring

- Field missing rate: If the rate exceeds 5%, it triggers Source Data Verification (SDV).
- Microbial naming failure rate: If the rate exceeds 1%, it necessitates an update to the NCBI reference library.
- Time window alignment deviation: If the deviation exceeds 48 hours, the data is flagged as "Offset" with no correction applied.

Analysis-Ready Dataset Structure

- ADSL (Subject-Level Dataset): Contains core variables such as demographics, grouping, and treatment exposure for use in baseline characteristic analysis.
- ADLB (Laboratory Dataset): Includes standardized laboratory values, units, and timepoints to support longitudinal trend analysis.
- ADMG (Metagenomic Dataset): Provides a species abundance matrix (rows = samples, columns = NCBI Taxa IDs) applicable for microbiome composition and evolution analysis.

Output Dataset Validation

- Clinical dataset: Validated using CDISC Validator v2.0
- Metagenomic dataset: Must conform to MIxS (Minimum Information about any Sequence) requirements

Data Dictionary Requirements

Each variable must include:

- Variable Name (≤ 8 characters)
- Label (bilingual descriptive field)
- Type (numeric / character / date)
- Unit (standardized to SI units)
- Missing Value Codes (.N = Not available, .L = Below limit, .D = Discrepancy)

Microbial Naming Reference Mapping

- Pseudomonas aeruginosa: Standardized as NCBI Species ID 208964.
- Methicillin-resistant Staphylococcus aureus: Standardized as NCBI Species ID 1280, with subspecies identification annotated.

### **SAP02: Microbiome Sequencing Data Processing and Quality Control Procedures**

Scope: Principal Investigators (PIs) at clinical centers, Sub-Investigators (Sub-Is), sampling staff, sample quality control staff, central laboratory staff.

Purpose: To standardize the workflow and quality control (QC) criteria for shotgun metagenomic sequencing data, ensuring compliance with MIxS (Minimum Information about any Sequence) standards and alignment with the study’s biological hypothesis testing requirements.

Workflow Overview

Input Data Standards

- Raw sequencing data: Requires a Q30 score of at least 80% and a minimum of 10 million reads per sample, with data being rejected if the Q30 score falls below 70% or reads are fewer than 5 million.
- Sample metadata: Must contain complete MIxS core fields, and samples are rejected if key fields (e.g., sampling site) are missing.
- Host contamination control: For skin samples, human DNA must not exceed 40%, and samples are rejected if contamination surpasses 60%.

Standardized Analytical Workflow

Data Input → Raw FASTQ → Quality Trimming → Host Sequence Removal → Metagenomic Assembly → Gene Prediction → Taxonomic/Functional Annotation → Abundance Matrix Generation

Key Analytical Parameters

- Quality trimming: Performed using Trimmomatic with parameters SLIDINGWINDOW 4:20 and MINLEN 50, requiring at least 90% of reads to be retained.
- Host sequence removal: Conducted with KneadData(hg38 reference), requiring a minimum human sequence removal rate of 99.5%.
- Metagenomic assembly: Executed using MEGAHIT (kmin = 21, kmax = 141, kstep = 10), requiring an assembly N50 of at least 5 kb and a minimum of 50,000 contigs.
- Gene prediction: Performed with Prodigal using the meta mode (-p meta), requiring the prediction of at least 500,000 genes per sample.
- Taxonomic annotation: Conducted using Kraken2 with Bracken (standardDB), requiring a minimum confidence score of 0.8 for all assignments.

Process Monitoring Indicators

- Sequencing: Monitors average read length and Q30 ratio; triggers re-sequencing if Q30 falls below 75%.
- Assembly: Tracks N50 and maximum contig length; requires parameter adjustment and reassembly if N50 is under 3 kb.
- Annotation: Measures the proportion of unannotated genes; prompts a reference database update if the proportion exceeds 30%.

Output Data Specifications

Core Output Matrices

- Species abundance: Formatted as BIOM 2.1, with rows representing NCBI Species IDs and columns representing Sample IDs.
- Functional pathways: Formatted as TSV, with rows representing KEGG Orthology terms and columns representing Sample IDs.
- Antimicrobial resistance genes: Formatted as TSV, with rows representing CARD identifiers and columns representing Sample IDs.

Metadata Association Requirements

Each output matrix must include:

- Sample ID (matched with the clinical dataset ADSL)
- Sampling timepoint
- Sequencing depth (normalized to per million reads)

Exception Handling Procedures

- High host contamination: Handled by re-running host decontamination; if unsuccessful, the sample is downgraded to 16S analysis, and the contamination rate with corrective actions is documented.
- Low assembly quality: Addressed by switching to an alternative assembly algorithm (e.g., IDBA-UD or SPAdes), and assembly metrics from different versions are saved for comparison.
- Annotation consistency < 60%: Requires manual review followed by BLASTP validation, and a "Disputed Species Watchlist" is established based on the findings.

Metagenomic Sequencing QC Scope

Quality control (QC) is conducted across multiple stages:

- Compliance of sample preservation and transportation
- DNA quality inspection
- Library quality inspection
- Raw data statistics and sequencing QC

Metagenomic Sequencing QC Methods and Standards

Compliance of Sample Preservation and Transportation

- Immediate Handling: Following collection, cryopreservation must be performed in liquid nitrogen or on dry ice within 15 minutes or less.
- Storage Conditions: Samples must be stored at -80℃. The use of cryoprotectants is strictly prohibited.
- Transportation: Samples must be transported with dry ice according to SOP05. Shipment records are to be submitted to the shared group. Upon receipt, the central laboratory must perform quality control (QC) checks and provide feedback within 24 hours.

During sample submission, an electronic sample submission form must be completed. At receipt, the central laboratory verifies sample condition and remaining dry ice.

- Samples received frozen with dry ice remaining are considered compliant.
- Samples that have thawed upon receipt are considered noncompliant.

DNA Quality Control (DNA QC)

After sample receipt, DNA extraction and quality inspection must be performed.
DNA samples must meet the following minimum requirements:

- Concentration ≥ 20 ng/μL
- Total yield ≥ 500 ng
- No degradation, RNA contamination, or discoloration/turbidity

DNA QC Methods:

- Quantification: Qubit fluorometric quantification
- Purity & Integrity: Agarose gel electrophoresis (0.8% gel, 120 V)

Sample Grading Criteria

- Grade I: No remarks; yield sufficient for at least 2 library constructions. Result: Qualified.
- Grade II: Contains a minor remark (A, C, or H) or yield supports only one library. Result: Qualified (requires preprocessing).
- Grade III: Contains remark F or I, or has insufficient yield (B); considered a borderline case. Result: Possible (requires discussion).
- Grade IV: Contains a serious remark (D, E, or G) indicating degradation or contamination. Result: Possible (no quality guarantee).
- Grade V: Contains remark J, indicating critically insufficient yield. Result: Not buildable.

Remark Codes

- Code A: Mild RNA contamination detected.
- Code B: Insufficient total yield for processing.
- Code C: Insufficient concentration for standard procedures.
- Code D: Severe degradation indicated by a weak gDNA band.
- Code E: Heavy RNA contamination present.
- Code F: Small fragment contamination observed.
- Code G: No target band detected.
- Code H: Slight discoloration, turbidity, or precipitate noted.
- Code I: Severe turbidity, viscosity, or dark coloration present.
- Code J: Extremely low yield; library construction not feasible.

Acceptance Criteria for DNBSEQ-T7 Sequencing:

- Total library yield ≥ 60 ng
- Concentration ≥ 2.0 ng/μL
- Volume ≥ 15 μL
- Main fragment size: 480–520 bp

Qualified libraries are sequenced on the DNBSEQ-T7 platform (PE150 mode).

Raw Data Statistics and Sequencing QC

Raw sequencing data (Raw Data) refers to the direct output from the sequencing platform prior to any QC processing.

Additional analyses may include:

- Base composition (A/T/G/C) distribution plots
- Read quality and error rate distribution

Further QC steps are recommended:

- Primary QC (Clean Data): remove adapters, duplicates, and low-quality reads.

Secondary QC: align Clean Data to the host reference genome to assess host contamination. Summary of QC Methods and Standards

- Sample preservation & transport: QC is performed by adhering to standard SOPs for freezing and dry ice transport, with the required standard being that samples must remain frozen with dry ice upon receipt, and responsibility lies with both the sampler and central lab receiver.
- DNA QC: QC is conducted via Qubit quantification and agarose gel integrity checks, with standards requiring a concentration ≥ 20 ng/μL, a total amount ≥ 500 ng, and no signs of degradation or contamination.
- Library QC: QC involves Qubit quantification and electrophoresis for fragment sizing, requiring a library yield ≥ 60 ng, concentration ≥ 2.3 ng/μL, volume ≥ 15 μL, and a fragment size between 480 – 520 bp.
- Raw sequencing QC: QC is performed on the raw data, requiring Q20 ≥ 90% and Q30 ≥ 85%.
- Clean data QC: QC is performed on the post-filtered reads, though no unified standard is specified.
- Secondary QC: QC involves analyzing host-mapped reads, with no unified standard as it varies by sample type.

Standard Pipeline Configuration (Snakemake Example)

\# Core Snakemake rules

rule all:

input: expand("results/.species.tsv", sample=SAMPLES)

rule qc:

input: "raw/.fastq"

output: "trimmed/.fq"

shell: "trimmomatic SE -threads 8 {input} {output} SLIDINGWINDOW:4:20 MINLEN:50"

rule remove_host:

input: "trimmed/{sample}.fq"

output: "clean/{sample}.fq"

shell: "kneaddata -i {input} -o clean/ --reference-db hg38"

rule assembly:

input: "clean/{sample}.fq"

output: "assembly/{sample}/contigs.fa"

shell: "megahit -r {input} -o {wildcards.sample} --k-min 21 --k-max 141 --k-step 10"

**Mandatory QC Report Metrics**

- **Effective data ratio: Calculated as Clean reads divided by Raw reads, with an acceptance value of ≥ 85%.**
- **Taxonomic annotation coverage: Calculated as Annotated reads divided by Clean reads, with an acceptance value of ≥ 60%.**
- **α-Diversity stability: Measured using the Intraclass correlation coefficient (ICC) of the Shannon index, with an acceptance value of ≥ 0.8.**

### **SAP03: Implementation of Statistical Analysis Plan**

Scope: Principal Investigators (PIs) at clinical centers, Sub-Investigators (Sub-Is), sampling staff, sample quality control staff, central laboratory staff, and Data Analysts

Purpose: To standardize the execution process from analysis-ready datasets to finalized statistical reports, ensuring that clinical and metagenomic statistical analyses align with the prespecified hypotheses, regulatory compliance, and reproducibility requirements of the study protocol.

Analytical Framework

Clinical Dataset → Hypothesis Testing → Metagenomic Dataset → Microbial Network Analysis → Integrated Modeling → Statistical Reporting

Library construction and metagenomic sequencing

Metagenomic libraries were constructed following the manufacturer’s introductions (Illumina, San Diego, CA, USA), and index codes were added to attribute the sequences of each sample. The clustering of the index-coded samples was performed on a cBot Cluster Generation System according to the manufacturer’s instructions. After cluster generation, the metagenomic library preparations were sequenced on a Novaseq 6000 platform adopting a 150 bp paired-end sequencing strategy by Berry Genomics Company (http://www.berrygenomics.com/. Beijing, China). Basically, the libraries were prepared following these steps: DNA fragmentation by sonication, end-polishing of the DNA fragments, A-tailing and ligation with the full-length adapters for Illumina sequencing, PCR amplification, and purification of PCR products (AMPure XP bead system). The libraries were analyzed for size distribution using an Agilent 2100 Bioanalyzer and were quantified using real-time PCR.

Sequence quality checking and filtering

To avoid reads with artificial bias (i.e., low-quality paired reads, which primarily result from base-calling duplicates and adaptor contamination), we removed the following types of reads:

- reads with 3nt unidentified nucleotides (N);
- reads aligned to the adaptor;
- reads with ≥ 20% bases having phred quality ≤ 5.

The reads mapped to the host genomic DNA by BWA MEM (v0.7.17-r1188) [35] were filtered out.

Metagenome assembly

The filtered reads were processed by MEGAHIT (v1.1.3) [36] using the options ‘--k-min 21 --k-max 141 --k-step 12 --min-count 2’ for metagenome assembly. The sequence data of each sample were assembled individually. The contigs with length more than 500 bp were retained for gene prediction.

Construction of the gene catalog

The contigs assembled above were used for gene prediction by Blast Prodigal (v2.6.3) [37] software with default parameters. After removing the genes shorter than 100 bp, the remaining gene were clustered at protein level following 95% of protein identity and 90% of coverage using CD-HIT (v4.8.1) [38] using the option ‘-c0.95-n8-|100-aS0.9-d0’. The clean data of each sample were mapped to the primary nonredundant set of genes by BWA MEM and reads with mapping quality less than 30 were filtered by SAMtools [39]. The relative abundance of genes in the samples wascalculated based on the count of aligned reads, normalized to the gene length and sum of the abundance of allgenes. Based on the taxonomic annotation of the gene catalogue, aligned reads were used to quantify taxonomic abundance profiles within samples. Relative taxonomic abundance was normalized to sequencing depth and the length of the genes originating from the same level of taxonomic classification. Genes with depth more than 2 were retained for further analyses.

Taxonomic and functional annotation of genes

The amino acid sequences of proteins in the catalog were aligned to the Non-Redundant Protein Database (NR) by DIAMOND (v0.9.21.122) [40] with options‘--evalue 1e-10 --max-target-seqs 5’. The genes that could not be aligned in the database were defined as undefined taxa. For those genes that were matched to distinguishable taxonomic groups, the taxonomic classification was determined based on the lowest common ancestor (LCA) algorithms by DIAMOND whose score is at most 10% lower than the best score. The genes that could not be classified to any taxa were defined as unclassified taxa.

Bray–Curtis dissimilarity values were calculated to assess beta-diversity. The Python package Qimime2 was employed for multivariate statistical analyses, including PCA, PCoA (using Jensen–Shannon divergence), NMDS. While UPGMA was perform by Python packages scipy and skbio, Anosim was performed using R package vegan, corroborated the distinct distribution patterns of bacteria related to this study.

Principal Component Analysis (PCA) is a method for analyzing and simplifying datasets. Through variance decomposition, it reflects the differences among multiple sets of data on a two-dimensional coordinate plot, where the coordinate axes represent the two eigenvalues that maximize the variance explanation. The closer the distance between two samples on the PCA plot, the more similar the species composition of the two samples (Z-score) [41]. Principal Coordinates Analysis (PCoA) is a dimensionality reduction and ordination method similar to PCA. The principle is that if there are data measuring the differences or distances among N samples, this method can be used to find a Cartesian coordinate system, representing the N samples as N points. The squared distances between these points exactly match the original difference data, realizing the quantitative conversion of qualitative data and extracting the most important elements and structures from multidimensional data. Principal Coordinates Analysis enables the classification of multiple samples and displays the differences in species diversity among samples. Based on the species abundances at each taxonomic level, Bray-Curtis and Euclidean distances are calculated respectively, followed by PCoA analysis. The more similar the species composition of the samples, the closer their distances in the dimensionality-reduced plot [41].

Non-Metric Multi-Dimensional Scaling (NMDS) is an ordination method suitable for ecological research. It mainly simplifies research objects (samples or variables) in multi-dimensional space to low-dimensional space for positioning, analysis, and classification, while retaining the original relationships among the objects. The model of NMDS is non-linear, which can better reflect the non-linear structure of ecological data. Similar to PCA or PCoA, the differences between groups or within groups can be observed through the distribution of samples in NMDS [42]. Unweighted Pair-group Method with Arithmetic Mean (UPGMA) is a commonly used cluster analysis method for hierarchical clustering of samples. Based on the Bray-Curtis and Euclidean distance matrices respectively, hierarchical clustering of samples was performed using the UPGMA method via R language tools to determine the similarity of species composition among different samples. The shorter the branch length, the more similar the species composition of the two samples [43].

Analysis of Similarities (ANOSIM) is a non-parametric statistical test method used to analyze the similarity between groups of multidimensional data. Based on the Bray-Curtis and Jaccard distance algorithms respectively, it examines whether the differences between all groups of species abundances at different taxonomic levels (kingdom, phylum, class, order, family, genus, species) are significantly greater than the differences within groups, thereby determining the validity of the grouping [42].

LEfSe (Linear Discriminant Analysis (LDA) Effect Size) analysis is mainly used to identify species with statistical differences between different groups. It detects differential species among groups through the Kruskal-Wallis rank sum test, performs dimensionality reduction via LDA, and evaluates the impact size of differential features (LDA score) [44]. Spearman correlations were performed between the main genera contributing to clustering and all other genera. Genus networks were visualized using the Cytoscape platform by transforming Spearman correlations into links [45].

The KEGG Kyoto Encyclopedia of Genes and Genomes) annotation results were extracted with KOBAS (v3) [46] software with default parameters. The eggnog (Evolutionary Genealogy of Genes: Non-supervised Orthologous Groups) was annotated by aligning genes to eggNOG 4.5 [47] database using DIAMOND software, and CAZymes (carbohydrate-active enzymes) were annotated by aligning genes to CAZy (Carbohydrate-Active Enzymes Database) with hmmscan program in HMMER (v3.1b2) [48]. ARGs (Antibiotic resistance genes) were annotated by alignment against the Comprehensive Antibiotic Resistance Database (CARD) using RGI (v5.1.1) [49]. The functional annotation of genes was annotated by aligning genes to the Swiss-Prot database using DIAMOND. The GO (Gene Ontology) accessions were linked based on the results of Swiss-Prot annotation. The virulent protein sequences were identified by annotation with the Virulence Factor Database (VFDB) [50] using DIAMOND. The PHI (Pathogen Host Interactions) genes were annotated by aligning genes to the Pathogen Host Interactions Database (PHI-Base) [51] using DIAMOND. The annotation of protein families was extracted with hmmscan program in HMMER (v3.1b2) based on the Pfam database (v33.1) [52]. Cytochrome P450 genes were identified by annotation with the CYPED (Cytochrome P450 Engineering Database) [53]. For the aligned protein sequences, the annotated hit(s) with the highest score was used for the subsequent analysis.

Clinical Statistical Analysis Workflow

Core Analytical Modules

- Baseline Balance: Assessed using the Standardized Mean Difference (SMD) and implemented in R language tools.
- Primary Endpoint (28-day mortality): Analyzed via a Cox Proportional Hazards Model and implemented in R language tools.
- Longitudinal SOFA Score: Modeled using a Linear Mixed Model (LMM) and implemented in R language tools.
- Secondary Infection Rate: Analyzed with a Fine–Gray Competing Risk Model and implemented in R language tools.

Covariate Adjustment Principles

- Mandatory Covariates: Age, TBSA (%)
- Optional Covariates: Variables with |SMD| > 0.1 or p < 0.2 in univariate testing

Standardized Metagenomic Analysis

Core Analytical Pipeline

- **α-Diversity: Analyzed by calculating the Shannon index, requiring an Intraclass Correlation Coefficient (ICC) ≥ 0.8 across replicates to ensure reliability.**
- **β-Diversity: Assessed using Bray–Curtis distance and Principal Coordinate Analysis (PCoA), with a PERMANOVA p‑value < 0.05 required to confirm significant between‑group differences.**
- **Differential Species: Identified via LEfSe (LDA > 3.0), with an FDR‑adjusted q‑value < 0.1.**
- **Functional Pathways: Analyzed using HUMAnN3 and KEGG Modules, requiring pathway coverage ≥ 30% per sample.**
- **Microbial Interaction Network: Constructed with SparCC, requiring |ρ| > 0.3, p < 0.01, and network modularity > 0.4.**

Time-Series Modeling

Microbial trajectory modeling and longitudinal mixed-effect modeling (LMM) are applied for temporal dynamics.

Integrated Clinical–Microbiome Analysis

Omics Association Models

- Microbiome impact on mortality: Analyzed using a multivariate Cox model incorporating microbial biomarkers, resulting in a hazard ratio (HR) forest plot.
- Dynamic association between species and SOFA scores: Examined through a LMM with lagged species abundance, producing time-varying effect curves.
- Relationship between ARGs and antibiotic efficacy: Assessed via logistic regression combined with the CARD database, yielding a resistance–drug sensitivity matrix.

Causal Inference Framework

Mediation Analysis Model:
Mortality ← Microbial species X ← Treatment → mHLA-DR (monocyte Human Leukocyte Antigen DR)

Statistical Implementation: R, calculating ACME (Average Causal Mediation Effect).

Advanced Approaches: Mendelian Randomization and related algorithms.

Multiple Testing Correction Strategy

Layered FDR Control

Species-Level Corrections:

- Genus-level FDR threshold: q < 0.1
- Species-level FDR threshold: q < 0.05

Network Analysis:

- Edge weights adjusted via 1,000 permutation tests.

Software and Version Control

Core Analytical Modules Environment

- **Clinical Statistics: Uses the rocker/tidyverse container image with core dependencies including survival, lme4, and cmprsk.**
- **Metagenomics: Utilizes the biocontainers/microbiome image with core tools QIIME2, LEfSe, and SparCC.**
- **Integrated Analysis: Runs on the jupyter/scipy-notebook image with core packages scikit-learn and statsmodels.**

Reproducibility Assurance

- Code Versioning: Git repository with Semantic Versioning (e.g., v1.2.3-STAT).
- Random Seed Control: Global parameter set.seed(12345).
- Container Freeze: Databases locked within one year after completion of data production, QC, and integration.

Reporting Standards

Required Elements of Statistical Reports

- Baseline Characteristics: Must include a CONSORT flow diagram and a standardized balance table with a SMD of less than 0.1.
- Primary Outcomes: Must present adjusted Hazard Ratios or Risk Ratios (HR/RR) with 95% Confidence Intervals and Kaplan–Meier survival curves.
- Microbial Findings: Requires an LEfSe cladogram and a Principal Coordinates Analysis (PCoA) biplot for visualization.
- Integrated Analysis: Needs a mediation structural equation path diagram and a time-varying association heatmap.

Sensitivity Analyses

Clinical Data: ITT vs. PP comparison.

Metagenomic Data:

- Comparison of annotation databases (Kraken2 vs. MetaPhlAn).
- Sensitivity to host contamination threshold (30% vs. 50%).

SAP Execution Checklist

- Pre-Analysis: Dataset version must be consistent with the SAP appendix, with signature responsibility held by the Statistician.
- Code Execution: Requires random seed locking and container hash verification, with signature responsibility held by the Programmer.
- Report Generation: Mandates that all p-values are labeled with their correction method, with signature responsibility held by the Investigator.

Multiple Testing Correction Hierarchy

- Primary Endpoint (Mortality): Based on a single statistical test with no multiple comparison adjustment applied, using a significance threshold of α = 0.05.
- Secondary Endpoints (Infection): Involves three statistical tests, with the Bonferroni correction applied, resulting in a per-test significance threshold of α = 0.0167.
- Differential Species: Involves testing up to 10,000 features, controlled using the FDR (Benjamini) method, with a significance threshold set at q < 0.1.

### **SAP04: Integration Procedures for Microbiome and Clinical Data Analysis**

Scope: Principal Investigators (PIs) at clinical centers, Sub-Investigators (Sub-Is), sampling staff, sample quality control staff, central laboratory staff.

Purpose: To establish a standardized framework for integrating microbiome and clinical datasets, elucidating the microbial mechanisms underlying sepsis onset and scar formation. This SAP supports hypothesis-driven, reproducible analyses of host–microbiome interactions and provides insights for translational research in severe burn and critical care populations.

Integration Framework

Clinical Data + Microbiome Data → Integrated Matrix → Cohort Stratification → Dynamic Sepsis Modeling → Scar Formation Modeling → Mechanistic Mapping

Data Fusion Standards

**Temporal and Spatial Alignment**

- **Microbial Sampling Time: Aligned to clinical event timestamps with minute-level precision, allowing a tolerance window of ±** **2 hours.**
- **Sepsis Biomarkers: Aligned strictly to the T0 diagnosis time, with zero tolerance permitted.**
- **Scar Assessment: Aligned to the documented day of complete epithelialization, with a tolerance window of ±** **24 hours.**

Batch Effect Correction

All sequencing and metadata variables are batch-corrected using ComBat-seq or equivalent normalization algorithms to harmonize inter-center technical variation.

Multimodal Matrix Construction

Microbial abundance, gene function, and metabolite data are integrated with clinical and immunological variables through standardized normalization (z-score scaling) to form an analytical matrix linking host and microbiome dimensions.

Core Scientific Objective

Through a multicenter, prospective observational study, this project aims to construct temporal and anatomical maps of the microbiome in critically burned patients. It focuses on elucidating the systemic evolution of microbial communities and their associations with clinical outcomes, exploring the interplay between skin barrier reconstruction and microbial ecosystem recovery, and identifying predictive and therapeutic microbial targets.

Multiple Analytical Module 1: Sepsis Dynamics

Early Warning Biomarkers: Developed using LASSO–Cox Regression, resulting in a Microbial Risk Scoring Formula.

Time-Varying Causal Effects: Analyzed via Structural Equation Modeling (SEM), which outputs Path Coefficients quantifying the influence of species on SOFA scores.

Host–Microbe Co-Evolution: Assessed using a Granger Causality Test to identify the Lagged Effect of microbial changes on host mHLA-DR expression.

Treatment Response Prediction: Implemented with XGBoost and SHAP Interpretation to generate the Feature Importance of key microbial species.

**Analytical Module 2: Wound Healing and Scar Formation**

- **Microbial Trajectory Typing: Utilizes Gaussian Mixture Model (GMM) clustering to identify Scar-Associated Ecotypes.**
- **Functional Pathway Enrichment: Applies KEGG MODULE with GSEA to assess collagen synthesis pathway enrichment.**
- **Spatial Co-Localization: Employs STAMP (Spatial Transcriptomics–Microbiome Integration) to generate microbe–fibroblast co-occurrence maps.**
- **Scar Hardness Prediction: Uses Random Forest with 10-fold cross-validation to produce ranked species importance for scar hardness.**

Cross-Cohort Integrated Analysis

Sepsis, Wound Healing and Scar Formation Interaction Effects

- Sepsis-associated microbiota alteration hypothesis: Validated using a Difference-in-Differences (DID) model to compare microbial composition between sepsis and non-sepsis groups.
- Antibiotic perturbation effects hypothesis: Analyzed via a Linear Mixed Model (LMM) to assess how the interaction between antibiotic days and microbial diversity influences wound healing and scar outcomes.

Multi-Omics Mechanistic Mapping

Integrative models combining clinical, transcriptomic, and microbial functional data will be used to visualize causal pathways and microbial–host molecular interactions.

Visualization Standards

Required Visualization Types

- Early Warning Models: Features a Time-dependent Microbial ROC Curve generated using R packages pROC and ggplot2.
- Causal Path Analysis: Visualized via an SEM Path Diagram displaying standardized coefficients, created with the semPlot package.
- Microbial Trajectories: Illustrates dynamics through a 3D PCoA Dynamic Trajectory Plot built with the EMPeror tool.
- Spatial Co-Localization: Combines a Heatmap with Spatial Scatter Overlay using the SpatialExperiment package.

Publication-Grade Output Standards

- Resolution: ≥ 300 dpi
- Format: PDF (vector) or TIFF (raster)
- Color Scheme: ColorBrewer palette (color-blind friendly)

Sensitivity and Validation Analyses

Robustness Testing

- Variable Sequencing Depth: Corrected through rarefaction-based resampling.
- Confounding Factors: Addressed using Inverse Probability of Treatment Weighting (IPTW).
- Time-Dependent Confounding: Modeled and adjusted for with Marginal Structural Models (MSM).

Experimental Validation Design

- In Vitro Validation: Co-culture of key bacterial strains (e.g., *Staphylococcus spp*.) with fibroblasts, followed by qPCR detection of collagen-related genes.
- Animal Model Validation: Murine burn models colonized with scar-associated microbiota; histological scoring to evaluate tissue remodeling and inflammation.

### **SAP05: Procedures for Predictive Model Construction and Validation**

Scope: Principal Investigators (PIs) at clinical centers, Sub-Investigators (Sub-Is), sampling staff, sample quality control staff, central laboratory staff.

Purpose: To standardize the processes for developing, validating, and deploying predictive models of sepsis risk and scar severity. This SAP ensures compliance with the TRIPOD (Transparent Reporting of a multivariable prediction model for Individual Prognosis or Diagnosis) statement, supporting clinical decision-making and reproducible machine learning analyses.

**Definition of Predictive Objectives**

- **Sepsis Risk Model: Predicts the probability of sepsis occurring within the next 72 hours, using data collected within the first 24 hours of admission and incorporating input features such as microbial risk score, SOFA trend, and mHLA-DR level.**
- **Scar Severity Model: Predicts the likelihood of developing severe scarring (VSS ≥ 8) at 6 months post-injury, based on data captured at the time of wound epithelialization, with key input features including wound microbiota composition, inflammatory cytokine levels, and local skin tension.**

Data Preprocessing Standards

Feature Engineering Framework

Raw Microbial Features → Temporal Feature Extraction → Microbial Feature Transformation → Feature Selection

**Key Processing Techniques** **for Predictive Model Construction and Validation**

- **Microbial Composition Data: Processed using CLR Transformation to eliminate compositional bias within the data.**
- **Clinical Time-Series Data: Processed by applying Sliding-Window Statistics to capture underlying temporal dynamics.**
- **High-Dimensional Metagenomics: Processed through LASSO-Based Preselection to achieve dimensionality reduction while maintaining clinical interpretability.**

Feature Selection Criteria

- Mandatory Baseline Features: Age, TBSA (%)
- Microbial Biomarkers: Inclusion criterion |HR| > 1.5 and FDR < 0.1 (Cox model)

Model Construction Workflow

**Algorithm Selection Matrix**

- **Sepsis Risk: Predicted primarily using the XGBoost algorithm, selected for its ability to handle nonlinear interactions, with Cox-Net serving as the alternative method.**
- **Scar Severity: Predicted primarily using the Random Forest algorithm, chosen for its interpretability and capacity for continuous output, with the Generalized Additive Model (GAM) as the alternative method.**

Example: Sepsis Prediction Model

from xgboost import XGBClassifier

from sklearn.preprocessing import StandardScaler

from skopt import BayesSearchCV

import pandas as pd

\# Feature Standardization

scaler = StandardScaler()

X_train = scaler.fit_transform(features)

\# Parameter Optimization (Bayesian Search)

model = XGBClassifier(objective='binary:logistic', tree_method='gpu_hist')

param_space = {'max_depth': (3, 12), 'learning_rate': (0.01, 0.3)}

optimizer = BayesSearchCV(model, param_space, n_iter=50, cv=5)

optimizer.fit(X_train, y_sepsis)

\# Output Feature Importance Ranking

pd.DataFrame(optimizer.best_estimator_.feature_importances_, index=feature_names)

Validation Strategy and Metrics

Alternatively, considering the potential geographic variation in microbiomes, we plan to allocate 20% of patients of each geographical division of China as the respective validation cohort

**Validation Framework**

- Internal Validation: Conducted using 5-fold cross-validation with stratified sampling on the entire dataset, with key metrics including AUC-ROC and Brier Score.
- Temporal External Validation: Performed by splitting data based on enrollment time (80% training, 20% validation), using 20% of data, and employing Calibration Slope as the key metric.
- **Spatial External Validation:** **Utilizes a hold-out set (20%) from independent centers as an independent cohort, with the Net Reclassification Index (NRI) as the key metric.**

Key Performance Metrics

- Discrimination: AUC-ROC ≥ 0.85 (for sepsis model)
- Clinical Utility: Net benefit assessed via Decision Curve Analysis (DCA)

Explainability Standards

Model Interpretation Techniques

- SHAP Values: Applicable to XGBoost / Random Forest models. Output format is a Feature Contribution Waterfall Plot.
- LIME: Applicable to any black-box model. Output format is a Local Surrogate Explanation.
- Partial Dependence Plot (PDP): Applicable to regression-based models. Output format is a Feature–Prediction Relationship Curve.

# **Ethics**

This multicenter, prospective cohort study was conducted in accordance with the ethical principles of the Declaration of Helsinki and the International Council for Harmonisation – Good Clinical Practice (ICH-GCP) guidelines. The study protocol and all associated standard operating procedures were reviewed and approved by the Ethics Committee of the First Affiliated Hospital of Naval Medical University (CHEC2021-081) prior to patient enrollment.

All participating centers signed a unified collaboration and ethics agreement authorizing the collection, transportation, storage, and analysis of biological specimens and clinical data. Each patient, or their legally authorized representative in cases of incapacity, provided written informed consent prior to inclusion. The consent process included information regarding the study objectives, potential risks and benefits, sample handling, data anonymization, and the possibility of future secondary analyses involving microbiome and genomic data.

All biological specimens (skin, stool, oral, and anal swabs) and associated clinical data were anonymized and recorded in the electronic data capture (eCRF) system with unique coded identifiers, ensuring patient confidentiality. Data integration from the Laboratory Information System (LIS) to the eCRF was performed using encrypted, access-controlled interfaces. Only authorized study personnel could access identifiable source data, strictly for verification or monitoring purposes.

The study’s follow-up plan includes regular assessments during hospitalization and up to 12 months post-injury. All follow-up activities were designed to minimize patient burden and adhere to institutional biosafety standards. Sample transportation and cold-chain procedures were carried out under approved biospecimen management protocols, with full traceability maintained through the BIOME-ID coding system.

Any protocol deviations, serious adverse events (SAEs), or breaches of confidentiality were to be reported immediately to the coordinating center’s Ethics Committee, followed by corrective action and documentation according to GCP requirements. No financial compensation or additional interventions beyond standard care were provided to participants.

# **Study Management**

### **Regulatory Documents**

The BIOME multicenter prospective study (Ethics Approval No. CHEC2021-081) was reviewed and approved by the Ethics Committee of the First Affiliated Hospital of Naval Medical University.Prior to study initiation, all participating centers obtained local Institutional Ethics Committee (IEC) approval and signed a standardized inter-center collaboration agreement authorizing biological sample collection, transport, analysis, and data sharing in compliance with the Declaration of Helsinki and Chinese GCP guidelines.

### **Protocol Amendments**

Any amendments to the approved protocol, including updates to Standard Operating Procedures, must comply with the same submission and review procedures as the original protocol.

All revisions, including clarifications of study procedures, additional assessments, or modifications to data management plans, will be submitted to the central and local IEC/IRB as required by applicable regulations.

No changes may be implemented without prior written approval from the IEC/IRB, except in cases where immediate changes are necessary to eliminate potential risks to participants.

### **Data Quality Control and Quality Assurance**

The BIOME study will be managed under Chinese GCP and ICH-GCP quality standards.

Comprehensive data quality assurance will be achieved through:

- Automated data validation via the electronic Case Report Form (eCRF) system.
- Double verification of laboratory data imported from the Laboratory Information System (LIS).
- Regular inter-center consistency checks.
- Periodic quality audits by the Coordinating Center’s Data Management Committee.

See Section 13.4 for on-site monitoring and Section 13.5 for auditing and inspection details.

### **Monitoring**

Designated monitors appointed by the sponsor or coordinating institution will conduct routine monitoring visits (both on-site and remote) to evaluate:

- Recruitment progress and follow-up compliance;
- Accuracy and completeness of eCRF data;
- Adherence to study protocol, SOPs, and GCP standards.

During each visit, investigators and site staff must make all relevant source documents available for review and cooperate fully with monitors.

A monitoring report will be generated after each visit, summarizing observations, deviations, corrective actions, and retraining recommendations.

### **Audit and Inspection**

The sponsor’s clinical quality assurance team or a designated representative may conduct regular audits of the study centers to ensure compliance with protocol and regulatory requirements. Authorized national or local regulatory authorities may perform inspections at any time. Inspectors and auditors will have direct access to all essential documents, including laboratory records, informed consent forms (ICFs), eCRFs, sample tracking logs, and monitoring reports. Investigators must provide full cooperation during such audits or inspections.

### **Source Documents**

Investigators must grant access to all original study documents to regulatory authorities, ethics committee members, or personnel delegated by the sponsor.

Source documents include but are not limited to:

- Signed ICFs,
- Medical and laboratory records,
- Vital signs, imaging, and laboratory test reports,
- Sample tracking logs (including BIOME-ID coding records).

All parties accessing these materials are bound by strict confidentiality obligations regarding participant identity and proprietary information.

### **Case Report Forms**

The sponsor or their authorized data management representative will provide the electronic Case Report Form (eCRF) system and the completion guidelines. Only authorized investigators or delegated personnel may enter data. All data entries must accurately reflect the corresponding source documentation and be entered within 24 hours of collection whenever possible. Data inconsistencies must be justified or corrected following query resolution procedures. Completed eCRFs will be reviewed by the sponsor’s monitoring team to ensure protocol adherence and data integrity.

### **Termination of Study and Termination of Center**

The sponsor retains the right to suspend or terminate the study at any time for any reason, including but not limited to:

- Safety concerns or adverse events indicating potential harm to participants;
- Inadequate enrollment rates;
- Major protocol deviations or noncompliance with GCP.

If a participating center fails to meet quality or enrollment expectations, the sponsor may terminate or replace that center.

- Grounds for center termination include:
- Slow recruitment or protocol violations;
- Incomplete or inaccurate data records;
- Poor communication or delayed reporting;

Failure to maintain biospecimen handling standards or cold-chain integrity.

All investigators will be notified in writing of any study suspension or termination, including reasons and effective dates.

### **Data archiving and Storage**

Investigators must retain all essential study documentation, including but not limited to:

- eCRFs and data validation records,
- Laboratory and LIS reports,
- Sample collection and transportation logs,
- Informed consent forms (ICFs),
- Monitoring and audit reports,
- Serious Adverse Event (SAE) reports and correspondence.

All study-related materials must be stored securely and in an organized manner for a minimum of five (5) years after study completion or early termination.

Records must remain readily available for regulatory inspection upon request.

Any accidental loss or destruction of documentation or samples must be reported to the sponsor immediately.

If the Principal Investigator (PI) retires, relocates, or transfers responsibilities, they must ensure proper handover of all documents and samples to a designated successor and notify the sponsor in writing at least 30 days in advance, providing the successor’s full contact details.

### **Confidentiality**

Investigators and all site staff are required to protect participant confidentiality in compliance with applicable privacy laws and ethical standards. Personal identifiers must not appear on any documents submitted to the sponsor or central data management team. Each participant will be identified only by their unique BIOME Study Number and study-specific code. A separate screening log will be maintained at each site containing identifiable information (e.g., name, address, contact number) for internal use only and will not be shared externally. Investigators must also recognize the sponsor’s proprietary rights over all study materials, including the study protocol, Investigator’s Brochure, SOPs, and eCRF templates. No study-related documents or data may be disclosed to any unauthorized third party without prior written approval from the sponsor. All centers must implement access control and data encryption measures to prevent unauthorized sharing or public disclosure of study information.

# **Data Sharing and Access Policy**

The data that support the findings of this study are available on request from the corresponding authors. The original clinical data are not publicly available due to privacy or ethical restrictions. No new data and scripts were generated in this protocol.

Leveraging the BIOME project as a foundation, we have established a centralized data repository. This initiative marks a critical step toward building a national-level database, fostering a collaborative network that transitions research from single-center studies to nationwide data sharing. The data generated and analyzed during the current study are managed by this centralized repository. The repository adheres to the FAIR (Findable, Accessible, Interoperable, and Reusable) data principles while ensuring strict patient confidentiality. All data-sharing activities are overseen by a Data Access Committee (DAC). To balance open science with privacy, data access is tiered:

- Open Access Data: De-identified metadata, processed microbiome abundance tables, and summary statistics will be publicly available at the project data portal (under construction).
- Controlled Access Data: Raw metagenomic sequencing data (FASTQ files) will be stored in the centralized repository. These data are available to qualified researchers who meet the criteria for access (e.g., for verifying conclusions or conducting authorized secondary analysis). Requests for controlled data should be submitted to the Data Access Committee and require the execution of a Data Use Agreement (DUA).

All participating centers in the BIOME cohort retain co-ownership rights and prioritized access to the dataset for collaborative research and clinical translation.

- Participating centers: As co-constructors of the database, all contributing centers retain unrestricted access to their own contributed data. For research using the pooled, de-identified multicenter dataset, they are granted priority access but must still submit a collaborative research proposal through the standard DAC review process.
- External Access: Access to Controlled Access Data requires submission of a formal research proposal and a completed Data Access Application Form to the DAC. Requests are evaluated based on scientific merit, ethical compliance, and the applicant's data security protocols.

Approved data is transferred via secure, encrypted methods. The Data Access Application Form is attached at the end (please refer to the Appendix for detail).

**BIOME Project Data Access Application Form**

**Instructions:**

This form is required for researchers requesting access to Controlled Access Data (e.g., raw metagenomic sequencing data, high-resolution clinical trajectories) from the BIOME Project. Please complete all sections and submit the signed form to the Data Access Committee (DAC).

**I. Organization and Individual Requesting the Use of Data**

**A. Principal Investigator (PI)**

| Principal Investigator (PI) Name |  |
| --- | --- |
| Title / Position |  |
| Degrees / Credentials |  |
| Phone Number |  |
| Email |  |

**B. Institution / Organization**

| Organization / Institution Name |  |
| --- | --- |
| Department / Unit |  |
| Address |  |

**C. Contact Person (If different than PI listed in I_A_)**

| Contact Person Name |  |
| --- | --- |
| Phone Number |  |
| Email |  |

**D. Co-Investigators (if any)**

*(Name, Affiliation, Email)*

**II. Research Project Description**

**A. Title of Project**

**B.** **Research Abstract** **(approx. 250 words)**

*(Please briefly describe the background, objectives, and expected outcomes of the study.)*

**C.** **Scientific Rationale & Methodology (approx. 500-1000 words)**

*(Please articulate: 1.the specific research gaps the data will address; 2. methods and analytical approaches you plan to use; 3. the potential scientific or translational impact of the work.,)*

**D. Data Requested**

Please specify the data types and time points required for your study:

•Data Types:

◯ Raw Metagenomic Sequencing Data (FASTQ/BAM files)

◻Skin Microbiome	◻Gut Microbiome	◻Oral Microbiome

◯ Detailed Clinical Trajectories (Longitudinal physiological parameters, detailed medication records, etc.)

◯ Biospecimen Metadata

•Time Points of Interest:

◯ Acute Phase (Day 1-3)

◯ Infection/Hypermetabolic Phase (Day 7-28)

◯ Rehabilitation/Scarring Phase (3-12 Months)

◯ All Available Time Points

•Other:

**III. Data Security and Ethical Use Agreement**

The Principal Investigator hereby, on behalf of myself and the research team under my supervision, formally accepts and undertakes to comply with all the following terms:

1.	Non-Identification: The Applicant will not attempt to identify or contact any individual participant from whom the data were derived.

2.	Purpose Limitation: The data will be used solely for the research purpose described in Part II. Any new research objectives require a new application.

3.	Data Security: The Applicant will maintain the data in a secure environment (encrypted servers/local storage) and will not distribute, sell, or share the data with unauthorized third parties.

4.	Citation & Acknowledgement: The Applicant agree to acknowledge the BIOME project in all oral and written presentations, disclosures, and publications resulting from any analyses of these data.

5.	Reporting: The Applicant shall immediately report any data security incidents or inadvertent re-identification of participants to the DAC (within 72 hours upon discovery).

6.	Data Disposal: Upon completion of the approved research project, or upon request, the Applicant will securely and permanently delete/destroy all local copies of the data obtained.

The Applicant understands and agrees that any breach of the above terms constitutes a material breach of this Statement, which will result in immediate termination of data access privileges and may lead to legal and accountability procedures.

**IV: Signatures**

**A. Principal Investigator (Applicant)**

I confirm that I have communicated the full contents of this Statement to all research team members who will handle the data, and I accept responsibility for their compliance.

Printed Name: ____________________	Title: ______________________

Signature: ______________________	Date: _______________________

**B. Institutional Representative (e.g., Dean, Department Head, or Research Office):**

I certify that the Applicant is affiliated with this Institution and that the Institution will oversee the applicant's compliance with data use policies.

Printed Name: ____________________	Title: ______________________

Signature: ______________________	Date: _______________________

--------------------------------------------------------------------------------------------------------------------

**C. For DAC Use Only:**

Application ID： _____________________________

Status: ◻ Approved	◻ Rejected	◻Revision Required

Date: __________________________________

# **References**

1. Chinese Burn Association. The National Burn Conference in Shanghai in 1970. https://www.doctor-network.com/Public/LittleTools/338.html

2. Mock, Charles, Michael Peck, Margie Peden, Etienne Krug, Rajeev Ahuja, Hendrina Albertyn, Welsly Bodha, et al. 2008. 'A WHO plan for burn prevention and care', World Health Organization, Geneva. https://www.who.int/publications/i/item/9789241596299

3. Sanjar, Fatemeh, Alan J. Weaver, Trent J. Peacock, Jesse Q. Nguyen, Kenneth S. Brandenburg, Kai P. Leung. 2020. “Identification of Metagenomics Structure and Function Associated With Temporal Changes in Rat (Rattus norvegicus) Skin Microbiome During Health and Cutaneous Burn.” *Journal of Burn Care and Research* 41: 347−358. https://doi.org/10.1093/jbcr/irz165

4. Costantini, Todd W., Brian P. Eliceiri, Carrie Y. Peterson, William H. Loomis, James G. Putnam, Andrew Baird, Paul Wolf, et al. 2010. “Quantitative assessment of intestinal injury using a novel in vivo, near-infrared imaging technique.” *Molecular Imaging* 9: 30−39. https://doi.org/10.2310/7290.2010.00001

5. Walsh, Dana M., Shaun D. McCullough, Scott Yourstone, Samuel W. Jones, Bruce A. Cairns, Corbin D. Jones, Ilona Jaspers, et al. 2017. “Alterations in airway microbiota in patients with PaO2/FiO2 ratio ≤ 300 after burn and inhalation injury.” *PLOS ONE* 12: e0173848. https://doi.org/10.1371/journal.pone.0173848

6. Huang, Runzhi, Yuntao Yao, Xirui Tong, Lei Wang, Weijin Qian, Jianyu Lu, Wei Zhang, et al. 2023. “Tracing the evolving dynamics and research hotspots of microbiota and immune microenvironment from the past to the new era.” *Microbiology Spectrum* 11: e0013523. https://doi.org/10.1128/spectrum.00135-23

7. de Porto, Alexander P., Nicholas P. Dylla, Matthew Stutz, Huaiying Lin, Maryam Khalid, Michael W. Mullowney, Jessica Little, et al. 2025. “Fecal metabolite profiling identifies critically ill patients with increased 30-day mortality.” *Science Advances* 11: eadt1466. https://doi.org/10.1126/sciadv.adt1466

8. Harris-Tryon, Tamia A., Elizabeth A. Grice. 2022. “Microbiota and maintenance of skin barrier function.” *Science* 376: 940−945. https://doi.org/10.1126/science.abo0693

9. Uberoi, Aayushi, Amelia McCready-Vangi, Elizabeth A. Grice. 2024. “The wound microbiota: microbial mechanisms of impaired wound healing and infection.” *Nature Reviews Microbiology* 22: 507−521. https://doi.org/10.1038/s41579-024-01035-z

10. Mahmud, Md Rayhan, Sharmin Akter, Sanjida Khanam Tamanna, Lincon Mazumder, Israt Zahan Esti, Sanchita Banerjee, Sumona Akter, et al. 2022. “Impact of gut microbiome on skin health: gut-skin axis observed through the lenses of therapeutics and skin diseases.” *Gut Microbes* 14: 2096995. https://doi.org/10.1080/19490976.2022.2096995

11. Dokoshi, Tatsuya, Yang Chen, Kellen J. Cavagnero, Gibraan Rahman, Daniel Hakim, Samantha Brinton, Hana Schwarz, et al. 2024. “Dermal injury drives a skin to gut axis that disrupts the intestinal microbiome and intestinal immune homeostasis in mice.” *Nature Communications* 15: 3009. https://doi.org/10.1038/s41467-024-47072-3

12. Chen, Li-Jian, Yi Liu, Jing-Wen Yang, Yan Lin, Clare Hsu, Kai-Kai Zhang, Jia-Li Liu, et al. 2023. “Microbial community succession in the intestine of mice with deep partial-thickness burns.” *Frontiers in Microbiology* 14: 1140440. https://doi.org/10.3389/fmicb.2023.1140440

13. Sanjar, Fatemeh, Alan J. Weaver, Trent J. Peacock, Jesse Q. Nguyen, Kenneth S. Brandenburg, Kai P. Leung. 2020. “Temporal shifts in the mycobiome structure and network architecture associated with a rat (Rattus norvegicus) deep partial-thickness cutaneous burn.” *Medical Mycology* 58: 107−117. https://doi.org/10.1093/mmy/myz030

14. Pei, Fei, Ren-Qi Yao, Chao Ren, Soheyl Bahrami, Timothy R. Billiar, Irshad H. Chaudry, De-Chang Chen, et al. 2022. “Expert consensus on the monitoring and treatment of sepsis-induced immunosuppression.” *Military Medical Research* 9: 74. https://doi.org/10.1186/s40779-022-00430-y

15. Belkaid, Yasmine, Timothy W. Hand. 2014. “Role of the microbiota in immunity and inflammation.” *Cell* 157: 121−141. https://doi.org/10.1016/j.cell.2014.03.011

16. Cai, Jie, Lulu Sun, Frank J. Gonzalez. 2022. “Gut microbiota-derived bile acids in intestinal immunity, inflammation, and tumorigenesis.” *Cell Host & Microbe* 30: 289−300. https://doi.org/10.1016/j.chom.2022.02.004

17. Hou, Kaijian, Zhuo-Xun Wu, Xuan-Yu Chen, Jing-Quan Wang, Dongya Zhang, Chuanxing Xiao, Dan Zhu, et al. 2022. “Microbiota in health and diseases.” *Signal Transduction and Targeted Therapy* 7: 135. https://doi.org/10.1038/s41392-022-00974-4

18. Greenhalgh, David G., Jeffrey R. Saffle, James H. Holmes IV, Richard L. Gamelli, Tina L. Palmieri, Jureta W. Horton, Ronald G. Tompkins, et al. 2007. “American Burn Association consensus conference to define sepsis and infection in burns.” *Journal of Burn Care and Research* 28: 776−790. https://doi.org/10.1097/BCR.0b013e3181599bc9

19. Integrative HMP (iHMP) Research Network Consortium. 2019. “The Integrative Human Microbiome Project.” *Nature* 569: 641−648. https://doi.org/10.1038/s41586-019-1238-8

20. Gilbert, Jack A., Martin J. Blaser, J. Gregory Caporaso, Janet K. Jansson, Susan V. Lynch, Rob Knight. 2018. “Current understanding of the human microbiome.” *Nature Medicine* 24: 392−400. https://doi.org/10.1038/nm.4517

21. Fan, Yong, Oluf Pedersen. 2021. “Gut microbiota in human metabolic health and disease.” *Nature Reviews Microbiology* 19: 55−71. https://doi.org/10.1038/s41579-020-0433-9

22. Byrd, Allyson L., Yasmine Belkaid, Julia A. Segre. 2018. “The human skin microbiome.” *Nature Reviews Microbiology* 16: 143−155. https://doi.org/10.1038/nrmicro.2017.157

23. Belkaid, Yasmine, Samira Tamoutounour. 2016. “The influence of skin microorganisms on cutaneous immunity.” *Nature Reviews Immunology* 16: 353−366. https://doi.org/10.1038/nri.2016.48

24. Capone, Kimberly A., Scot E. Dowd, Georgios N. Stamatas, Janeta Nikolovski. 2011. “Diversity of the human skin microbiome early in life.” *Journal of Investigative Dermatology* 131: 2026−2032. https://doi.org/10.1038/jid.2011.168

25. Sanford, James A., Richard L. Gallo. 2013. “Functions of the skin microbiota in health and disease.” *Seminars in Immunology* 25: 370−377. https://doi.org/10.1016/j.smim.2013.09.005

26. Brusselaers, Nele, Stan Monstrey, Dirk Vogelaers, Eric Hoste, Stijn Blot. 2010. “Severe burn injury in Europe: a systematic review of the incidence, etiology, morbidity, and mortality.” *Critical Care* 14: R188. https://doi.org/10.1186/cc9300

27. Jeschke, Marc G., Margriet E. van Baar, Mashkoor A. Choudhry, Kevin K. Chung, Nicole S. Gibran, Sarvesh Logsetty. 2020. “Burn injury.” *Nature Reviews Disease Primers* 6: 11. https://doi.org/10.1038/s41572-020-0145-5

28. Legrand, Matthieu, Audra T. Clark, Javier A. Neyra, Marlies Ostermann. 2024. “Acute kidney injury in patients with burns.” *Nature Reviews Nephrology* 20: 188−200. https://doi.org/10.1038/s41581-023-00769-y

29. Deitch, Edwin A. 2012. “Gut-origin sepsis: evolution of a concept.” *The Surgeon* 10: 350−356. https://doi.org/10.1016/j.surge.2012.03.003

30. Church, Deirdre, Sameer Elsayed, Owen Reid, Brent Winston, Robert Lindsay. 2006. “Burn wound infections.” *Clinical Microbiology Reviews* 19: 403−434. https://doi.org/10.1128/cmr.19.2.403-434.2006

31. Huang, Runzhi, Yuwei Lu, Minghao Jin, Yifan Liu, Mengyi Zhang, Shuyuan Xian, Zhengyan Chang, et al. 2023. “A bibliometric analysis of the role of microbiota in trauma.” *Frontiers in Microbiology* 14: 1091060. https://doi.org/10.3389/fmicb.2023.1091060

32. Zhu, Yushu, Yizhen Lin, Sujie Xie, Mingxuan Yang, Wei Zhang, Minjuan Wu, Yifan Liu, et al. 2025. “Mapping intellectual structures and research hotspots of chronic wound in global perspective.” *Regenerative Therapy* 30: 47−62. https://doi.org/10.1016/j.reth.2025.05.002

33. Yan, Lu, Guanwen Liu, Bin Zhao, Bing Pang, Wanqin Wu, Chongyang Ai, Xixi Zhao, et al. 2020. “Novel Biomedical Functions of Surfactin A from Bacillus subtilis in Wound Healing Promotion and Scar Inhibition.” *Journal of Agricultural and Food Chemistry* 68: 6987−6997. https://doi.org/10.1021/acs.jafc.0c01658

34. Huang, Runzhi, Yuntao Yao, Sujie Xie, Junqiang Li, Wei Zhang, Yifan Liu, Shuyuan Xian, et al. 2025. “Optimal Timing and Approaches for First Tangential Excision in Patients with ≥70% TBSA Burns: Insights from a Retrospective Cohort of a Mass Casualty Incident.” *Annals of Surgery* https://doi.org/10.1097/sla.0000000000006626

35. Li, Heng, Richard Durbin. 2009. “Fast and accurate short read alignment with Burrows-Wheeler transform.” *Bioinformatics* 25: 1754−1760. https://doi.org/10.1093/bioinformatics/btp324

36. Li, Dinghua, Ruibang Luo, Chi-Man Liu, Chi-Ming Leung, Hing-Fung Ting, Kunihiko Sadakane, Hiroshi Yamashita, et al. 2016. “MEGAHIT v1.0: A fast and scalable metagenome assembler driven by advanced methodologies and community practices.” *Methods* 102: 3−11. https://doi.org/10.1016/j.ymeth.2016.02.020

37. Hyatt, Doug, Gwo-Liang Chen, Philip F. Locascio, Miriam L. Land, Frank W. Larimer, Loren J. Hauser. 2010. “Prodigal: prokaryotic gene recognition and translation initiation site identification.” *BMC Bioinformatics* 11: 119. https://doi.org/10.1186/1471-2105-11-119

38. Li, Weizhong, Adam Godzik. 2006. “Cd-hit: a fast program for clustering and comparing large sets of protein or nucleotide sequences.” *Bioinformatics* 22: 1658−1659. https://doi.org/10.1093/bioinformatics/btl158

39. Li, Heng, Bob Handsaker, Alec Wysoker, Tim Fennell, Jue Ruan, Nils Homer, Gabor Marth, et al. 2009. “The Sequence Alignment/Map format and SAMtools.” *Bioinformatics* 25: 2078−2079. https://doi.org/10.1093/bioinformatics/btp352

40. Buchfink, Benjamin, Chao Xie, Daniel H. Huson. 2015. “Fast and sensitive protein alignment using DIAMOND.” *Nature Methods* 12: 59−60. https://doi.org/10.1038/nmeth.3176

41. Arumugam, Manimozhiyan, Jeroen Raes, Eric Pelletier, Denis Le Paslier, Takuji Yamada, Daniel R. Mende, Gabriel R. Fernandes, et al. 2011. “Enterotypes of the human gut microbiome.” *Nature* 473: 174−180. https://doi.org/10.1038/nature09944

42. Abed, Raeid M. M., Samiha Al-Kharusi, Stephane Prigent, Tom Headley. 2014. “Diversity, distribution and hydrocarbon biodegradation capabilities of microbial communities in oil-contaminated cyanobacterial mats from a constructed wetland.” *PLOS One* 9: e114570. https://doi.org/10.1371/journal.pone.0114570

43. Bialek, Katarzyna, Amit Kumar, Thérèse Mahony, Piet N. L. Lens, Vincent O'Flaherty. 2012. “Microbial community structure and dynamics in anaerobic fluidized-bed and granular sludge-bed reactors: influence of operational temperature and reactor configuration.” *Microbial Biotechnology* 5: 738−752. https://doi.org/10.1111/j.1751-7915.2012.00364.x

44. Marcondes, Marta Angela, Andrezza Nascimento, Rodrigo Pessôa, Jefferson Russo Victor, Alberto José da Silva Duarte, Patricia Bianca Clissa, Sabri Saeed Sanabani, et al. 2022. “Characterization of Bacterial Communities from the Surface and Adjacent Bottom Layers of Water in the Billings Reservoir.” *Life (Basel)* 12. https://doi.org/10.3390/life12081280

45. Shannon, Paul, Andrew Markiel, Owen Ozier, Nitin S. Baliga, Jonathan T. Wang, Daniel Ramage, Nada Amin, et al. 2003. “Cytoscape: a software environment for integrated models of biomolecular interaction networks.” *Genome Research* 13: 2498−2504. https://doi.org/10.1101/gr.1239303

46. Xie, Chen, Xizeng Mao, Jiaju Huang, Yang Ding, Jianmin Wu, Shan Dong, Lei Kong, et al. 2011. “KOBAS 2.0: a web server for annotation and identification of enriched pathways and diseases.” *Nucleic Acids Research* 39: W316−322. https://doi.org/10.1093/nar/gkr483

47. Huerta-Cepas, Jaime, Damian Szklarczyk, Kristoffer Forslund, Helen Cook, Davide Heller, Mathias C. Walter, Thomas Rattei, et al. 2016. “eggNOG 4.5: a hierarchical orthology framework with improved functional annotations for eukaryotic, prokaryotic and viral sequences.” *Nucleic Acids Research* 44: D286−293. https://doi.org/10.1093/nar/gkv1248

48. Pattabiraman, Srilakshmi, Tandy Warnow. 2021. “Profile Hidden Markov Models Are Not Identifiable.” IEEE/ACM *Transactions on Computational Biology and Bioinformatics* 18: 162−172. https://doi.org/10.1109/tcbb.2019.2933821

49. Jia, Baofeng, Amogelang R. Raphenya, Brian Alcock, Nicholas Waglechner, Peiyao Guo, Kara K. Tsang, Briony A. Lago, et al. 2017. “CARD 2017: expansion and model-centric curation of the comprehensive antibiotic resistance database.” *Nucleic Acids Research* 45: D566−d573. https://doi.org/10.1093/nar/gkw1004

50. Chen, Lihong, Jian Yang, Jun Yu, Zhijian Yao, Lilian Sun, Yan Shen, Qi Jin. 2005. “VFDB: a reference database for bacterial virulence factors.” *Nucleic Acids Research* 33: D325−328. https://doi.org/10.1093/nar/gki008

51. Urban, Martin, Alayne Cuzick, James Seager, Valerie Wood, Kim Rutherford, Shilpa Yagwakote Venkatesh, Nishadi De Silva, et al. 2020. “PHI-base: the pathogen-host interactions database.” *Nucleic Acids Research* 48: D613−d620. https://doi.org/10.1093/nar/gkz904

52. Mistry, Jaina, Sara Chuguransky, Lowri Williams, Matloob Qureshi, Gustavo A. Salazar, Erik L. L. Sonnhammer, Silvio C. E. Tosatto, et al. 2021. “Pfam: The protein families database in 2021.” *Nucleic Acids Research* 49: D412−d419. https://doi.org/10.1093/nar/gkaa913

53. Fischer, Markus, Michael Knoll, Demet Sirim, Florian Wagner, Sonja Funke, Juergen Pleiss. 2007. “The Cytochrome P450 Engineering Database: a navigation and prediction tool for the cytochrome P450 protein family.” *Bioinformatics* 23: 2015−2017. https://doi.org/10.1093/bioinformatics/btm268
